# Mechanistic Foundations of the Sequential Activation of Methane by Ta^+^: Oxidative Addition, Ring-Opening σ‑Bond Metathesis, and C–C Bond Formation

**DOI:** 10.1021/acs.jpca.5c01569

**Published:** 2025-05-06

**Authors:** Tucker W. R. Lewis, Albert A. Viggiano, Brendan C. Sweeny, Jennifer Meyer, Shaun G. Ard, Nicholas S. Shuman

**Affiliations:** † Air Force Research Laboratory, Space Vehicles Directorate, Kirtland AFB, Albuquerque, New Mexico 87117, United States; ‡ RPTU Kaiserslautern-Landau, Fachbereich Chemie und Forschungszentrum OPTIMAS, Erwin-Schrödinger Str. 52, 67663 Kaiserslautern, Germany

## Abstract

The kinetics of Ta^+^ + CH_4_ and related reactions TaC*_n_* H*_m_* ^+^ + CH_4_ (*n* = 2–4, *m* = *n*, 2*n*, 3*n*) are measured from 300–600 K using a selected-ion flow tube apparatus. Complicated kinetics are analyzed through a novel bootstrapping methodology, and rate constants for 38 unimolecular, bimolecular, and ternary processes are reported at each of the four temperatures. As has been well-established, Ta^+^ efficiently dehydrogenates methane through a non-spin-conserved process. Sequential chemistry leads to the dehydrogenation of up to four methane molecules per tantalum center through the competing processes of TaC*_n_* H*_m_* ^+^ + CH_4_ → TaC_*n*+1_H_*m*+2_^+^ + H_2_ (dehydrogenation) and TaC_*n*+1_H_*m*+4_^+^ (association). Supported by density functional theory calculations, the distinct mechanisms and product structures of the sequential reactions are derived. The activation energy for oxidative insertion of Ta into a C–H bond is well-predicted by a simple heuristic: whether or not the reactant tantalum atom possesses unbound valence electrons of opposite spin. TaCH_2_^+^ is predicted to have a small activation energy for oxidative insertion but can only proceed to dehydrogenation of methane via carbon–carbon bond formation, enabled by three separate intersystem crossing events. The product is determined to be the tantalapropene dihydride cation, not the more intuitive tantalapropane cation, via comparison of measured and calculated thermal dissociation rates. The TaC_2_H_4_^+^ tantalapropene dihydride has a prohibitive barrier to oxidative insertion. It proceeds instead through a ring-opening insertion of the entire tantalapropene moiety into a C–H bond via σ-bond metathesis; the unbroken metallacycle bond acts as a tether, preventing the activated products from separating and allowing for further isomerization, leading to dehydrogenation. This and subsequent dehydrogenation processes occur without carbon–carbon bond formation; no evidence of a tantalabutane or larger metallacycle is found.

## Graphical abstract

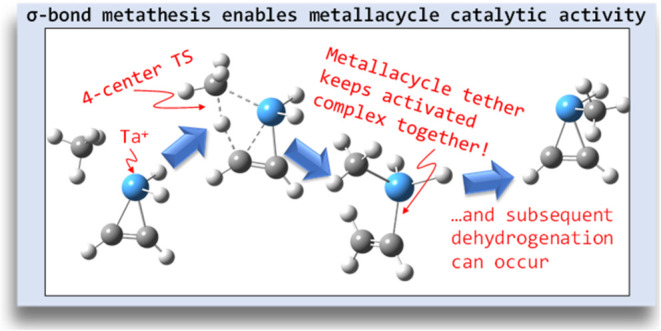

## Introduction

Although activation of the C–H bonds in methane presents a challenge under ambient conditions, the room-temperature dehydrogenation of methane (i.e., loss of H_2_) has been observed in the gas phase through reaction with a variety of transition metal cations. The dehydrogenation reactions are exothermic if the M^+^-CH_2_ bond energy exceeds that of CH_2_–H_2_, i.e., 4.74 eV at 0 K, 4.83 eV at 298.15 K.^1^ This precludes exothermic methane dehydrogenation by all 3d and 4d metal cations as the strongest such bond is 4.63 eV for M = Zr, although both Zr^+^ and Nb^+^ show small activity at room temperature through an endothermic reaction.^2^ The M^+^-CH_2_ bond strengths for 5d atomic cations are generally stronger, ranging from 4.20 eV (M = La) to about 5.3 eV (M = Os); the M^+^-CH_2_ bonding is qualitatively similar for the 3d–5d transition metals, all being covalent double bonds, but quantitatively stronger due to lanthanide contraction of the 6s orbitals resulting in a similar size as the valence 5d improving hybridization.^3–5^ Ta^+^, W^+^, Os^+^, Ir^+^, and Pt^+^ form sufficiently strong bonds to dehydrogenate methane through exothermic processes. In some cases the MCH_2_^+^ product rapidly dehydrogenates a second (Ir^+^), or third and fourth methane (Ta^+^, W^+^).^6,7^ Notably less facile further dehydrogenations can occur, with W^+^ accommodating at least eight carbons and Pt^+^ five.^7,8^ Both thermochemical and coordinative arguments have been made that these sequential processes involve carbon–carbon bond formation, although the product structures are not certain.

The initial reaction of the 5d transition metals with methane has been studied extensively and reviewed.^5^ Ir^+^ and Os^+^ are the most active, reacting at the Langevin-Gioumousis-Stevenson (LGS) capture rate, likely driven by the larger exothermicities of these processes resulting from the larger M^+^-CH_2_ bond dissociation energies (BDE). Pt^+^, Ta^+^, and W^+^ react at significant fractions (20–50%) of the LGS rate. The Ta^+^ system, in particular, has received a large amount of interest, summarized below, which we add to here by focusing on the less well-studied sequential chemistry.

Freiser and co-workers^9^ first reported the reaction

1 $${\mathrm{Ta}}^{+}+{\mathrm{CH}}_{4}\to {{\mathrm{TaCH}}_{2}}^{+}+H_{2},R_{0,0\to 1,2}$$

intuiting an oxidative addition mechanism and identifying sequential activation of up to four methane molecules:

2 $${{\mathrm{TaCH}}_{2}}^{+}+{\mathrm{CH}}_{4}\to {\mathrm{TaC}}_{2}{H_{4}}^{+}+H_{2},R_{1,2\to 2,4}$$

3 $${\mathrm{TaC}}_{2}{H_{4}}^{+}+{\mathrm{CH}}_{4}\to {\mathrm{TaC}}_{3}{H_{6}}^{+}+H_{2},R_{2,4\to 3,6}$$

4 $${\mathrm{TaC}}_{3}{H_{6}}^{+}+{\mathrm{CH}}_{4}\to {\mathrm{TaC}}_{4}{H_{8}}^{+}+H_{2},R_{3,6\to 4,8}$$

where the shorthand R_a,b→c,d_ indicates reaction of TaC_a_H_b_^+^ with methane to yield TaC_c_H_d_^+^. Irikura and Beauchamp followed with consistent results, adding some information on possible structures of sequential products via collision-induced dissociation (CID) measurements.^3,7^ A reaction coordinate was calculated using density functional (DFT) methods.^10,11^ Simon et al. combined ion cyclotron resonance (ICR) experiments with DFT calculations to investigate the sequential reactivity, focusing largely on the second dehydrogenation producing TaC_2_H_4_^+^ and identifying the likely ground state geometry as the tantalapropene dihydride metallacycle H_2_TaC_2_H_2_^+^ not the more intuitive tantalapropane structure.^12^ Note the current IUPAC naming^13^ for metallacycles is preliminary, but recommends following the classic (i.e., Hantzsch–Widman^14^) nomenclature for heterocyclic compounds. In plain language, use the familiar name of the compound if the metal atom were replaced with a saturated carbon atom and alter the “cyclo” prefix to, in this case, “tantala”. Bohme and co-workers surveyed the reactivity of atomic metal cations with methane across the periodic table under thermal conditions, with results consistent with the literature and reported a rate constant of 3.8 × 10^–10^ cm^3^ s^–1^, 39% of the LGS capture rate, for Ta^+^, and observation of the same sequential products up to TaC_4_H_8_^+^.^6^ Armentrout and co-workers reported the reactivity as a function of collision energy using a guided ion beam apparatus, including calculated density functional (DFT) reaction coordinates, relative cross sections for the sequential reactivity, and the reverse TaCH_2_^+^ + H_2_ reaction providing a determination of the Ta–CH_2_^+^ bond dissociation energy (BDE) via the equilibrium constant.^15^ The structure of the TaCH_2_^+^ has been determined using action spectroscopy, confirming a Ta–C double bond along with an agostic interaction between the Ta and a hydrogen atom,^16–19^ along with information on related species including TaC_2_H_2_^+^, which forms the tantalapropene cation metallacycle. The sequential chemistry of Ta*_n_* ^+^ (*n* = 1–10) was studied using an ion trap apparatus, showing continuously decreasing reactivity with increasing cluster size.^20^ More recently, Meyer and co-workers investigated the dynamics of the reaction at suprathermal collision energies via a crossed-beam velocity map imaging experiment.^21^ Guo and co-workers calculated parametrized potentials of the lowest energy singlet, triplet, and quintet surfaces and used quasi-classical trajectory calculations to compare to experimental data, concluding that the intersystem crossing (ISC) is the primary kinetic constraint.^22^ Related chemistry involving tantalum-mediated coupling of methane and carbon dioxide has also been investigated initially by Schwarz and co-workers^23^ and more recently by other groups.^24–26^

The mechanism of the initial activation of a methane molecule by Ta^+^ has been discussed, occurring similarly to that of other M^+^.^15,27–29^ M^+^–H bonds are weaker than a methane C–H bond and the metal cannot directly abstract a hydrogen atom at thermal energies. Instead, the process occurs through oxidative addition followed by reductive elimination of H_2_. After forming a weakly bound entrance complex Ta^+^(CH_4_), the Ta^+^ inserts into a C–H bond by forming covalent Ta–H and Ta–CH_3_ bonds. The process is well-described by a donor–acceptor model,^15^ with the activation energy of the process being small if both a vacant frontier acceptor orbital and a donor orbital are available. For Ta^+^ with just 4 valence electrons (such that there are necessarily unoccupied valence orbitals), the activation energy for oxidative insertion of Ta into a C–H bond will be small if the Ta^+^ possesses unbound valence electrons of opposite spin. One α and one β electron from the Ta ion are needed to form covalent bonds with both species from the homolytically cleaved C–H bond (the fragments from which necessarily have unpaired electrons of opposite spin), favoring reaction on lower spin surfaces. Ta^+^ is a ground-state quintet with all four valence electrons having common spin. The transition state for insertion is above that of reactants on quintet surfaces but is much lower on triplet or singlet surfaces, which have valence electrons of unlike spin available. After insertion, the reaction proceeds via a submerged dehydrogenation barrier, yielding TaCH_2_^+^. Because of the newly formed covalent bonds, the product cation will tend toward a lower spin than the reactant cation. Absent additional rearrangement, reactions of open-shell TaR^+^ species with a closed-shell species, e.g., methane, will in general require an ISC in order to access the multiplicity of the ground state product.

It can be viewed as somewhat unexpected that the Ta^+^ + CH_4_ reaction navigates all these obstacles, requiring a sufficient Ta^+^–CH_2_ bond strength, a low-lying electronic state with available electrons to facilitate the insertion, and a facile ISC to access that electronic state, to not just proceed, but to proceed efficiently. It is more unexpected that reactions 2–4 proceed efficiently as increasing ligation both reduces the Ta^+^–C bond strengths and sequesters the electrons required to activate a C–H bond.

The mechanism(s) of sequential activation of additional methane molecules have been less detailed. Rather than forming multiple carbenes, carbon–carbon bond formation is believed to occur and the sequential products are likely metallacycles, although the nature of each of those products has not been established. While the initial Ta^+^ + CH_4_ dehydrogenation occurs via oxidative insertion, the mechanism(s) for the sequential dehydrogenations have also not been established.

In addition to reactions 1–4, methane association reactions

5 $${{\mathrm{TaC}}_{a,b}}^{+}+{\mathrm{CH}}_{4}\to {\mathrm{TaC}}_{a+1}H_{b+4}$$

can occur, and each of those products has the opportunity for further chemistry with methane, including dehydrogenation. An association reaction followed by a dehydrogenation reaction yields the same molecular formula as the inverted process, although there is no guarantee that the product geometry or electronic state is the same. As a result, the sequential gas phase chemistry initiated by Ta^+^ + CH_4_ is a complicated, interweaved web of reaction paths from which extracting well-defined kinetics is challenging. Here we combine measurements of the sequential chemistry initiated by Ta^+^ and other TaR^+^ + CH_4_ reactions (where R = CH_2_ and C_2_H_2_, i.e., species involved in the sequential chemistry initiated by Ta^+^ + CH_4_) with density functional calculations and a novel bootstrapping analysis to derive the kinetics with reliable uncertainties in order to elucidate product and mechanistic information about the dehydrogenations.

## Methods

### Experimental Section

Temperature-dependent kinetics of Ta^+^ + CH_4_ and the successive reactions were measured using the Variable Ion Source Temperature Adjustable Selected Ion Flow Tube (VISTA-SIFT) instrument located at the Space Vehicle Directorate of the Air Force Research Laboratory. The VISTA-SIFT instrument has been described in detail previously.^30^

Ta^+^ was produced from the frequency-doubled output of a 100 Hz Nd:YAG laser focused onto a rotating and translating Ta rod (ESPI, 99.9% trace metals basis). TaCH_2_^+^ and TaC_2_H_2_^+^ were produced by leaking a small flow of CH_4_ into the source chamber. Efforts to produce TaC_2_H_4_^+^ and TaC_3_H_6_^+^ from the source in sufficient quantities failed; as detailed below, these species are subject to thermal decomposition at temperatures not far above ambient. The resulting plasmas were entrained in an Ar (99.999% Matheson) expansion produced from a Parker Series 9 solenoid valve operating at 100 Hz. Cations were extracted to the entrance of a rectilinear ion guide and transported through a second rectilinear ion guide to a quadrupole bender. Ions were mass selected using a quadrupole mass filter located at the end of the quadrupole bender and transported through a series of five rectilinear ion guides to the stainless-steel reaction flow tube (1 m long, 7.3 cm diameter). Ions were injected into the ∼0.3 Torr flow tube through a Venturi inlet flowing 10–14 std. L min^–1^ helium (99.999% Matheson) undergoing 10^4^–10^5^ collisions with the helium buffer gas prior to reaction. A 1/8” diameter stainless-steel finger inlet located 59 cm from the end of the flow tube was used to introduce CH_4_ into the flow tube metered using a mass flow controller (MKS Inc.). Typical reaction times were 2–3 ms. Ions exited the flow tube through a 4 mm aperture in a rounded, carbon-coated nosecone and were transported to the entrance of an orthogonally accelerated time-of-flight Reflectron mass spectrometer. Ions were detected using a “Z-stack” of microchannel plates and counted using a time-to-digital converter as a function of methane flow.

After injection into the reaction flow tube, reactant ions underwent 10^4^–10^5^ collisions with the helium buffer gas. For polyatomic species, this likely ensured thermalization to the wall temperature of the flow tube, but thermalization was less certain for atomic species, i.e., Ta^+^. The experiment did not directly probe the electronic state of the reactant and thermalization can only be inferred from the observed behavior. Several pieces of circumstantial evidence suggest that the Ta^+^ in the present experiment was thermalized, or nearly so. The decay of the Ta^+^ with reactants (methane here, CO_2_ in a separate experiment^31^ conducted under the same conditions) is well-described by a single exponential over nearly 2 orders of magnitude at all temperatures, suggesting that all Ta^+^ present react with a similar rate constant. That the rate constant is subcollisional and therefore controlled by subtleties of the potential surfaces and subject to the total energy available makes it unlikely that widely differing electronic states were present. Separately, the observed rate constant was consistent with or without a 100 std cm^3^ s^–1^ flow of N_2_ into the flow tube, indicating that either excited states were not present in significant quantities or were unquenched by the N_2_. Finally, the observed kinetics were consistent from day-to-day and under varying ion source conditions, a result that is unlikely if excited state ions were being produced. We conclude that at most a small fraction (<5%) of Ta^+^ differed from the expected thermal distribution of electronic states. It is worth noting that Ta^+^ has four valence electrons and a 5d^3^(^4^F)­6s quintet ground state with low-lying 5d^2^6s^2^ triplet excited states. At room temperature, nearly all (99%) of the Ta^+^ population is in the ground spin–orbit state. The first excited spin-orbit state at 1031 cm^–1^ carries 12% of the thermal population at 600 K, while higher states are essentially unpopulated at these temperatures.

Primary rate constants were measured by varying the neutral CH_4_ concentration while monitoring the depletion of Ta^+^, TaCH_2_^+^, and TaC_2_H_2_^+^. Injection of larger species, particularly TaC_2_H_4_^+^, was attempted, but it was not found possible to produce sufficient quantities using the LaVa source to enable mass-selection. Primary rate constants were measured at 300–600 K by heating the flow tube walls to each temperature and repeating this procedure. Partial rate constants of the primary and subsequent reactions were obtained from the Statistical Kinetic Analysis (SKA) method described below.

### Analysis

Commonly, analysis of ion–molecule kinetics assumes that the most probable fit is also the “best” fit, determined by minimizing the deviation of the model about the data. Rate constant errors in the fitting procedure are often estimated from experience, or from analysis of a covariance matrix, imposing a normal distribution on these errors. Here we take a different approach to build on these past methods and avoid these assumptions.

The Ta^+^ + CH_4_ reaction is initially branched to form TaCH_2_^+^ and TaCH_4_^+^ and each of these products subsequently reacts with CH_4_, branching to a wider set of products, and this process repeats itself 5 times to give a very complicated network of reactions with 38 partial rate constants. To analyze these data and extract partial rate constants with statistically relevant error bars, the SKA method was developed. An earlier version of the method has been described^32^ but has been improved for this study.

A reaction system consisting of all the observed species and the proposed relationships between them is converted to a system of ordinary differential equations (ODEs) and numerically integrated using a Dormand–Prince 5(4) solver for a given set of parameters consisting of the initial concentration of each species and a set of partial rate constants for each reaction. This yields a simulated set of concentrations of each species. A best fit set of parameters is found by minimizing a goodness-of-fit (GOF) function that assigns a numerical value to the difference between the measured concentration of each species for each neutral flow, and the simulated concentrations at the same neutral flows for a given set of parameters. The GOF function is minimized by using the JADE implementation of the differential evolution algorithm to vary the parameters. Good fits for this complicated reaction system can be typically found in 10^6^ parameter set evaluations. The best fit parameters and GOF value are then recorded.

Error determination is done through a bootstrapping method. Using bootstrapping to estimate the errors in the initial concentrations and partial rate constants does not require the assumption of any underlying distribution to the errors. By assuming that the data points in each data set are from a single empirical distribution function, the error in each parameter can be estimated by resampling each data set with replacement, fitting the sample and recording the best-fit parameters, and repeating this procedure to develop the parameter distributions. Simply, a bootstrap sample is generated by selecting a random set, with replacement, of data points from each measurement, and then is fit via differential evolution. The best fit parameters and GOF value for the bootstrap sample are recorded, and then the procedure is repeated 5 × 10^3^ times to build a distribution of initial concentrations of each species and partial rate constants. The width of this distribution is taken as the total error in each parameter.

The differential evolution algorithm is stochastic, and the problem being solved is complicated, so a small number of fits get stuck in local minima and are visibly poor fits. To reject this set of fits a modified Tukey’s Fence method is used to reject outlier GOF values and the related sets of parameters:

6 $$\mathrm{modified}\;\mathrm{Tukey’s}\;\mathrm{Fence:},{\;Q}_{75}+1.5\left(Q_{75}-Q_{50}\right)$$

To test the statistical validity of this method, artificial data was generated from the proposed reaction sets with artificial initial concentrations of each species and partial rate constants. These artificial data sets were fit using the above method, and the resulting error distributions included the correct values >95% of the time. As a result, the errors reported here are conservatively considered 95% confidence intervals.

### Quantum Calculations

All calculations were performed using the Gaussian 16 C.01 quantum chemical software. A large number of stationary points for TaC_*m*_H_*n*_^+^ (*m* = 0–5, *n* = 0–3*m*) isomers on singlet, triplet, and quintet surfaces were identified at the B3LYP/def2-TZVP level.^33^ The def2-TZVP basis set for Ta contains a 60-electron effective core potential. The energetics of transition metal species at this level of theory are not highly accurate. It has been previously noted that the B3LYP functional overestimates bond dissociation energies (BDE) for single bonds in transition metal complexes, but performs much better for multiply bound species.^15^ This is found here as well, with the method overestimating the Ta^+^–CH_3_ and Ta^+^–H BDEs by about 0.4 eV, but deviating from the experimental value of Ta^+^–CH_2_ BDE by just 0.1 eV. The functional does well reproduce the experimentally determined geometries of several Ta^+^ species.^16,18^ For selected species, the electronic energies were refined by extrapolating to the complete basis set (CBS) limit at the CCSD­(T)/CBS (PVXZ, X = T, Q)//B3LYP/def2-TZVP level. PVXZ refers here to the cc-pVXZ basis sets^34^ on carbon and hydrogen atoms, and the cc-pVXZ-PP basis set^35^ on the tantalum atom. The latter includes a small-core 60 electron effective core potential and was obtained from the Basis Set Exchange.^36^ The CBS extrapolation followed the method outlined by Schwenke^37^ using the parameters determined by Neese and Valeev for the cc-PVXZ (X = 3, 4) basis sets.^38^

Calculations were checked for wave function instability. A number of singlet TaR^+^ species showed a restricted-unrestricted instability. The resulting unrestricted calculations yielded biradical singlets, generally at lower energy than the closed shell species found with the restricted calculations. Recalculating selected species using unrestricted CCSD­(T) yielded the closed-shell species, and these are likely preferred.

Minima were confirmed to have no imaginary frequencies, while transition states (TS) were confirmed to have a single imaginary frequency. For several of the smaller species, reaction coordinates with methane were calculated. In these cases, TS were confirmed to connect to intermediates via intrinsic reaction coordinate calculations. Where relevant, natural bond orbital (NBO) analysis was used to identify electron localization. Full calculation results are reported in Supporting Information.

## Results

### Derived Rate Constants from Ta^+^, TaCH_2_^+^, and TaC_2_H_2_^+^ + CH_4_

Example data for selected species is shown in Figure 1 for the injections of Ta^+^, TaCH_2_^+^, and TaC_2_H_2_^+^, along with the simultaneous fits using the reaction network given in Table S1 to the data. Fits to data from each individual injection yields fits and error distributions for the partial rate constants. However, many of these distributions are ill-defined in one set of fits, while being well-defined in others. For example, for the Ta^+^ injection data rate constants are poorly defined for the later steps in the reaction system, while those are better defined for the TaC_2_H_2_^+^ injection data. The error bars for the partial rate constants from the Ta^+^, TaCH_2_^+^, and TaC_2_H_2_^+^ injection data were inclusive and so the data was fit simultaneously, which substantially improved the fitting quality and narrowed the distributions for each partial rate constant. The results presented in Figure 1 and below are from the simultaneous fitting of the data sets from all 3 injections.

**Figure 1:**
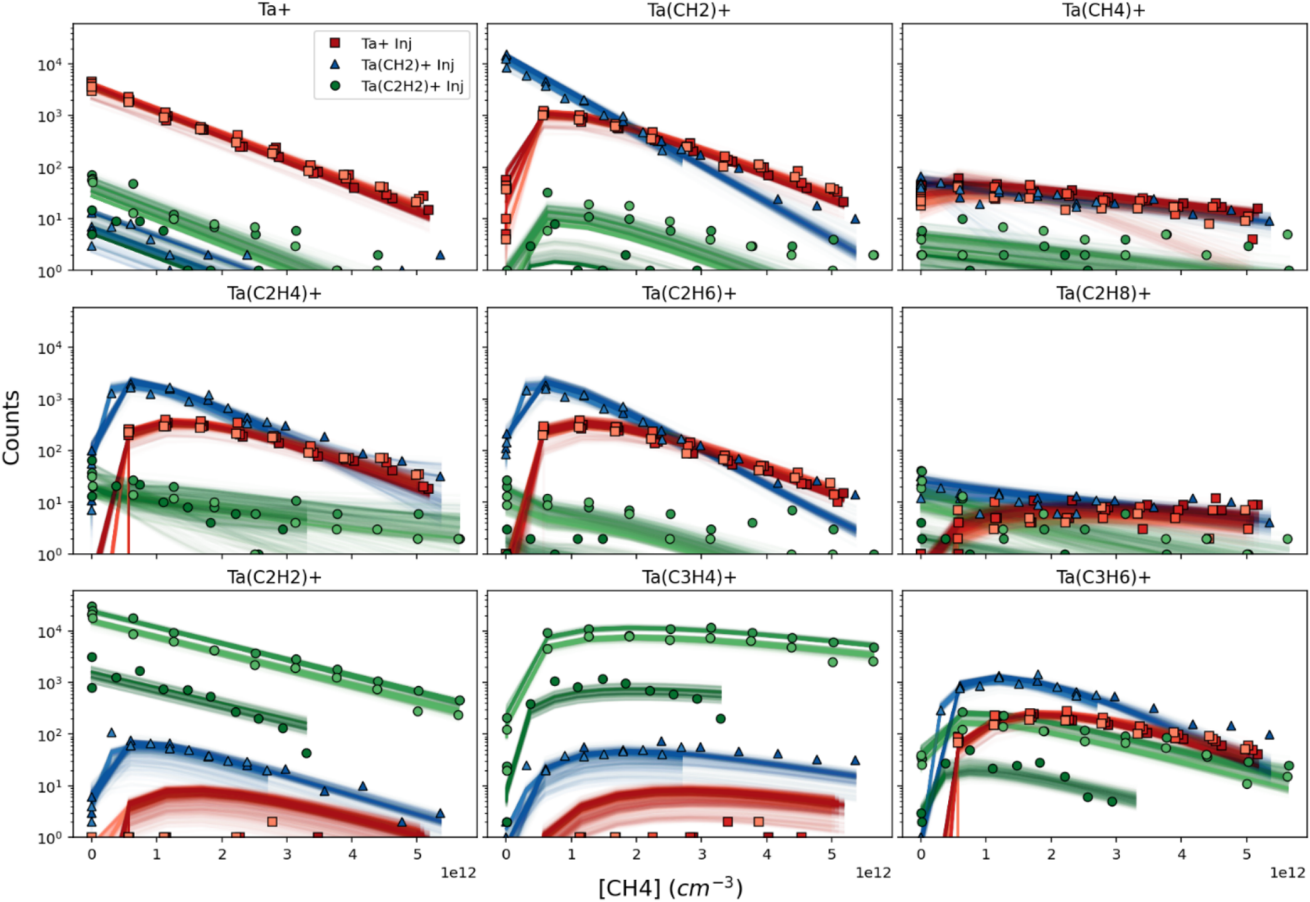
Observed ion abundances from multiple experiments compared to modeled fits. Data from 9 experiments across 3 experimental conditions (injection of either Ta^+^ (red squares), TaCH_2_^+^ (blue triangles), or TaC_2_H_2_^+^ (green circles)) are shown in each panel; individual experiments are distinguished by shade (the initial injected counts varied between experiments within each experimental condition, most notably for TaC_2_H_2_^+^ injection). Curves are modeled abundances for varying sets of rate constants, which reproduced the experimental values within an acceptable likelihood (see text). Data shown here are at 300 K; analogous plots for other temperatures are shown in Figures S3–S6.

Typical fits to the experimental data are shown in Figure 1; all fits are presented in Figure S1. Points are experimental data and solid lines are each of the bootstrap fits to the data. Note that each panel shows the ion abundance from 9 experiments; the more common visualization of all species for a single experiment is shown in Figure S1, but is cluttered due to the large number of species. In general, the fits are excellent, particularly for species with more than 10 counts. Deviations for some of the low count species are observed, particularly for data from the injection of TaC_2_H_2_^+^. Most likely, these deviations indicate minor contributing chemistries that have not been included in the analysis but are unlikely to affect the derivation of rate constants for the dominant processes. Additionally, the spread in the fits closely approximates that of the scatter in the experimental data, indicating that the uncertainty has been appropriately determined.

The derived rate constants as a function of temperature for selected reactions are shown in Figure 2, with those of all other reactions presented in Figures S2–S5. All data points are at 300, 400, 500, or 600 K, with horizontal offsets for clarity to minimize distributions from overlapping. The violin plots shown in Figure 2 show 95% probability intervals for each rate constant. The uncertainty distribution is an output of the analysis, which is shown as the envelope. The horizontal extent is linearly proportional to the likelihood of that value, such that the widest portion indicates the single most likely value. The extreme values along the vertical are comparable to 2σ uncertainty limits. Table S1 represents these derived rate constants as best possible in “typical” fashion, that is a single most likely value along with high and low uncertainty limits.

**Figure 2:**
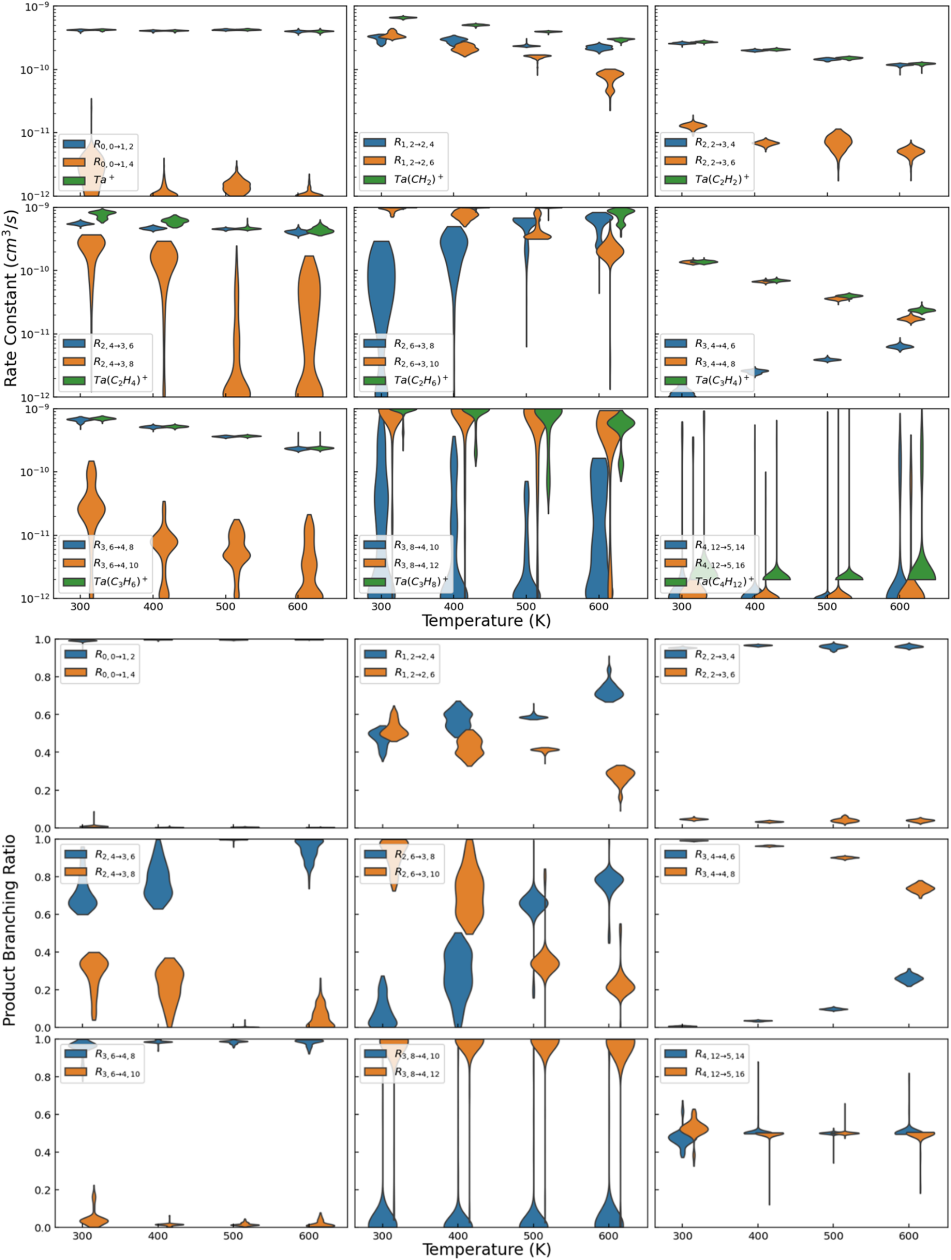
Derived rate constants (top 9 panels) and corresponding product branching fractions (bottom 9 panels) for dehydrogenation reactions (blue), association processes (orange), and the summed total rate constant (green) for the indicated reactions (results for other reactions shown in Figure S6). All measurements are at 300, 400, 500, or 600 K, with the blue and green offset to prevent overlap. The envelopes are symmetric about the vertical, with the width being proportional to the likelihood of that value.

The reaction network and measured rate constants are summarized in Figure 3. Pathways are elucidated and rate constants and uncertainties are summarized as follows.

**Figure 3:**
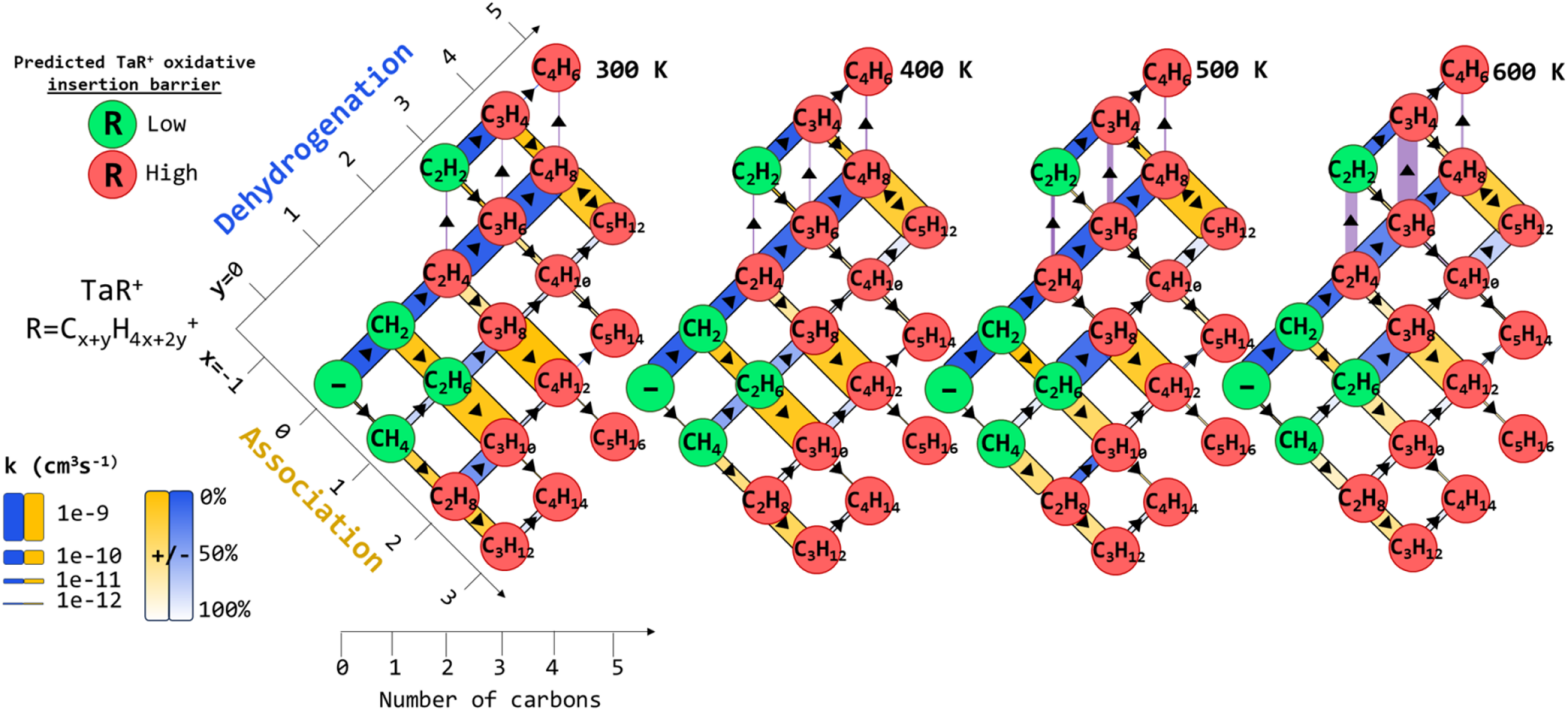
Visualization of the reaction network initiated by Ta^+^ + CH_4_. Circles represent a TaR^+^ species by indicating the R group (“–” indicates a bare Ta^+^). The connecting lines indicate either bimolecular dehydrogenation (blue), association (orange), or unimolecular thermal dissociation (red) processes, with the thickness proportional to the rate constant on the indicated log scale. The purple vertical arrows indicate thermal dissociation (see text). The transparency of the connecting lines represents the uncertainty in the derived rate constant. The color of each circle represents whether the predicted activation energy for insertion of the Ta^+^ into a C–H bond is low (green) or high (red).

R_0,0→1,2_ (i.e., Ta^+^ + CH_4_ → TaCH_2_^+^ + H_2_) proceeds with a nearly temperature-independent (*T* ^0.0±0.2^) rate constant of 4 × 10^–10^ cm^3^ s^–1^. The room temperature rate constant is in agreement with literature values.^6,15,20^ This compares reasonably to the GIBMS experiment of Armentrout and co-workers at the lowest reported kinetic energies.^15^ The competing association reaction R_0,0→1,4_ occurs inefficiently under the present experimental conditions.

The sequential dehydrogenation processes (R_1,2→2,4_, R_2,4→3,6_, R_3,6→4,8_) occur with room temperature rate constants of a similar magnitude (ranging from 2.5 × 10^–10^ to 6.5 × 10^–10^ cm^3^ s^–1^) with increasingly steep negative temperature dependences, *T* ^–0.15±0.2^, *T* ^–0.4±0.2^, *T* ^–1.6±0.2^, respectively. These species also cluster more readily than does Ta^+^, with room temperature rate constants for R_1,2→2,6_, R_2,4→3,8,_ R_3,6→4,10_ of 3.4 × 10^–10^ cm^3^ s^–1^, 1.2 × 10^–10^ cm^3^ s^–1^, and 2.2 × 10^–11^ cm^3^ s^–1^, respectively, all with steep negative temperature dependences characteristic of association reactions.^39^

The sequential dehydrogenations terminate after four iterations, with R_4,8→5,10_ not occurring. Instead, association R_4,8→5,12_ occurs efficiently at all temperatures, but the thermal dissociation of TaC_5_H_12_^+^ to yield TaC_4_H_8_^+^ + CH_4_ occurs with an increasingly large rate constant with temperature, limiting the observed abundance of TaC_5_H_12_^+^.

For several species and rate constants, significant discrepancies exist between the data sets starting with Ta^+^, TaCH_2_^+^, or TaC_2_H_2_^+^ unless thermal dissociation of select species is considered in the reaction network. For 300 K, under typical flow tube conditions, species bound by less than about ∼0.5 eV are unlikely to have ms or longer lifetimes and are unlikely to be observed on the time scale of this experiment. Species bound by about 0.5 to 1 eV are likely to have lifetimes on the ms scale and will undergo a measurable amount of decay due to this process on the time scale of the experiment.

An example of the above is that TaC_2_H_2_^+^ is observed, particularly at higher temperatures. This implies that either

7 $${{\mathrm{TaCH}}_{2}}^{+}+{\mathrm{CH}}_{4}\to {\mathrm{TaC}}_{2}{H_{2}}^{+}+2H_{2}$$

or

8 $${\mathrm{TaC}}_{2}{H_{4}}^{+}+\mathrm{He}\to {\mathrm{TaC}}_{2}{H_{2}}^{+}+H_{2}+\mathrm{He}$$

is occurring, the latter being an example of thermal dissociation. Guided ion beam measurements^15^ that sampled the reaction at low pressures, room temperature internal energies, and a range of kinetic energies (to well above the total energies sampled here) do not report the TaC_2_H_2_^+^ product, supporting that it is formed here by thermal dissociation, which cannot occur in the near single collision GIB experiment. By including thermal dissociation processes, the discrepancies between data sets injecting Ta^+^, TaCH_2_^+^, or TaC_2_H_2_^+^ are resolved, producing the high-quality fits shown in Figure 1.

The effective unimolecular rate constant of the thermal dissociation (i.e., TaR^+^ → Ta­(R – H_2_)^+^ + H_2_) may be predicted using statistical theory to model the reverse association process and thermodynamics.^40^ The thermal dissociation rate constants provide information on the bond dissociation energy and therefore on the structure of the dissociating species. Figure 4 shows a comparison of derived thermal dissociation rates to those calculated for isomers of the indicated species using energies calculated at the CCSD­(T)/CBS level, with additional details in SI.

**Figure 4:**
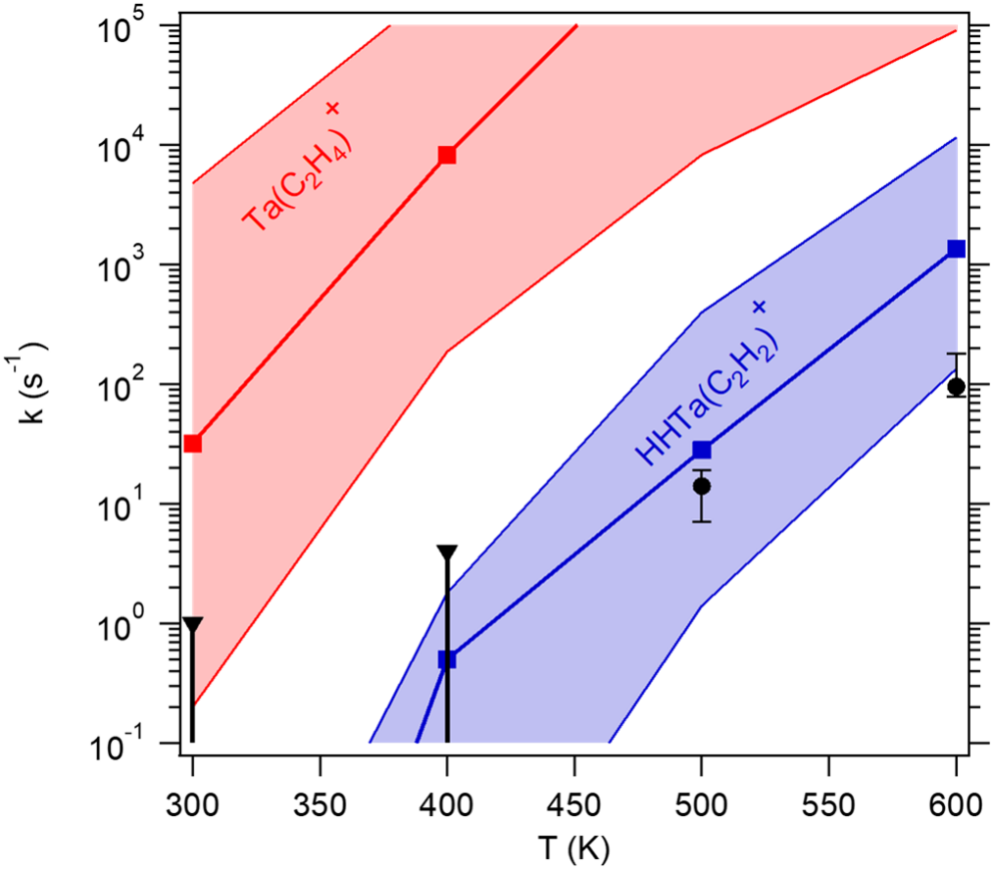
Thermal dissociation rate constants for TaC_2_H_4_^+^ as a function of temperature derived from experiment (solid black points, triangles are upper limits) compared to those calculated using statistical theory (see text) assuming either the tantalapropane cation (red squares and shaded area) or the tantalapropene dihydride cation (blue squares and shaded area); uncertainty limits in the calculated values are derived assuming an uncertainty of ±0.15 eV in the calculated TaC_2_H_2_^+^–H_2_ bond strength.

The calculated values assuming the tantalapropene dihydride (ground state isomer, Figure 6A) cation agree well with the experimental values at 500 and 600 K and with the measured upper limits at 300 and 400 K. The calculated values assuming the tantalapropane cation (Figure 6C) are orders of magnitude faster than the experimental values at all temperatures, suggesting that the isomer would not be observable on the ms time scale of the experiment.

Where comparison is possible, the present room temperature rate constants agree reasonably well with prior results (Table 1).

**Table 1:** Reaction Rate Constants of Indicated Species with Methane

	*k*_+CH_4_/–H_2__ (×10^–10^ cm^3^ s^–1^)				
species	present results (300 K)	Simon et al.^12^	Eckhard et al.^20^	Irikura and Beauchamp^7^	Shayesteh et al.^6^
Ta^+^	4.2 ± 0.8	fixed at 3.9	3.8 ± 0.8	3.4 ± 0.9	3.8 ± 1.0
TaCH_2_^+^	3.2 ± 0.8	3.3	3.5 ± 0.7	2.0 ± 0.5	∼4
TaC_2_H_4_^+^	5.0 ± 1.1	1.7	4.0 ± 0.8	2.0 ± 0.5	∼4
TaC_3_H_6_^+^	8.0 ± 1.5	1.8	5.5 ± 1.1	1.4 ± 0.3	∼4
TaC_4_H_8_^+^	–	–	–	–	

### Thermochemistry

A number of experimental and calculated values have been reported for the exothermicity of Ta^+^ + CH_4_ → TaCH_2_^+^ + H_2_.^4,10,15,41^ Because the CH_2_–H_2_ BDE is accurately known,^1^ the reaction exothermicity determination is also a determination of the Ta–CH_2_^+^ BDE. Therefore, we have studied the reverse process TaCH_2_^+^ + H_2_ → Ta^+^ + CH_4_ at 300–600 K using the SIFT technique (Table 2). Using literature thermochemistry, the measured equilibrium constants imply a 0 K reaction thermicity of – 0.07 ± 0.04 eV (see SI for detailed derivation). It is worth noting that the enthalpy of reaction becomes increasingly less negative with temperature, becoming endothermic around 600 K; however, the free energy of reaction remains negative. The derived Ta^+^–CH_2_ BDE (4.81 ± 0.04 eV) agrees with that derived from GIBMS measurements (4.81 ± 0.03 eV).^15^

**Table 2:** Measured Rate Constants for TaCH_2_^+^ + H_2_ → Ta^+^ + CH_4_ and Corresponding Derived Equilibrium Constant for Ta^+^ + CH_4_ ⇄ TaCH_2_^+^ + H_2_

*T* (K)	*k* (cm^3^ s^–1^)	*K*
300	(8.4 ± 2) × 10^–13^	452 ± 95
400	(1.3 ± 0.2) × 10^–12^	292 ± 45
500	(1.6 ± 0.4) × 10^–12^	259 ± 30
600	(1.6 ± 0.3) × 10^–12^	248 ± 50

A large number of TaC*_n_* H*_m_* ^+^ structures were calculated at the B3LYP/def2-TZVP level. A selection of lower energy isomers relevant to the discussion below are shown in Figures 5–11, with more results in SI.

**Figure 5:**
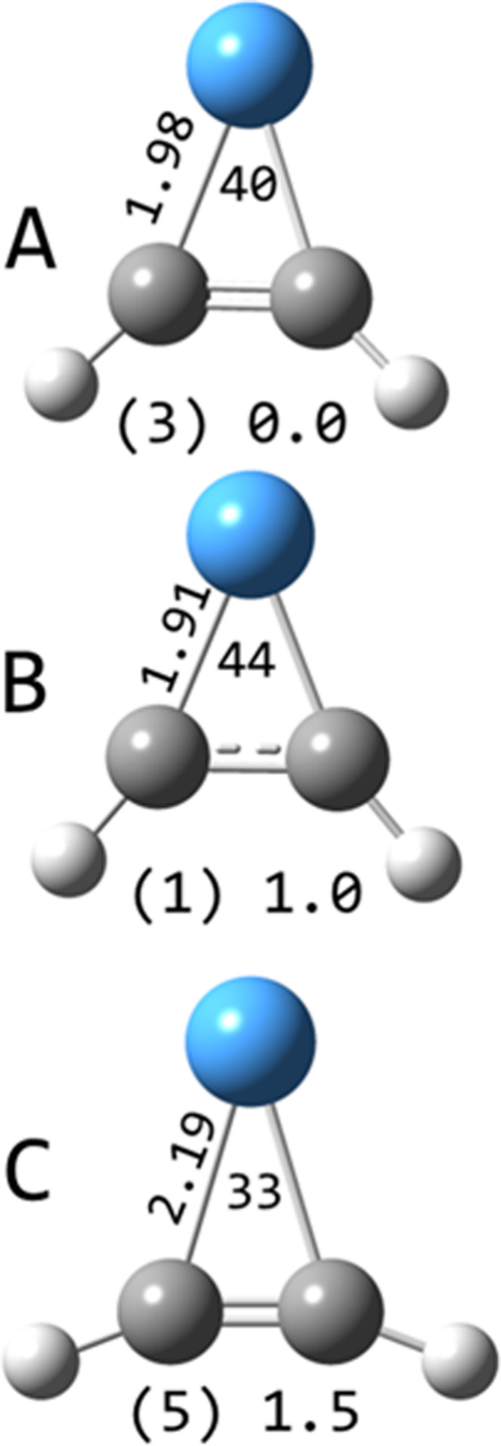
Selected structures of TaC_2_H_2_^+^ calculated at the B3LYP/def2-TZVP level. Multiplicities in parentheses and relative energies in eV are indicated along with Ta–C bond lengths (Å) and C–Ta–C angles (degrees).

**Figure 6:**
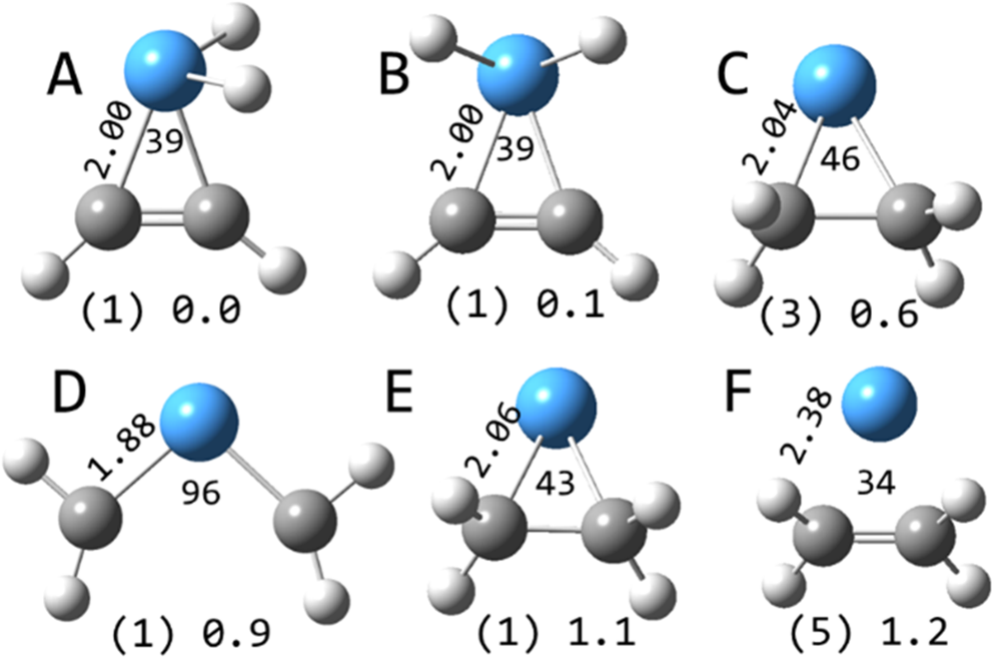
Selected structures of TaC_2_H_4_^+^ calculated at the B3LYP/def2-TZVP level. Multiplicities in parentheses and relative energies in eV are indicated along with Ta–C bond lengths (Å) and C–Ta–C angles (degrees).

**Figure 7:**
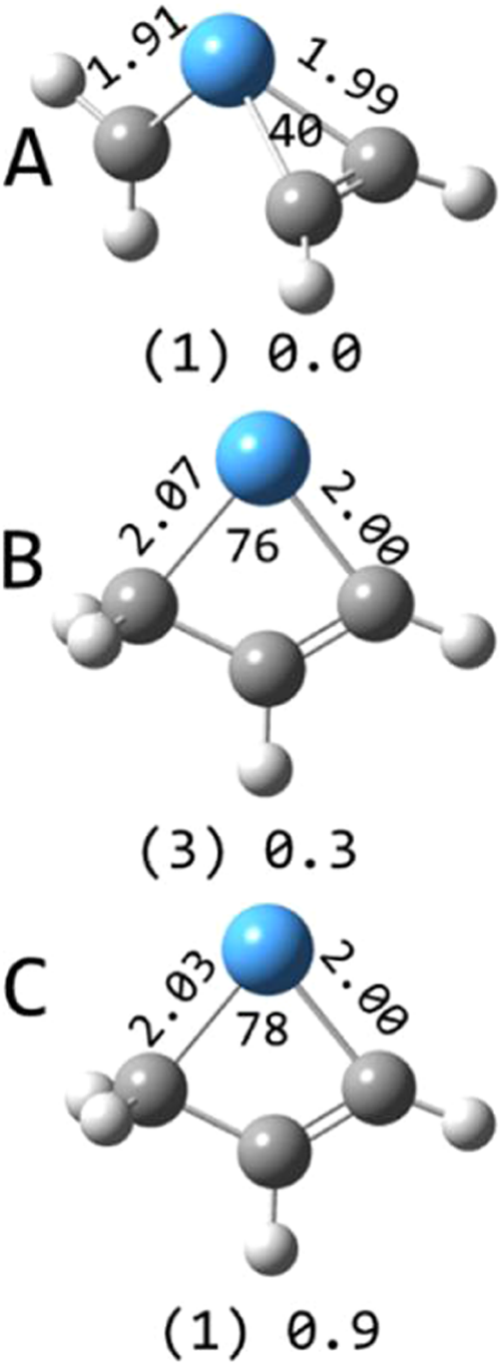
Selected structures of TaC_3_H_4_^+^ calculated at the B3LYP/def2-TZVP level. Multiplicities in parentheses and relative energies in eV are indicated along with Ta–C bond lengths (Å) and C–Ta–C angles (degrees).

**Figure 8:**
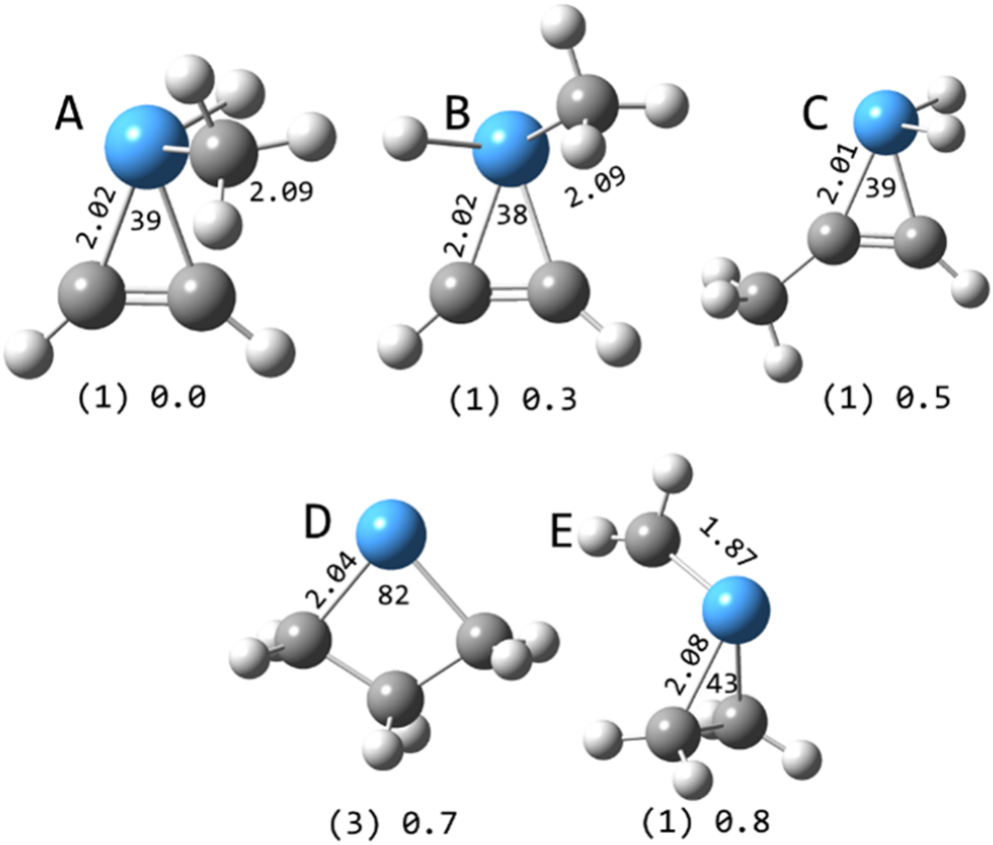
Selected structures of TaC_3_H_6_^+^ calculated at the B3LYP/def2-TZVP level. Multiplicities in parentheses and relative energies in eV are indicated along with Ta–C bond lengths (Å) and C–Ta–C angles (degrees).

**Figure 9:**
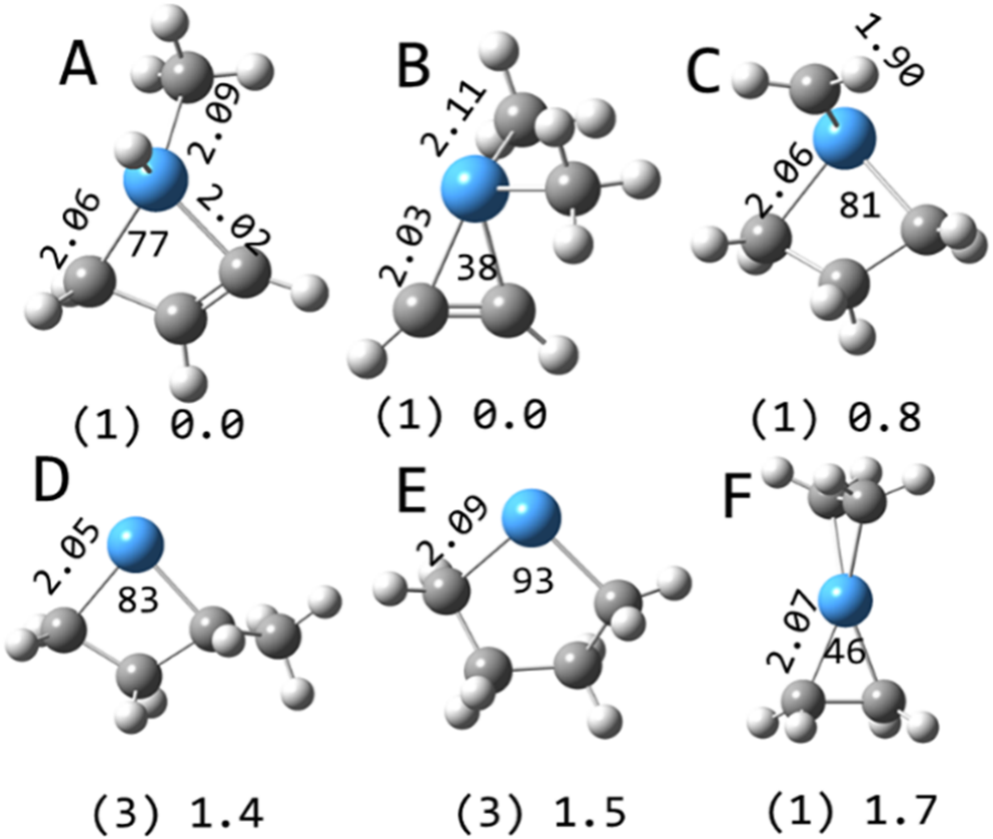
Selected equilibrium structures of TaC_4_H_8_^+^ calculated at the B3LYP/def2-TZVP level. Multiplicities in parentheses and relative energies in eV are indicated along with Ta–C bond lengths (Å) and C–Ta–C angles (degrees).

**Figure 10:**
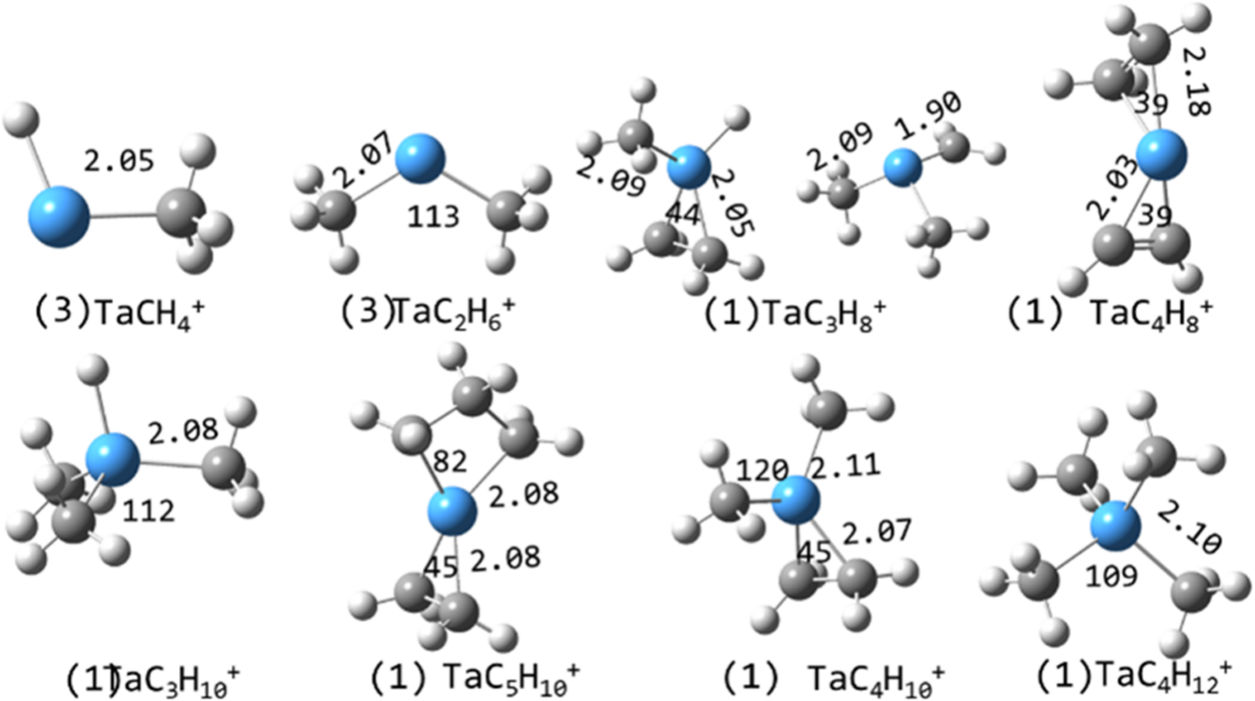
Global minimum energy structures of selected species calculated at the B3LYP/def2-TZVP level. Multiplicities are in parentheses and Ta–C bond lengths (Å) and C–Ta–C angles (degrees) are indicated. The two structures shown for TaC_3_H_8_^+^ are calculated to be of similar energy.

**Figure 11:**
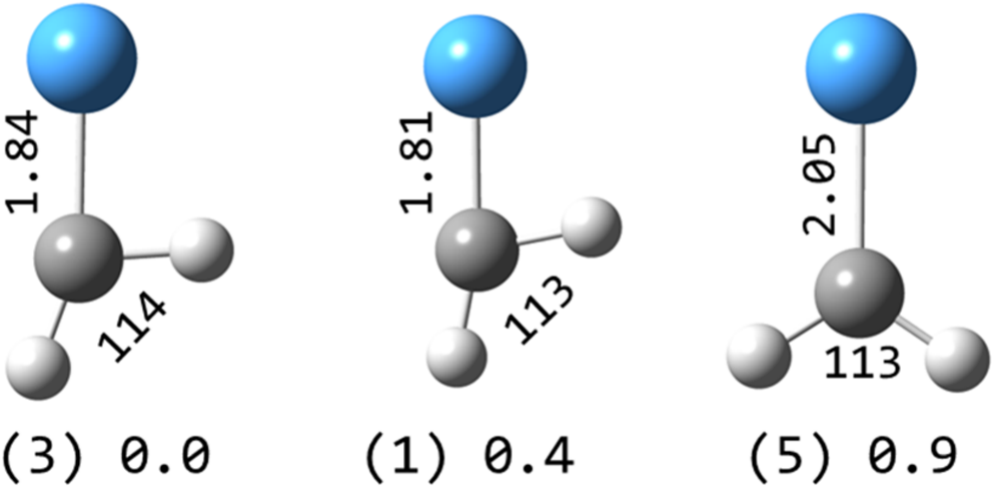
Lowest energy structures of TaCH_2_^+^ calculated at the B3LYP/def2-TZVP level at each indicated multiplicity (in parentheses). Relative energies in eV are indicated. Ta–C bond lengths (Å) and H–C–H angles (degrees) are shown.

The only low-lying TaC_2_H_2_^+^ structure is the tantalapropene cation on the triplet surface (Figure 5), containing Ta–C single bonds and two unpaired valence electrons. It is useful to note that typical Ta–C bond lengths for a single bond are between 2 and 2.1 Å and for a double bond between 1.85 and 1.9 Å. The C–C bond distance (1.35 Å) is increased relative to acetylene (1.20 Å). On the singlet surface, the Ta–C bonds have increased electron density, approaching double bonds, while the C–C bond length is increased (1.43 Å). On the quintet surface, one-electron Ta–C bonds are formed and the C_2_H_2_ moiety is only slightly distorted from acetylene with a 1.25 Å bond length and a 159 °C–C–H angle. The structure of TaC_2_H_2_^+^ resulting from the Ta^+^ + C_2_H_4_ reaction has been investigated spectroscopically,^18^ confirming a tantalapropene structure, but not differentiating between the singlet and triplet structures. Similar metallapropene structures have been noted for a number of transition metal cation systems.^42–44^

The lowest energy calculated structures of TaC_2_H_4_^+^ on the singlet, triplet, and quintet surfaces are shown in Figure 6. The quintet structure is an electrostatically bound species at elevated energy. The lowest energy triplet structure is the tantalapropane cation. On the singlet surface, the lowest two energy structures are dihydride tantalapropene cations, differing slightly in energy. A dihydride structure has also been identified through both calculation and spectroscopy as the lowest energy structure in the Ta_4_CH_2_^+^ system.^45^ The singlet tantalapropane structure is 1.1 eV higher in energy, while the tantalabiscarbene structure is 0.9 eV above the ground state. The tantalapropane and tantalapropene structures all comprise single Ta–C bonds. The tantalapropane structures each have two unbound valence electrons located primarily on the tantalum (paired in the singlet structure and unpaired in the triplet). The tantalapropene and tantalabiscarbene structures have no unbound valence electrons, the latter comprising Ta–C double bonds.

The singlet tantalapropene carbene cation is the lowest energy TaC_3_H_4_^+^ structure (Figure 7), while the singlet and triplet tantalabutene structures are at higher energy. The tantalapropene carbene has no unbound valence electrons, while the tantalabutene structures each have two unbound valence electrons located primarily on the tantalum atom.

TaC_3_H_6_^+^ has a number of low-lying structures, mostly on singlet surfaces. The lowest energy triplet structure is the tantalabutane (Figure 8).

TaC_4_H_8_^+^ is calculated to only have low-lying structures on singlet surfaces, none of which have unbound valence electrons (Figure 9). The tantalabutene methyl hydride and tantalapropene dimethyl structures are calculated to be isoenergetic. The lowest-energy structure found on a triplet surface is the tantalamethylbutane (the tantalapentane (not shown) is slightly higher in energy). The interesting bimetallacycle (tantalaspiropentane, perhaps) structure is highly elevated in energy.

The global minimum structures for several other TaR^+^ species are shown in Figure 10.

## Discussion

### C–H Activation Mechanisms

The Ta^+^ + CH_4_ dehydrogenation reaction has been well-detailed, proceeding by oxidative insertion of the Ta into a C–H bond.^10,15^ Harvey et al. refer to this, i.e., oxidative addition to transition metals, as a “ubiquitous” manner of hydrocarbon activation.^46^ Briefly, oxidative addition involves the breaking of a C–H bond, energetically enabled by the concerted formation of two new covalent bonds with the inserted species (e.g., Ta^+^); one bond is formed with the hydrogen atom and another with the alkyl fragment, increasing the oxidation state of the inserted species. Because the cleaved C–H bond necessarily involved two electrons of opposite spin, the newly formed covalent bonds require two additional electrons of opposite spin. If those electrons are not readily available (i.e., as unbound valence electrons on the inserting species), the mechanism requires either promotion of an electron, or a spin-flip of an electron, i.e., an ISC to a lower spin surface. This is the case for Ta^+^ + CH_4._ The insertion does not occur on the reactant ground state quintet surface because all valence electrons are of common spin, instead proceeding on the triplet surface.

An expectation for facile oxidative addition to a TaR^+^ species can be summed up in the simple heuristic that at least one unbound valence electron of each α and β spin is available. Or stated differently, that the inserting species is both electronically and coordinatively unsaturated. Explaining the observed sequential dehydrogenations initiated by Ta^+^ + CH_4_ through the same mechanism presents a problem: because the inserted species increases in oxidation state, eventually the TaR^+^ species will become saturated and the activation energy of the oxidative insertion will be prohibitive. As Ta^+^ is a quintet ground state, this simplified view would predict no more than two successive processes, first yielding a triplet product and finally a singlet species. Once the TaR^+^ species becomes saturated, the activation energy of the oxidative insertion becomes prohibitive, but three successive dehydrogenations are observed.

One possibility to resolve the issue is a post-transition state ISC back to a higher spin surface, such as is well-known for the prototypical two-state reactivity process FeO^+^ + H_2_.^47,48^ For instance, if R_1,2→2,4_ yields the TaC_2_H_4_^+^ tantalapropane cation (Figure 6C), then both the product and the TaCH_2_^+^ reactant would be unsaturated triplet species in identical oxidation states, with a higher oxidation state on the singlet surface only sampled as an intermediate. The tantalapropane cation could interact with an additional methane molecule similarly, undergoing an ISC to a singlet species to enable the insertion, followed by isomerization and ISC back to a triplet surface and yielding the tantalabutane cation (Figure 8D). Electrons can be pushed in this manner to dehydrogenate any number of methane molecules, yielding ever large tantala-alkane cations. However, inspection of the calculated energetics of the TaR^+^ species shows that for TaC_2_H_4_^+^ and larger TaR^+^, the ground state isomers are singlet species. If the reactant species are the triplet cations that are predicted to undergo facile oxidative insertion, then they are in metastable geometries.

An alternate possibility (shown below to more likely) is that the saturated singlet TaR^+^ species can activate and dehydrogenate methane via a distinct mechanism. Transition metals may less commonly activate hydrocarbons via σ-bond metathesis (σ-BM).^49^ Similar to oxidative insertion, two new covalent bonds are formed, but in σ-BM two, not one, covalent bonds are also broken, and the resulting species have not changed oxidation state. The prototypical form of σ-BM is
9

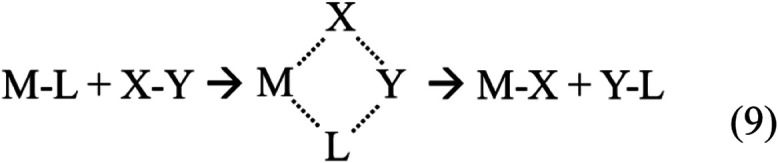

That is, a metal–ligand bond and an incident molecular bond are cleaved and the constituents exchanged through a four-center transition state. Regardless of energetics, the mechanism cannot easily explain methane dehydrogenation from nonmetallacycle TaR^+^ species, as only a single hydrogen atom could be transferred. For example, TaCH_3_^+^ + CH_4_ undergoing σ-BM would result only in a hydrogen exchange, yielding the reactant species. However, for metallacycle TaR^+^ species, if one of the Ta–C bonds is cleaved, the R group can remain tethered to the tantalum atom by the other Ta–C bond. The resulting (CH_3_)­TaRH ^+^ species includes an already activated methane and retains the possibility of undergoing dehydrogenation via subsequent isomerizations.

Below, the various TaR^+^ reactions are explored within this framework, integrating the kinetics measurements with the density functional calculations. We apply the simple heuristic that the oxidative insertion reaction should be facile if the reactant ion has at least one unbound valence electron of each spin available to all the TaR^+^ species. In Figure 3, these categories are color-coded green for a predicted small activation energy or red for a predicted large activation energy, respectively. By inspection, the heuristic fairs well, with large dehydrogenation rate constants observed by “green” species and small or zero rate constants observed for “red” species, with the exceptions of TaC_2_H_4_^+^ and TaC_3_H_6_^+^. This suggests that those processes occur through a mechanism other than oxidative insertion of the Ta atom into a C–H bond, e.g., σ-BM.

### TaCH_2_^+^+ CH_4_ (R_1,2→2,4_): Dehydrogenation via Carbon–Carbon Bond Formation

The requirement of at least one unbound valence electron of each α and β spin for facile oxidative insertion of Ta into a C–H bond is further illustrated using quantum chemical calculations of the insertion transition state in the TaCH_2_^+^ + CH_4_ reaction.

The lowest energy structures of TaCH_2_^+^ for each spin state are shown in Figure 11. These DFT results are similar to those reported previously, along with spectroscopic evidence of the agostic interaction.^16^ Both the triplet and singlet structures form Ta–C double bonds, characterized by a bond length between 1.8 and 1.9 Å. The quintet structure forms a three-electron (1.5) Ta–C bond with a longer bond length of between 2.0 and 2.1 Å. The Ta–C bond strength decreases by 0.9 eV from the triplet to the quintet. The asymmetry in the triplet and singlet structures is due to a weak agostic interaction between the hydrogen atom and unoccupied d-orbitals of the tantalum.

The calculated reaction coordinates for oxidative insertion of the tantalum atom in a C–H bond for TaCH_2_^+^ + CH_4_ are compared in Figure 12 on a triplet surface and on a singlet surface. On the singlet surface (Figure 12, dashed black curve), the reaction must overcome an activation energy of just 0.2 eV above the TaCH_2_^+^(CH_4_) entrance complex. The equivalent reaction coordinate on the triplet surface (Figure 12, solid black curve) shows a larger activation energy of about 1.2 eV. The large difference has a clear origin. The unbound valence electrons in singlet TaCH_2_^+^ are of opposite spin and are available to form both Ta–C and Ta–H bonds with the homolytically cleaved CH_3_–H fragments. The unbound valence electrons in triplet TaCH_2_^+^ are of common spin, and only one of these electrons can form a bond with the CH_3_–H fragments, the other necessarily being of the same spin as the free electron on the corresponding fragment. In order to complete the oxidative insertion, an electron of opposite spin must be promoted.

**Figure 12:**
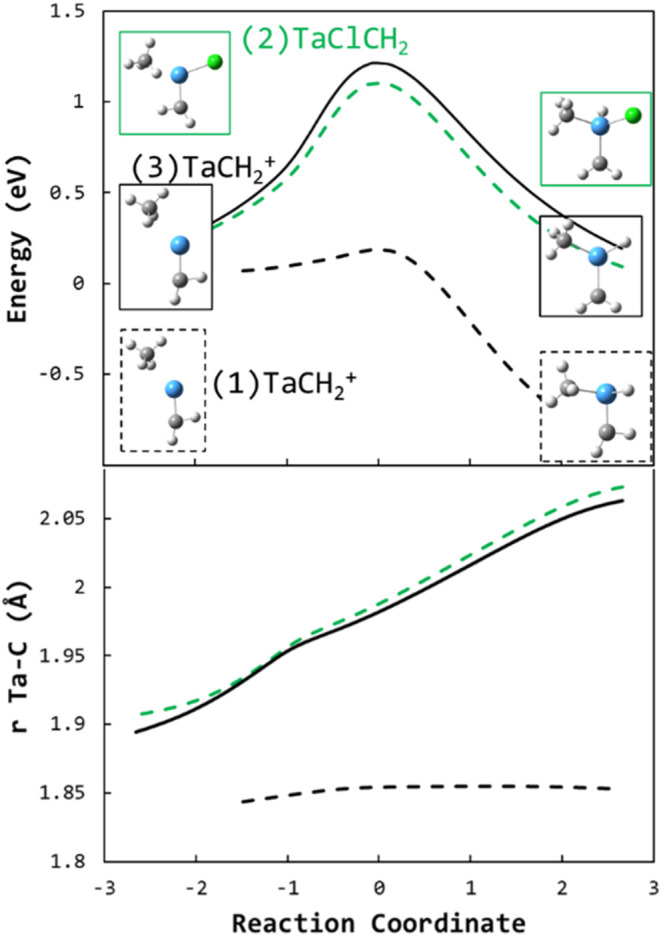
Energies (top) and Ta–C bond lengths (bottom) along the calculated reaction coordinates for oxidative insertion of Ta into a C–H bond for triplet TaCH_2_^+^ + CH_4_ (solid black curve), singlet TaCH_2_^+^ + CH_4_ (dashed black curve), and doublet TaClCH_2_^+^ + CH_4_ (dashed green curve). Energies are relative to the TaR^+^(CH_4_) entrance complex for each reaction. Structures at the minima are shown.

Comparing the Ta–C bond lengths of the singlet and triplet reactions (Figure 12, bottom panel), the bond length on the singlet surface remains constant throughout the reaction, while that on the triplet surface increases from 1.84 Å (a double bond) to 2.07 Å (a 1.5 bond). That is, the additional electron required for the insertion on the triplet surface is removed from the Ta–C bond (a conclusion also supported by Natural Bond Orbital analysis). The significant energetic cost is reflected in the increased activation energy (∼1 eV), corresponding closely to the difference in bond strength between the Ta–C double and 1.5 bonds (∼0.9 eV).

As an additional illustration, the equivalent reaction coordinate of TaClCH_2_^+^ + CH_4_ oxidative insertion is shown in Figure 12. The Cl bond acts as a spectator, only affecting the reaction by sequestering a single valence electron from the tantalum atom. The low-spin doublet TaClCH_2_^+^ has a single unbound valence electron and the calculated reaction coordinate (Figure 12, green dashed curves) energy and Ta–C bond length are nearly identical to those of triplet TaCH_2_^+^, which has two unbound valence electrons. The additional unbound valence electron in triplet TaCH_2_^+^ provides no benefit despite the oxidative insertion requiring a second electron; that electron must be of the opposite spin and be promoted from the Ta–C bond the same as in the TaClCH_2_^+^ reaction.

A calculated reaction profile for R_1,2→2,4_ is shown in Figure 13. TaCH_2_^+^ coordinates with methane to form an electrostatically bound complex on a triplet surface (stationary point ^3^ES in Figure 13). The oxidative insertion of Ta into a C–H bond is energetically restricted on the triplet surface, and is instead accessed on the singlet surface via ISC, forming a Ta–C single bond in addition to the pre-existing Ta–C double bond (^1^OA, Figure 13).

**Figure 13:**
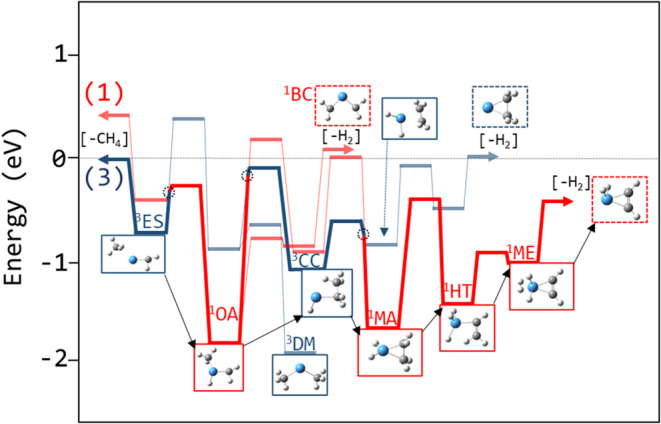
Reaction coordinate of TaCH_2_^+^ + CH_4_ → TaC_2_H_4_^+^ + H_2_ calculated at the B3LYP/def2-TZVP level along the triplet (blue) and singlet (red) surfaces. The expected pathway is bolded and the selected stationary point structures are indicated. Dotted circles are estimated locations of the crossing seams.

While a second tantalum carbene double bond can be formed (i.e., Ta­(CH_2_)_2_^+^), the calculated bond strength is weaker than the initial Ta^+^-CH_2_ bond, dropping below the 4.81 eV threshold for exothermic elimination of H_2_. Even though the reaction can navigate the oxidative insertion, it is energetically prohibited from completing the reductive elimination of H_2_ directly (^1^BC, Figure 13). Isomerization through carbon–carbon bond formation can provide a submerged pathway to H_2_ elimination, but this is not possible on the singlet surface alone.

A methyl migration replaces the Ta–CH_3_ bond in ^1^OA with a C–C bond (^3^CC, Figure 13). The C–C bond is slightly stronger and, on the triplet surface, this results in a small energetic benefit. However, on the singlet surface, the Ta–CH_2_ double bond, which was retained during the oxidative insertion, must transition to a single bond as the carbon transitions from sp^2^ to sp^3^ hybridization; note the increase in the Ta–C bond length along the reaction coordinate (Figure 14, bottom). The energetic cost avoided during the oxidative insertion on the singlet surface is paid now (Figure 14, top). A second ISC back to a triplet surface is needed to access the lower energy transition to form ^3^CC. While the subsequent elimination of H_2_ is energetically accessible on either spin surface, after this point, the calculated reaction coordinates on the singlet and triplet surfaces differ qualitatively.

**Figure 14:**
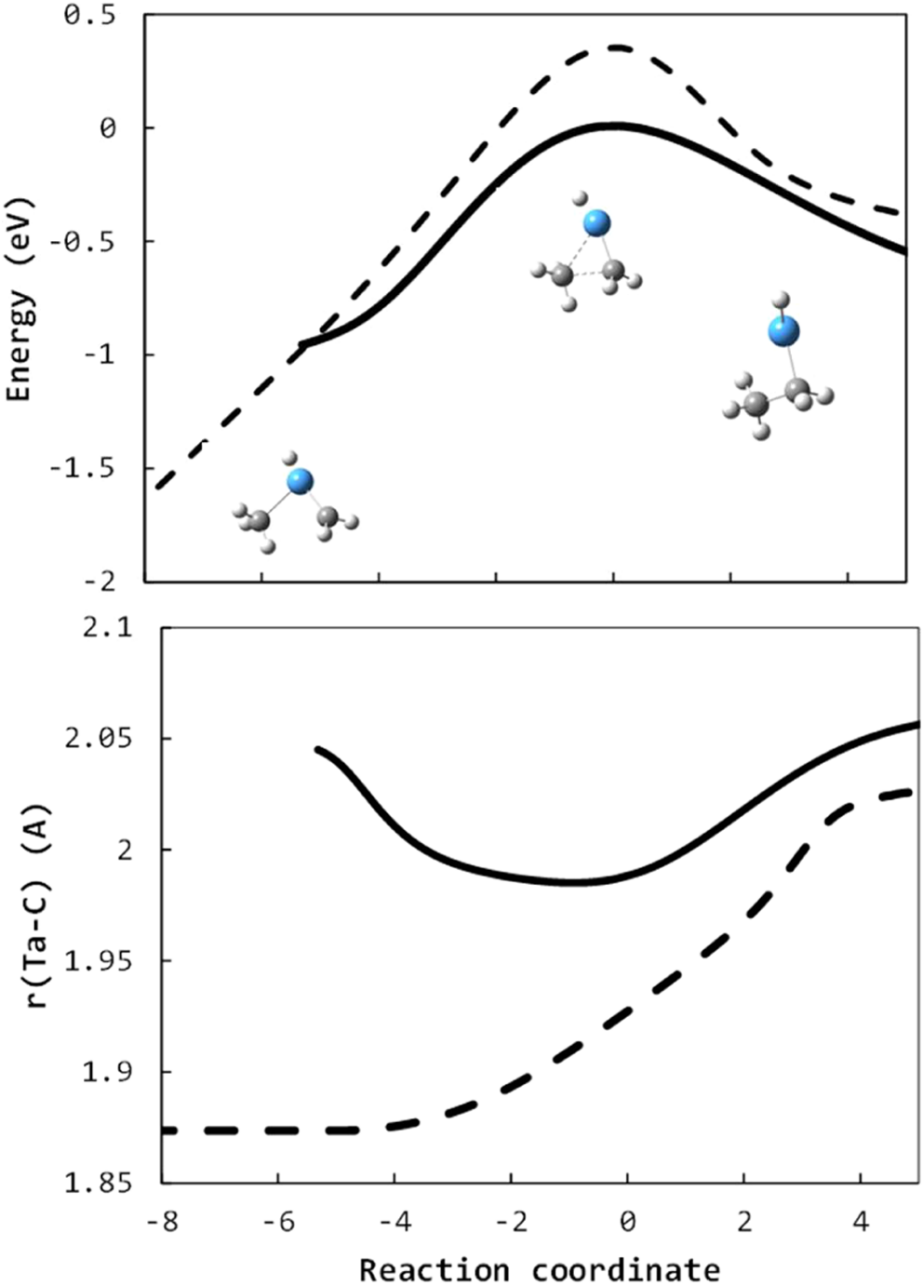
Energy (top) relative to separated reactants and Ta–C bond length (bottom) along the reaction coordinates of singlet (dashed) and triplet (solid) TaCH_2_^+^ + CH_4_ during the carbon–carbon bond formation step.

As a hydrogen migrates from the methyl to the tantalum atom, ISC back to the singlet surface allows for a tantalapropane metallacycle (^1^MA) to be formed. Subsequent isomerization to a tantalapropene metallacycle (^1^ME) is followed by dehydrogenation to the ground state H_2_TaC_2_H_2_^+^ tantalapropene dihydride cation (Figure 6A) isomer. If ISC does not occur during the hydrogen migration from ^3^CC, the metallacycle bond cannot be formed. Instead, the hydrogen shift to the Ta is concerted with breaking the remaining Ta–C covalent bond, yielding TaH_2_^+^(C_2_H_4_), that is an ethylene electrostatically bound to TaH_2_^+^. Subsequent dehydrogenation to a TaC_2_H_4_^+^ tantalapropane cation (Figure 6C) calculated to be nearly isoenergetic with the TaCH_2_^+^ + CH_4_ reactants. Notably, the dehydrogenation of TaCH_2_^+^ + CH_4_ requires multiple ISC, all of which must occur with high efficiency in order for the reaction to proceed with the observed high efficiency.

As discussed above, the thermal dissociation of TaC_2_H_4_^+^ to TaC_2_H_2_^+^ provides information on the TaC_2_H_2_^+^–H_2_ bond energy of the species produced in R_1,2→2,4_. The derived bond energy corresponds much more closely to the ground state H_2_TaC_2_H_2_^+^ tantalapropene dihydride cation isomer (Figure 5A) than to the higher lying TaC_2_H_4_^+^ tantalapropane cation isomer (Figure 5C), which is expected to rapidly dissociate under the present experimental conditions. This interpretation largely agrees with that presented previously by Simon et al.^12^ and is distinct from that presented more recently by Eckhard et al.^20^

Interestingly, the most facile isomerization from the ^1^OA complex may be hydrogen transfer from the methyl group to the carbene group, enabled by an ISC back to the triplet surface. The resulting tantalum dimethyl cation (Figure 13, ^3^DM) is the global minimum found for the TaC_2_H_6_^+^ system. While this structure could be stabilized through collisions with the buffer gas, no direct exothermic pathway to bimolecular products is found. Instead, only isomerization back to ^1^OA can occur, followed by several steps to finally achieve dehydrogenation.

### TaC_2_H_4_^+^ + CH_4_ (R_2,4→3,6_): Dehydrogenation without Carbon–Carbon Bond Formation via Ring-Opening σ-Bond Metathesis

TaC_2_H_4_^+^ rapidly dehydrogenates another methane molecule (R_2,4→3,6_ occurs at over 50% of the Langevin capture rate). However, the ground state tantalapropene dihydride cation (Figure 6A) has no unbound valence electrons, and oxidative insertion of Ta is expected to have a large activation energy. If the ground state isomer is formed from R_1,2→2,4_, the sequential methane dehydrogenation must occur through an alternative mechanism, such as σ-BM.

Density functional calculations (Figure 15, yellow pathway) confirm that the transition state for oxidative insertion of tantalum into a C–H bond is energetically inaccessible, lying about 0.5 eV above the H_2_TaC_2_H_2_^+^ + CH_4_ reactants. Similarly, isomerization to the tantalapropane structure (Figure 6E), which has only a small activation energy to a reactive inserted structure (^1^MA, Figure 15, blue pathway) cannot occur at thermal energies whether the hydrogen transfers occur in a concerted (Figure 15, red pathway) or sequential (Figure 15, purple pathway). Instead, a low-energy pathway (Figure 15, green pathway) occurring on a singlet surface without ISC is found involving σ-BM insertion of the entire tantalapropene moiety in a methane C–H bond.

**Figure 15:**
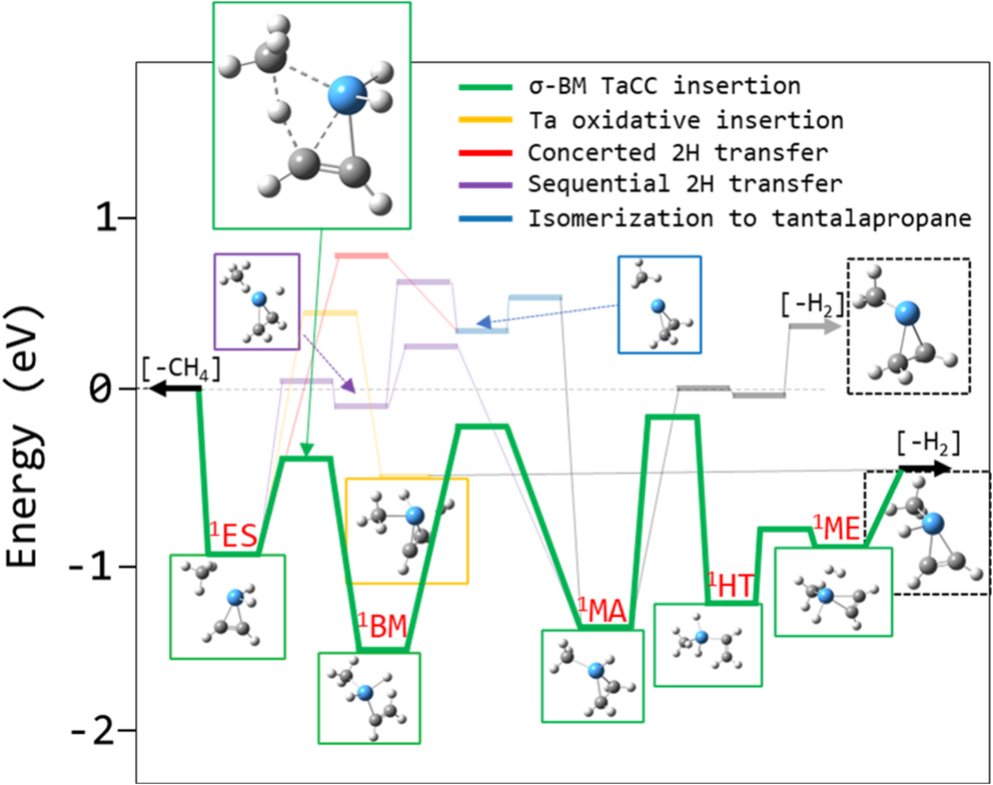
Reaction coordinate of R_2,4→3,6_ TaC_2_H_4_^+^ + CH_4_ → TaC_3_H_6_^+^ + H_2_ calculated at the B3LYP/def2-TZVP level. All structures are on a singlet surface. Predicted dominant pathway is in bold; dispreferred pathways are ghosted.

The insertion initiates a hydrogen transfer from the electrostatically bound methane (^1^ES) to a carbon in the metallacycle. At the characteristic 4-center transition state (larger inset of Figure 15), the methyl has formed a one-electron bond to the tantalum, subsequent to which the Ta–C bond with the abstracting carbon in the metallacycle breaks, freeing an electron for the tantalum to complete a single bond with the methyl while the intramolecular hydrogen abstraction is completed. The resulting intermediate (^1^BM) is not a metallacycle, instead a methyl vinyl tantalum dihydride cation. Although chemically similar in ways to oxidative insertion, the oxidation state of the Ta atom has not changed, as the two newly formed covalent bonds are only possible due to the concerted breaking of the two pre-existing covalent bonds. The typical picture of σ-bond metathesis involves an exchange resulting in separated products, but here, because of the metallacycle reactant structure, while one Ta–C bond was broken, the carbon chain remains tethered to the tantalum atom. No separated products are formed at this step, and the insertion results in an intermediate species with all atoms available to undergo further chemistry. An analogous process involving a noncyclic moiety, e.g., TaCH_3_^+^ + CH_4_, would result in separated products, in the example given, a symmetric hydrogen exchange reaction; although not explored here, this is a possible mechanism for hydrogen isotopic scrambling observed for some TaR^+^ + CD_4_ in other experiments.^20^

The ^1^OA structure from the R_1,2→2,4_ reaction proceeded via methyl migration to form a carbon–carbon bond, followed by a hydrogen transfer from a carbon to the tantalum to form the ^1^MA tantalapropane dihydride structure (Figure 13). Along the R_2,4→3,6_ coordinate, the methyl migration requires overcoming a transition state calculated at 0.7 eV above the separated reactants. Instead, a single hydrogen transfer from the tantalum to a carbon yields the analogous ^1^MA tantalapropane methyl hydride structure directly. R_2,4→3,6_ can then follow an analogous path to R_1,2→2,4_ to isomerize to a tantalpropene species (^1^ME) followed by dehydrogenation to yield the ground state TaC_3_H_6_^+^ tantalapropene methyl hydride cation (Figure 15, Figure 8A).

The dehydrogenation R_2,4→3,6_ differs from R_1,2→2,4_ in four qualitative aspects. First, the insertion into a C–H bond occurs across the TaCC tantalapropene moiety, not only across the Ta atom. Second, the insertion is via a ring-opening σ-bond metathesis, not a redox process. Third, while R_2,4→3,6_ requires three separate ISC, R_2,4→3,6_ takes place entirely along a singlet surface and no ISC is required. Fourth, the subsequent dehydrogenation is not (indeed at thermal energies cannot be) enabled by additional carbon–carbon bond formation. The reaction is highly efficient and therefore not significantly affected by the expected entropic constraint of a four-center transition state.

### TaC_3_H_6_^+^ + CH_4_ (R_3,6→4,8_): Further Dehydrogenation via Ring-Opening σ-Bond Metathesis

Similar to TaC_2_H_4_^+^, TaC_3_H_6_^+^ has a ground state singlet isomer containing a tantalapropene moiety that is predicted to have a large activation energy to the insertion of a Ta atom. Despite this, the R_3,6→4,8_ reaction proceeds very quickly, near the Langevin capture limit at 300 K. Also similar to TaC_2_H_4_^+^, TaC_3_H_6_^+^ may instead insert the entire tantalapropene moiety into a C–H bond via σ-BM, with the calculated transition state slightly submerged relative to separated reactants. Calculated energies for the lowest energy product TaC_4_H_8_^+^ isomers, tantalabutene methyl hydride cation (implying additional carbon–carbon bond formation, Figure 9A) and tantalapropene dimethyl cation (implying no carbon–carbon bond formation has occurred 9B) are similar. Methyl migration within the intermediate formed after insertion (^1^BM, Figure 16) would yield the methyl tantalapropane methyl hydride cation and subsequently the tantalabutene methyl hydride product (i.e., additional C–C bond formation; purple pathway, Figure 16), while hydrogen transfer would yield the tantalapropane dimethyl cation (^1^MA) and subsequently the tantalapropene dimethyl product (i.e., no C–C bond formation; green pathway, Figure 16). While TS along both pathways are calculated at higher energy than separated reactants, the observed efficiency of R_3,6→4,8_ suggests that one of these transition states is, in fact, submerged, and the hydrogen transfer at 0.1 eV above separated reactants is much preferred to the methyl migration at 0.9 eV. The experiment does not provide direct evidence of the product structure, but the observed kinetics, combined with the DFT calculations, suggest that additional carbon–carbon bond formation does not occur.

**Figure 16:**
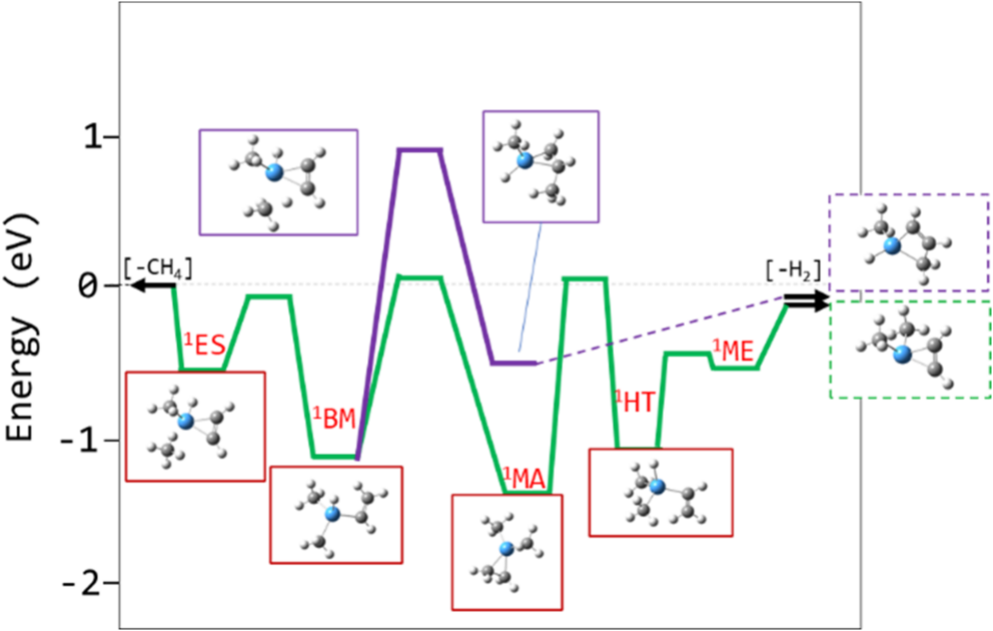
Calculated reaction coordinate for R_3,6→4,8_ at the B3LYP/def2-TZVP level. Predicted green pathway leads to the tantalapropene dimethyl cation product, while the dispreferred purple isomerization could lead to the tantalabutene methyl hydride cation product. Both dehydrogenation products shown are calculated to be of similar energy.

### TaC_4_H_8_^+^+ CH_4_ (R_4,8→5,12_)

The main sequence of methane dehydrogenation reactions terminates at R_4,8→5,10_, which does not occur within the sensitivity of the experiment. Instead, a rapid association reaction R_4,8→5,12_ is observed, followed by thermal dissociation of TaC_5_H_12_^+^ back to TaC_4_H_8_^+^. Indeed, no exothermic dehydrogenation product is found from the DFT calculations, with the lowest energy TaC_5_H_10_^+^ isomer identified, bitantala[3.2]­pentane (i.e., a bimetallacycle comprising tantalabutane and tantalapropane moieties, Figure 10), implying an endothermicity of 0.9 eV for R_4,8→5,10_ (the intuitively appealing tantalahexane cation is 0.9 eV again higher in energy). At the same DFT level, the transition state for insertion of the tantalapropene moiety (i.e., a σ-bond metathesis mechanism analogous to that for R_2,4→3,6_ and R_3,6→4,8_) is calculated slightly (0.1 eV) below the energy of separated reactants and may occur.

Some aspects of methane activation, or lack thereof, are elucidated by inspection of reactions outside of the main sequence terminating in TaC_4_H_8_^+^, and these are discussed briefly below.

### TaC_2_H_2_^+^ + CH_4_ (R_2,2→3,4_)

TaC_2_H_2_^+^ has not been reported in previous flow tube studies, likely because the production at room temperature is very small, near the noise level of our experiment. Injecting either Ta^+^ or TaCH_2_^+^ into the flow tube yields increasingly large abundances of TaC_2_H_2_^+^ with increasing temperature, which we assign to thermal dissociation of TaC_2_H_4_^+^ product. Injecting TaC_2_H_2_^+^ directly into the flow tube shows an efficient dehydrogenation process to form TaC_3_H_4_^+^. The only low-lying TaC_2_H_2_^+^ structure is tantalapropene cation (Figure 5A) in a triplet state, which has two unpaired valence electrons available of common spin. Similar to the TaCH_2_^+^ reaction, oxidative insertion could be enabled by a facile ISC to the singlet surface after complexation with CH_4_. Alternatively, the reaction could occur without ISC via σ-BM. The present data do not distinguish between these possibilities. At the B3LYP/def2-TZVP level, no isomer of TaC_3_H_4_^+^ can be produced exothermically from TaC_2_H_2_^+^ + CH_4_. As the observed kinetics indicate an exothermic R_2,2→3,4_ reaction is occurring, we assume the calculated energetics are in error and the only low-lying isomer, tantalapropene carbene cation Ta­(CH_2_)­C_2_H_2_^+^, is formed exothermically.

### TaC_3_H_4_^+^+ CH_4_ (R_3,4→4,6_)

TaC_3_H_4_^+^ does not efficiently dehydrogenate methane (R_3,4→4,6_), despite the reaction being similarly slightly exothermic (∼0.1 eV) to other dehydrogenation reactions measured (R_0,0→1,2_, R_1,2→2,4_, R_2,2→3,4_, R_2,4→3,6_, R_3,6→4,8_). R_3,4→4,6_ proceeds with a small rate constant of about 10^–12^ cm^3^ s^–1^ at 300 K, increasing with an apparent activation energy of about 0.2 eV (Figure 17). This behavior is consistent with the lowest energy isomer of TaC_3_H_4_^+^ singlet tantalapropene carbene cation (Figure 7A), with no unbound valence electrons available for oxidative insertion of the tantalum atom to the methane suggesting a very large activation energy, as opposed to the higher energy tantalabutane structure, which should undergo facile oxidative insertion of the tantalum atom. The TaC_3_H_4_^+^ singlet tantalapropene carbene cation can undergo insertion of the entire tantalapropene moiety into a C–H bond via σ-BM, similar to the TaC_2_H_4_^+^ reaction described above. The transition state for this insertion is calculated at 0.2 eV above the separated reactants, matching the observed activation energy (although the exact agreement is fortuitous). The inefficient dehydrogenation of methane by TaC_3_H_4_^+^ is not qualitatively distinct from the rapid dehydrogenation enabled by TaC_2_H_4_^+^ or TaC_3_H_6_^+^, occurring also by σ-BM and without C–C bond formation, but is inhibited by a transition state only somewhat higher in energy.

**Figure 17:**
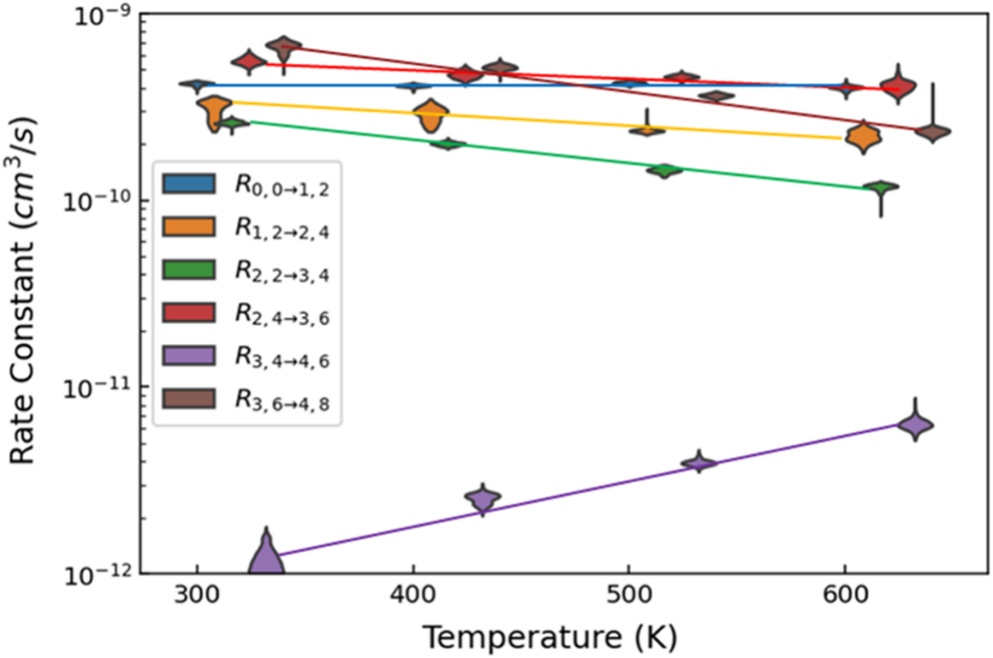
Rate constants of the indicated dehydrogenation reactions (those with kinetics well-defined by the data here) as a function of temperature. Lines added to guide the eye.

### TaC_2_H_6_^+^+ CH_4_ (R_2,6→3,10_)

The total rate constant for TaC_2_H_6_^+^ + CH_4_ is near the Langevin capture value at all temperatures. At lower temperatures, the association reaction R_2,6→3,10_ dominates, but the rate constant decrease sharply as T^–2.5^, typical for an association.^39^ The rate constant of the dehydrogenation reaction R_2,6→3,8_ increases rather sharply with temperature, a notably different behavior than the other efficient dehydrogenation processes observed. These kinetics are well explained by the framework developed from the comparison of quantum chemical calculations to the measured kinetics of the reactions discussed above.

The ground state tantalum dimethyl cation Ta­(CH_3_)_2_^+^ has two unpaired valence electrons available on the tantalum, and assuming ISC to a singlet surface, TaC_2_H_6_^+^ + CH_4_ may proceed by facile oxidative insertion of the tantalum atom into a C–H bond (analogous to R_0,0→1,2_ and R_1,2→2,4_). The resulting intermediate tantalum trimethyl hydride cation TaH­(CH_3_)_3_^+^ (Figure 10) is bound by about 2 eV relative to reactants. This is a deeper well than in other TaR^+^ systems considered here, enabling a more rapid stabilization of the intermediate. The dehydrogenation reaction R_2,6→3,8_ is calculated to be slightly exothermic and competes from the TaH­(CH_3_)_3_^+^ intermediate. It is not immediately clear if carbon–carbon bond formation occurs in the reaction, as either the tantalapropane methyl hydride cation (Figure 10, TaC_3_H_8_^+^ left) or tantalum dimethyl carbene cation (Figure 10, TaC_3_H_8_^+^ right) products could reasonably be formed, although as argued below likely no C–C bond formation occurs. In either case, the observed positive temperature dependence of R_2,6→3,8_ arises from the decreasing competition of the association reaction, not from a required activation energy.

### TaC_3_H_8_^+^+ CH_4_ (R_3,8→4,12_)

Although the dehydrogenation reaction R_3,8→4,10_ is calculated to be about 0.3 eV exothermic, it is observed to proceed with only a very small rate constant if at all. Instead, the association reaction R_3,8→4,12_ dominates, occurring near the Langevin capture limit. The only low-lying isomer of TaC_4_H_12_^+^ identified is the tantalum tetramethyl cation (Figure 10) and must be the association product. For both possible TaC_3_H_8_^+^ isomers discussed as possible products of R_2,6→3,8_, oxidative insertion of the tantalum atom should be inaccessible. From the tantalapropane methyl hydride cation, σ-BM insertion of the tantalapropane moiety into the C–H bond of an incident methane appears possible, but further isomerization to the tantalum tetramethyl cation would require carbon–carbon bond breaking along with additional rearrangement. On the other hand, an association of the TaC_3_H_8_^+^ tantalum dimethyl carbene cation to the tantalum tetramethyl cation requires only a hydrogen transfer from the incident methane to the carbene. The dominance of the association reaction here supports that R_2,6→3,8_ produced tantalum dimethyl carbene cation and no carbon–carbon bond formation occurred.

### TaC_3_H_10_^+^+ CH_4_

Similar to TaC_3_H_8_^+^ above, the dehydrogenation reaction R_3,10→4,12_ is calculated to be exothermic but is not observed to occur, indicating a kinetic constraint. The competing association reaction R_3,10→4,14_ is also not observed to any significant extent and TaC_3_H_10_^+^ is mostly unreactive under the present conditions. The calculated lowest energy isomer, the tantalum trimethyl hydride cation (Figure 10), has no unbound valence electrons, making oxidative addition prohibitive, and does not contain a metallacycle moiety, preventing a ring-opening σ-bond metathesis. None of the above insertion mechanisms appear plausible, commensurate with the observed lack of reactivity.

## Conclusions

The sequential activation of methane molecules by a Ta^+^ atomic cation is a remarkable chemical feat. Each dehydrogenation process must overcome a series of obstacles: the reactions are only slightly exothermic; the adiabatic pathway may require one or more intersystem crossings, intramolecular carbon–carbon bond formation may have to occur; and each successive activation risks saturating the tantalum atom either coordinatively or electronically, precluding further reactivity. Yet the reactions do occur, and at a substantial fraction of the Langevin capture rate. Here, measurement of the rate constants and temperature dependences of a large chemical network initiated by Ta^+^ + CH_4_ are combined with a novel analysis technique and extensive density functional calculations to understand the mechanisms of activation.

The established mechanism for Ta^+^ + CH_4_ involving oxidative insertion of the Ta into a C–H will only have a low activation energy for TaR^+^ species that retain two unpaired valence electrons of opposite spin on the tantalum atom. Assuming facile ISC between triplet and singlet surfaces, this condition is met by any otherwise unligated tantalaalkane metallacycle cation. Most intuitively, the sequential reactions could proceed by oxidative insertion followed by isomerization to larger and larger metallacycles (i.e., tantalapropane cation and then tantalabutane cation) with the sequence terminating due to electronic or coordinative saturation of the tantalum (i.e., formation of a species other than tantalapentane cation) or energetic concerns. The evidence presented here is that this intuitive sequence does not occur. The TaCH_2_^+^ + CH_4_ dehydrogenation does activate methane by oxidative insertion and the dehydrogenation is energetically enabled by carbon–carbon bond formation, but the product is the tantalapropene dihydride cation, not the tantalapropane cation.

The barrier for the tantalapropene dihydride cation to activate another methane via oxidative addition is prohibitive. Instead, activation occurs via a ring-opening σ-bond metathesis; i.e., two covalent bonds are broken (CH_3_–H and a Ta–C bond) concerted with forming two new bonds (Ta–CH_3_ and C–H) such that the tantalum atom does not change oxidation state. Unlike a typical σ-bond metathesis yielding separated products, the remaining Ta–C bond acts as a tether and an activated intermediate is formed with all atoms available for further chemistry. Subsequent isomerizations result in dehydrogenation. An analogous mechanism explains the TaC_3_H_6_^+^ + CH_4_ dehydrogenation, while the TaC_4_H_8_^+^ + CH_4_ dehydrogenation is endothermic and does not occur. In no case is a metallacycle moiety larger than tantalapropene formed.

While the dehydrogenation mechanism shifts to σ-bond metathesis once oxidative insertion is no longer viable, this is more of a happy coincidence than a dispreferred mechanism being observed only once a preferred mechanism is blocked. Just as the larger TaR^+^ species cannot undergo oxidative insertion, other TaR^+^ species lacking a metallacycle moiety cannot dehydrogenate via σ-bond metathesis. Note that the efficiency of σ-bond metathesis-enabled processes are in fact larger than those enabled by oxidative insertion. It is possible that the intersystem crossings required for oxidative insertion, while facile, are not of unit efficiency, placing an additional constraint on the reaction rate constants that is not present for σ-bond metathesis.

Several other bare 5d metal cations (W^+^, Os^+^, Ir^+^, Pt^+^) dehydrogenate one or more methane molecules. Each series of reactions must overcome obstacles analogous to those discussed here for Ta^+^. However, while each system has similar weapons available: oxidative insertion, σ-bond metathesis, carbon–carbon bond formation, and intersystem crossings, how those are deployed varies. Careful investigation of the sequential Pt^+^ ^50^ and Ir^+^ ^51^ reactions with methane indeed shows general similarity to the Ta^+^, but differs in important respects, including product structures. Finally, it is possible that ligated species, e.g., metallapropene cations, may activate methane via σ-bond metathesis even in cases where the bare metal cation (e.g., Hf^+^, Re^+^) is unreactive.

## Supplementary Material



## Notes

I. Additional data and analysis; II. Analysis of Simon et al. data; III. Ta^+^ + CH_4_ → TaCH_2_^+^ + H_2_ exothermicity from equilibrium measurements; IV. Calculated thermal dissociation rate constants; V. Density functional calculation summary (PDF)
