# Supplementary material for: Mechanistic Foundations of the Sequential Activation of Methane by Ta+: Oxidative Addition, Ring-Opening σ‑Bond Metathesis, and C–C Bond Formation
Source: J Phys Chem A. 2025 May 6;129(19):4217–33. doi: 10.1021/acs.jpca.5c01569 (PMC12980830; doi:10.1021/acs.jpca.5c01569)
Supplement: Supplementary file 1 [file jp5c01569_si_001.pdf]

Supplemental Information for:

Mechanistic foundations of the sequential activation of methane by Ta^+: Oxidative addition, ring-opening σ-bond metathesis, and C-C bond formation

Tucker W. R. Lewis,[1] Albert A. Viggiano,[1] Brendan C. Sweeny,[1] Jennifer Meyer,[2] Shaun G. Ard,[1,*] and Nicholas S. Shuman[1,*]

[1]*Air Force Research Laboratory, Space Vehicles Directorate, Kirtland AFB NM, USA 87117*

[2]*RPTU Kaiserslautern-Landau, Fachbereich Chemie und Forschungszentrum OPTIMAS, Erwin-Schrödinger Str. 52, 67663 Kaiserslautern, Germany*

*corresponding authors: rvborgmailbox@us.af.mil

I. *Additional data and analysis*
II. *Analysis of Simon et al. data*
III. *$Ta^+ + CH_4 \rightarrow TaCH_2^+ + H_2$ exothermicity from equilibrium measurements*
IV. *Calculated thermal dissociation rate constants*
V. *Density functional calculation summary*

I. *Additional data and analysis*

Table S1. Chemical reaction network initiated by $Ta^+ + CH_4$

Reactant cation	+ CH_4 Products	ID
Ta^+	$TaCH_2^+ + H_2$	$R_{0,0\rightarrow1,2}$
	$TaCH_4^+$	$R_{0,0\rightarrow1,4}$
$TaCH_2^+$	$TaC_2H_4^+ + H_2$	$R_{1,2\rightarrow2,4}$
	$TaC_2H_6^+$	$R_{1,2\rightarrow2,6}$
$TaCH_4^+$	$TaC_2H_6^+ + H_2$	$R_{1,4\rightarrow2,6}$
	$TaC_2H_8^+$	$R_{1,4\rightarrow2,8}$
$TaC_2H_2^+$	$TaC_3H_4^+ + H_2$	$R_{2,2\rightarrow3,4}$
	$TaC_3H_6^+$	$R_{2,2\rightarrow3,6}$
$TaC_2H_4^+$	$TaC_3H_6^+ + H_2$	$R_{2,4\rightarrow3,6}$
	$TaC_3H_8^+$	$R_{2,4\rightarrow3,8}$
$TaC_2H_6^+$	$TaC_3H_4^+ + H_2$	$R_{2,2\rightarrow3,4}$
	$TaC_3H_6^+$	$R_{2,2\rightarrow3,6}$
$TaC_2H_8^+$	$TaC_3H_6^+ + H_2$	$R_{2,4\rightarrow3,6}$
	$TaC_3H_8^+$	$R_{2,4\rightarrow3,8}$

$TaC_3H_4^+$	$TaC_4H_6^+ + H_2$	$R_{3,4\rightarrow4,6}$
	$TaC_4H_8^+$	$R_{3,4\rightarrow4,8}$
$TaC_3H_6^+$	$TaC_4H_8^+ + H_2$	$R_{3,6\rightarrow4,8}$
	$TaC_4H_{10}^+$	$R_{3,6\rightarrow4,10}$
$TaC_3H_8^+$	$TaC_4H_{10}^+ + H_2$	$R_{3,8\rightarrow4,10}$
	$TaC_4H_{12}^+$	$R_{3,8\rightarrow4,12}$
$TaC_3H_{10}^+$	$TaC_4H_{12}^+ + H_2$	$R_{3,10\rightarrow4,12}$
	$TaC_4H_{14}^+$	$R_{3,10\rightarrow4,14}$
$TaC_3H_{12}^+$	$TaC_4H_{14}^+ + H_2$	$R_{3,12\rightarrow4,14}$
$TaC_4H_8^+$	$TaC_5H_{12}^+$	$R_{4,8\rightarrow5,12}$
$TaC_4H_{10}^+$	$TaC_5H_{12}^+ + H_2$	$R_{4,10\rightarrow5,12}$
	$TaC_5H_{14}^+$	$R_{4,10\rightarrow5,14}$
$TaC_4H_{12}^+$	$TaC_5H_{14}^+ + H_2$	$R_{4,12\rightarrow5,14}$
	$TaC_5H_{16}^+$	$R_{4,12\rightarrow5,16}$
	$TaC_5H_{12}^+ + H_2$	$R_{4,10\rightarrow5,12}$
	$TaC_5H_{14}^+$	$R_{4,10\rightarrow5,14}$
	+He products (thermal dissociation)	
$TaC_2H_4^+$	$TaC_2H_2^+ + H_2 + He$	$D_{2,4\rightarrow2,2}$
$TaC_3H_6^+$	$TaC_3H_4^+ + H_2 + He$	$D_{3,6\rightarrow3,4}$
$TaC_2H_8^+$	$TaC_2H_6^+ + H_2 + He$	$D_{2,8\rightarrow2,6}$

Table S2. Derived rate constants and uncertainties for the indicated $TaR^+ + CH_4$ reactions. The min-max interval is taken as a 95% confidence interval. These intervals reflect the random error in the measurements; the absolute uncertainties can be obtained (as in Table 1) by convoluting an estimated additional 15% systematic uncertainty. Rows lacking a min or max value are upper or lower limits respectively. The shorthand $R_{x,y\rightarrow n,m}$ (as used in the main text) indicates $TaC_xH_y^+ + CH_4 \rightarrow TaC_nH_m^+ + C_{x+1-n}H_{y+4-m}$. The shorthand $D_{x,y\rightarrow n,m}$ indicates thermal dissociation reactions $TaC_xH_y^+ \rightarrow TaC_nH_m^+ + C_{x-n}H_{y-m}$

	300 K			400 K		
**Rate Constant**	**Min**	**Median**	**Max**	**Min**	**Median**	**Max**
$R_{0,0 \rightarrow 1,2}$	3.84E-10	4.24E-10	4.41E-10	3.90E-10	4.11E-10	4.27E-10
$R_{0,0 \rightarrow 1,4}$	1.51E-12	2.91E-12	3.62E-11		1.03E-12	4.33E-12
$R_{1,2 \rightarrow 2,4}$	2.37E-10	3.21E-10	3.56E-10	2.43E-10	2.90E-10	3.40E-10
$R_{1,2 \rightarrow 2,6}$	2.89E-10	3.42E-10	4.35E-10	1.66E-10	2.18E-10	2.73E-10
$R_{1,4 \rightarrow 2,6}$		4.05E-11	9.47E-10	3.10E-11	8.32E-11	3.61E-10
$R_{1,4 \rightarrow 2,8}$	3.92E-11	8.89E-11	1.40E-10	4.31E-11	7.20E-11	1.06E-10
$R_{2,2 \rightarrow 3,4}$	2.47E-10	2.60E-10	2.77E-10	1.87E-10	2.01E-10	2.14E-10
$R_{2,2 \rightarrow 3,6}$	9.64E-12	1.26E-11	1.58E-11	5.00E-12	6.72E-12	8.44E-12
$R_{2,4 \rightarrow 3,6}$	4.86E-10	5.56E-10	6.20E-10	4.14E-10	4.67E-10	5.17E-10
$R_{2,4 \rightarrow 3,8}$	2.40E-11	2.54E-10	3.60E-10		1.41E-10	2.77E-10
$R_{2,6 \rightarrow 3,8}$		6.10E-11	2.75E-10		2.74E-10	5.03E-10
$R_{2,6 \rightarrow 3,10}$	7.25E-10	9.39E-10		4.96E-10	7.22E-10	
$R_{2,8 \rightarrow 3,10}$	1.26E-12	1.73E-10	4.51E-10		1.14E-12	7.92E-12
$R_{2,8 \rightarrow 3,12}$	1.07E-12	1.73E-10	3.03E-10	2.20E-10	2.97E-10	3.61E-10
$R_{3,4 \rightarrow 4,6}$		1.15E-12	1.70E-12	2.20E-12	2.58E-12	3.08E-12
$R_{3,4 \rightarrow 4,8}$	1.24E-10	1.37E-10	1.48E-10	5.93E-11	6.81E-11	7.67E-11
$R_{3,6 \rightarrow 4,8}$	5.45E-10	6.79E-10	7.46E-10	4.69E-10	5.12E-10	5.72E-10
$R_{3,6 \rightarrow 4,10}$		2.64E-11	1.58E-10		7.96E-12	3.33E-11
$R_{3,8 \rightarrow 4,10}$	7.50E-13	1.40E-12	4.20E-10	5.00E-13	1.02E-12	2.02E-10
$R_{3,8 \rightarrow 4,12}$	1.32E-11	9.98E-10		5.50E-12	9.99E-10	
$R_{3,10 \rightarrow 4,12}$		8.48E-12	7.63E-11		1.74E-11	2.16E-10
$R_{3,10 \rightarrow 4,14}$		1.04E-12	1.67E-12		1.00E-12	1.02E-12
$R_{3,12 \rightarrow 4,14}$	4.37E-12	2.60E-11	9.67E-10	1.08E-12	1.87E-11	3.58E-11
$R_{4,8 \rightarrow 5,12}$	3.99E-10	6.30E-10		1.91E-10	5.51E-10	

$\mathbf{R}_{4,10 \rightarrow 5,12}$		**7.62E-12**	**9.94E-10**		**5.99E-12**	
$\mathbf{R}_{4,10 \rightarrow 5,14}$		**2.31E-11**	**5.29E-11**		**3.73E-12**	**2.70E-11**
$\mathbf{R}_{4,12 \rightarrow 5,14}$		**1.00E-12**	**5.66E-10**		**1.00E-12**	**2.69E-10**
$\mathbf{R}_{4,12 \rightarrow 5,16}$		**1.13E-12**	**3.70E-10**		**1.00E-12**	**3.70E-11**
$\mathbf{D}_{5,12 \rightarrow 4,8}$	**7.072968**	**206.1785**	**870.8202**	**1.278597**	**1975.592**	**4594.258**
$\mathbf{R}_{2,4^* \rightarrow 3,6}$		**9.95E-11**	**1.00E-10**	**8.84E-13**	**9.75E-11**	**1.00E-10**
$\mathbf{R}_{2,4^* \rightarrow 3,8}$		**1.12E-12**			**1.14E-12**	
$\mathbf{R}_{3,6^* \rightarrow 4,10}$	**2.01E-12**	**2.35E-10**		**5.11E-11**	**1.35E-10**	**9.89E-10**
$\mathbf{D}_{2,4 \rightarrow 2,2}$	**3.109262**	**17.26263**	**22.2522**	**11.03173**	**14.13659**	**20.2482**
$\mathbf{D}_{2,8 \rightarrow 2,6}$	**2.379707**	**55.37546**	**251.6882**		**1.756363**	**44.62536**
$\mathbf{D}_{3,6 \rightarrow 3,4}$		**10.4614**	**25.12827**	**10.4365**	**14.0609**	**20.22685**
$\mathbf{R}_{1,0 \rightarrow 2,2}$		**4.42E-10**			**4.66E-10**	
$\mathbf{D}_{4,8 \rightarrow 4,6}$		**1.224935**	**3.67797**	**1.342012**	**2.359605**	**3.173247**
$\mathbf{D}_{4,12 \rightarrow 3,8}$		**6.722291**	**32.64797**		**1.359414**	**177.0239**

	**500 K**			**600 K**		
**Rate Constant**	**Min**	**Median**	**Max**	**Min**	**Median**	**Max**
$\mathbf{R}_{0,0 \rightarrow 1,2}$	**4.01E-10**	**4.24E-10**	**4.49E-10**	**3.59E-10**	**4.02E-10**	**4.47E-10**
$\mathbf{R}_{0,0 \rightarrow 1,4}$		**1.46E-12**	**3.07E-12**		**1.01E-12**	**1.84E-12**
$\mathbf{R}_{1,2 \rightarrow 2,4}$	**2.24E-10**	**2.35E-10**	**2.53E-10**	**1.84E-10**	**2.17E-10**	**2.54E-10**
$\mathbf{R}_{1,2 \rightarrow 2,6}$	**1.31E-10**	**1.65E-10**	**1.72E-10**	**2.45E-11**	**8.35E-11**	**9.98E-11**
$\mathbf{R}_{1,4 \rightarrow 2,6}$		**1.04E-12**	**3.10E-12**		**1.06E-12**	**2.03E-11**
$\mathbf{R}_{1,4 \rightarrow 2,8}$	**8.93E-11**	**1.70E-10**	**5.18E-10**	**4.52E-11**	**6.62E-11**	**9.78E-11**
$\mathbf{R}_{2,2 \rightarrow 3,4}$	**1.28E-10**	**1.45E-10**	**1.56E-10**	**1.06E-10**	**1.16E-10**	**1.23E-10**
$\mathbf{R}_{2,2 \rightarrow 3,6}$	**2.10E-12**	**5.90E-12**	**1.02E-11**	**2.39E-12**	**4.62E-12**	**6.67E-12**
$\mathbf{R}_{2,4 \rightarrow 3,6}$	**4.35E-10**	**4.60E-10**	**4.93E-10**	**3.23E-10**	**4.09E-10**	**4.88E-10**

**$R_{2,4 \rightarrow 3,8}$**		**1.01E-12**	**2.05E-11**		**1.91E-11**	**1.52E-10**
**$R_{2,6 \rightarrow 3,8}$**	**1.58E-10**	**6.61E-10**	**8.86E-10**	**2.24E-10**	**6.78E-10**	**8.21E-10**
**$R_{2,6 \rightarrow 3,10}$**	**4.60E-12**	**3.39E-10**	**8.42E-10**	**1.90E-12**	**1.90E-10**	**2.96E-10**
**$R_{2,8 \rightarrow 3,10}$**		**1.04E-12**	**2.61E-12**		**1.27E-11**	**5.28E-11**
**$R_{2,8 \rightarrow 3,12}$**	**1.38E-10**	**1.95E-10**	**5.80E-10**	**1.31E-10**	**1.78E-10**	**2.81E-10**
**$R_{3,4 \rightarrow 4,6}$**	**3.64E-12**	**3.96E-12**	**4.41E-12**	**5.22E-12**	**6.58E-12**	**7.84E-12**
**$R_{3,4 \rightarrow 4,8}$**	**3.31E-11**	**3.63E-11**	**3.98E-11**	**1.45E-11**	**1.82E-11**	**2.20E-11**
**$R_{3,6 \rightarrow 4,8}$**	**3.38E-10**	**3.65E-10**	**3.87E-10**	**2.00E-10**	**2.34E-10**	**2.53E-10**
**$R_{3,6 \rightarrow 4,10}$**		**5.18E-12**	**1.67E-11**		**3.09E-12**	**1.86E-11**
**$R_{3,8 \rightarrow 4,10}$**	**5.00E-13**	**1.04E-12**	**5.68E-11**		**2.73E-12**	**1.70E-10**
**$R_{3,8 \rightarrow 4,12}$**		**9.92E-10**		**1.03E-12**	**5.97E-10**	**8.02E-10**
**$R_{3,10 \rightarrow 4,12}$**		**1.44E-12**	**6.96E-10**		**1.06E-12**	**3.58E-10**
**$R_{3,10 \rightarrow 4,14}$**	**1.10E-12**	**1.72E-12**	**1.44E-10**	**1.05E-12**	**1.66E-12**	**3.61E-10**
**$R_{3,12 \rightarrow 4,14}$**	**1.18E-12**	**1.08E-11**	**3.32E-11**		**1.52E-11**	**5.03E-11**
**$R_{4,8 \rightarrow 5,12}$**	**3.23E-11**	**9.16E-10**		**3.62E-11**	**8.51E-10**	
**$R_{4,10 \rightarrow 5,12}$**		**1.34E-12**	**9.98E-10**	**2.69E-12**	**1.37E-10**	
**$R_{4,10 \rightarrow 5,14}$**		**1.82E-11**	**4.52E-11**		**1.01E-12**	**3.46E-12**
**$R_{4,12 \rightarrow 5,14}$**		**1.00E-12**	**1.12E-12**		**1.00E-12**	**3.49E-10**
**$R_{4,12 \rightarrow 5,16}$**		**1.00E-12**	**1.92E-12**		**1.00E-12**	**2.22E-10**
**$D_{5,12 \rightarrow 4,8}$**	**69.05054**	**33355.62**	**40703.66**	**28211.61**	**515010.7**	**870069.5**
**$R_{2,4^* \rightarrow 3,6}$**		**1.00E-12**			**1.10E-12**	**9.84E-10**
**$R_{2,4^* \rightarrow 3,8}$**		**5.13E-10**			**4.39E-10**	
**$R_{3,6^* \rightarrow 4,10}$**		**3.93E-11**		**5.11E-12**	**7.93E-11**	**9.92E-10**
**$D_{2,4 \rightarrow 2,2}$**	**22.07001**	**25.18157**	**29.15214**	**94.38698**	**136.0662**	**204.1778**
**$D_{2,8 \rightarrow 2,6}$**		**1.174942**	**5.727437**		**30.62541**	**236.3036**
**$D_{3,6 \rightarrow 3,4}$**	**46.67644**	**53.9379**	**63.86756**	**189.109**	**210.5945**	**249.526**

**$R_{1,0 \rightarrow 2,2}$**	**5.00E-13**	**1.00E-10**	**1.00E-10**	**1.11E-12**	**7.05E-11**	**1.00E-10**
**$D_{4,8 \rightarrow 4,6}$**		**2.638103**	**3.922185**	**4.72329**	**7.01598**	**11.72938**
**$D_{4,12 \rightarrow 3,8}$**		**255.5004**	**404.588**		**99.13049**	**454.4912**

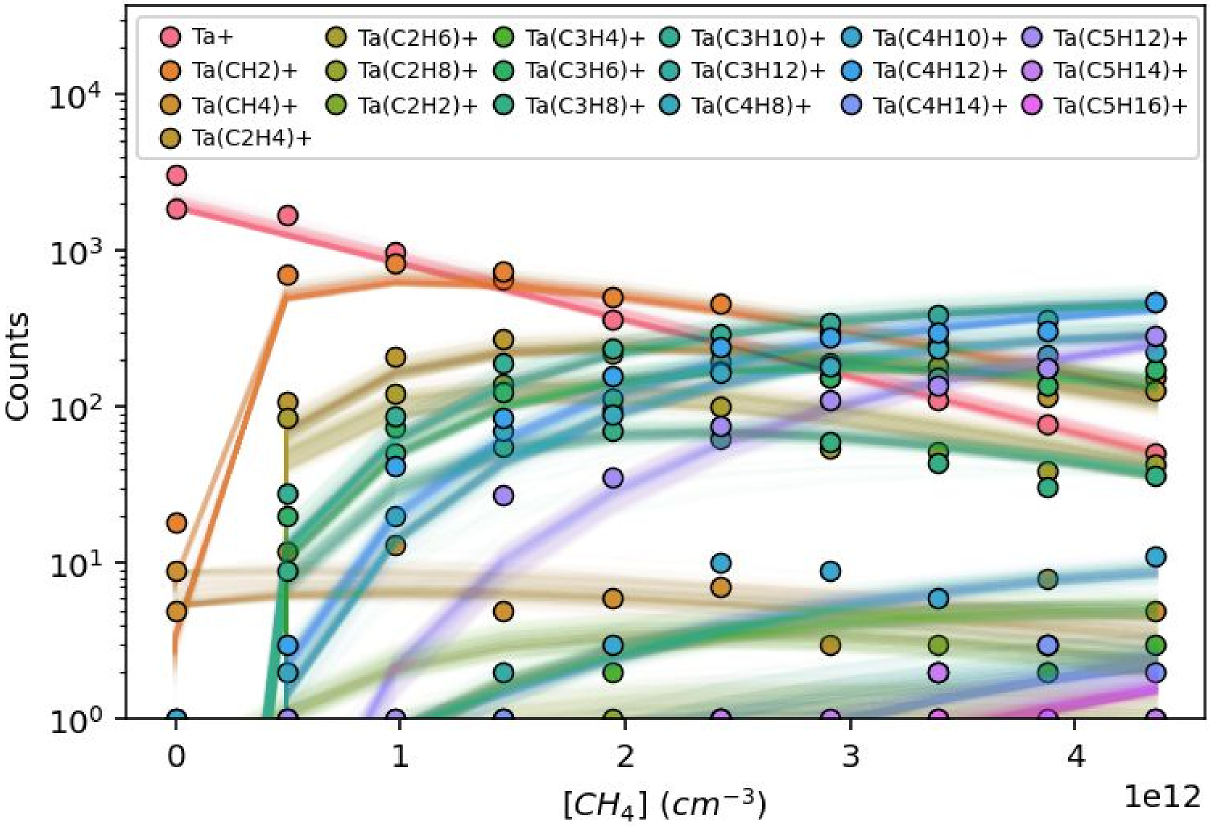

Figure S1. Representative experimental ion abundances and modeled fits for a single experiment. We place this example here to justify the choice of the non-standard data presentation in Figure 1.

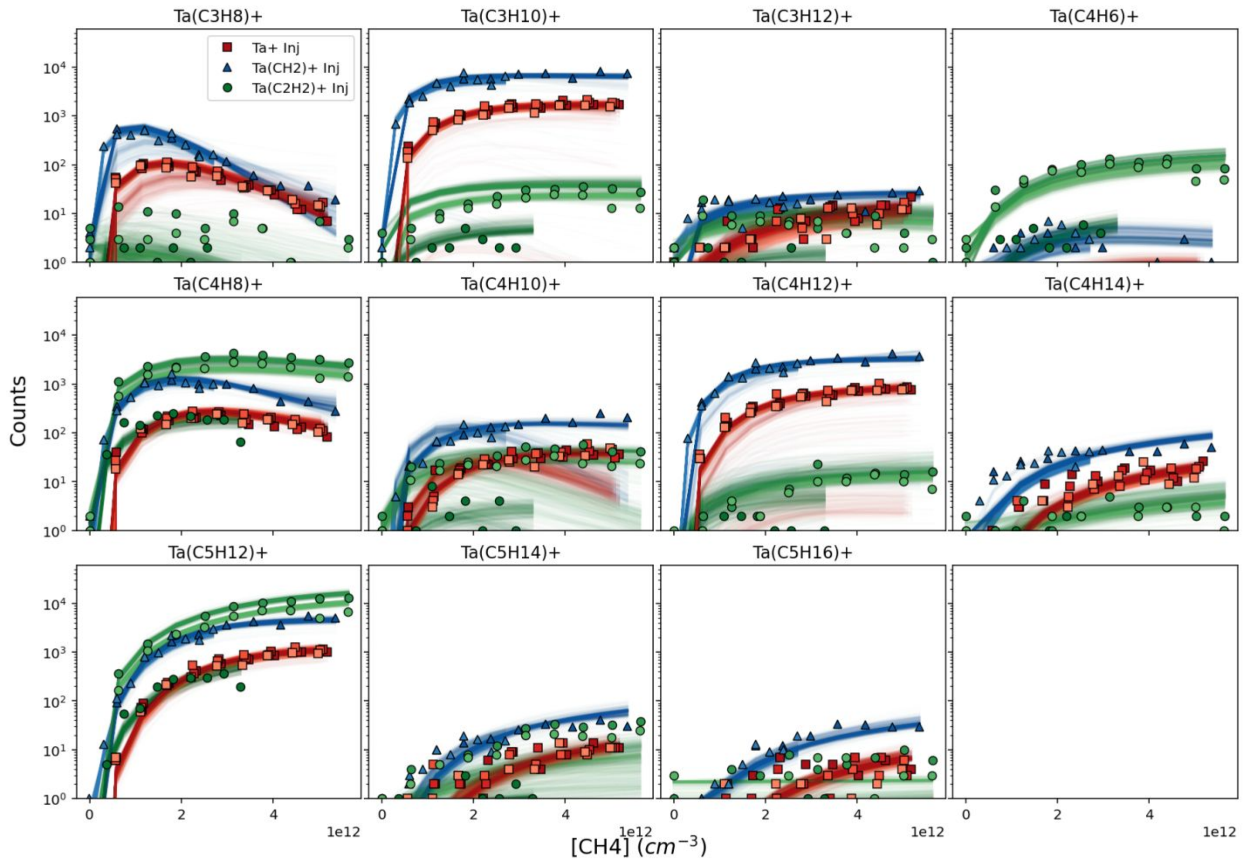

Figure S2. As Figure 1, showing species not included in that figure for the fits performed at 300 K. Observed ion abundances from multiple experiments compared to modeled fits. Data from 9 experiments across 3 experimental conditions (injection of either Ta^+ (red squares) , $TaCH_2^+$ (blue triangles) , or $TaC_2H_2^+$ (green circles)) are shown in each panel; individual experiments are distinguished by shade. Curves are modeled abundances for varying sets of rate constants which reproduced the experimental values within an acceptable likelihood (see text).

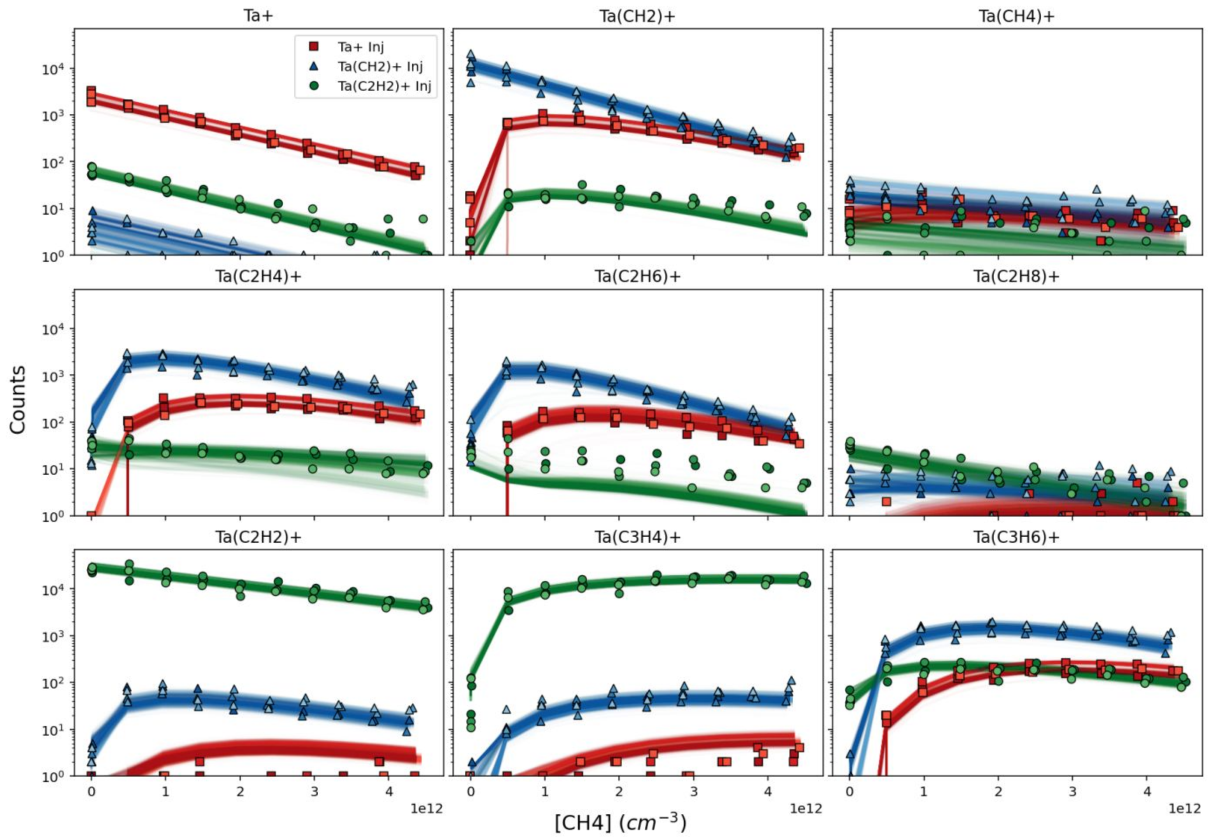

Ta+
Ta(CH2)+
Ta(CH4)+
Ta+ Inj
Ta(CH2)+ Inj
Ta(C2H2)+ Inj
Ta(C2H4)+
Ta(C2H6)+
Ta(C2H8)+
Ta(C2H2)+
Ta(C3H4)+
Ta(C3H6)+
Counts
[CH4] (cm^{-3})
1e12

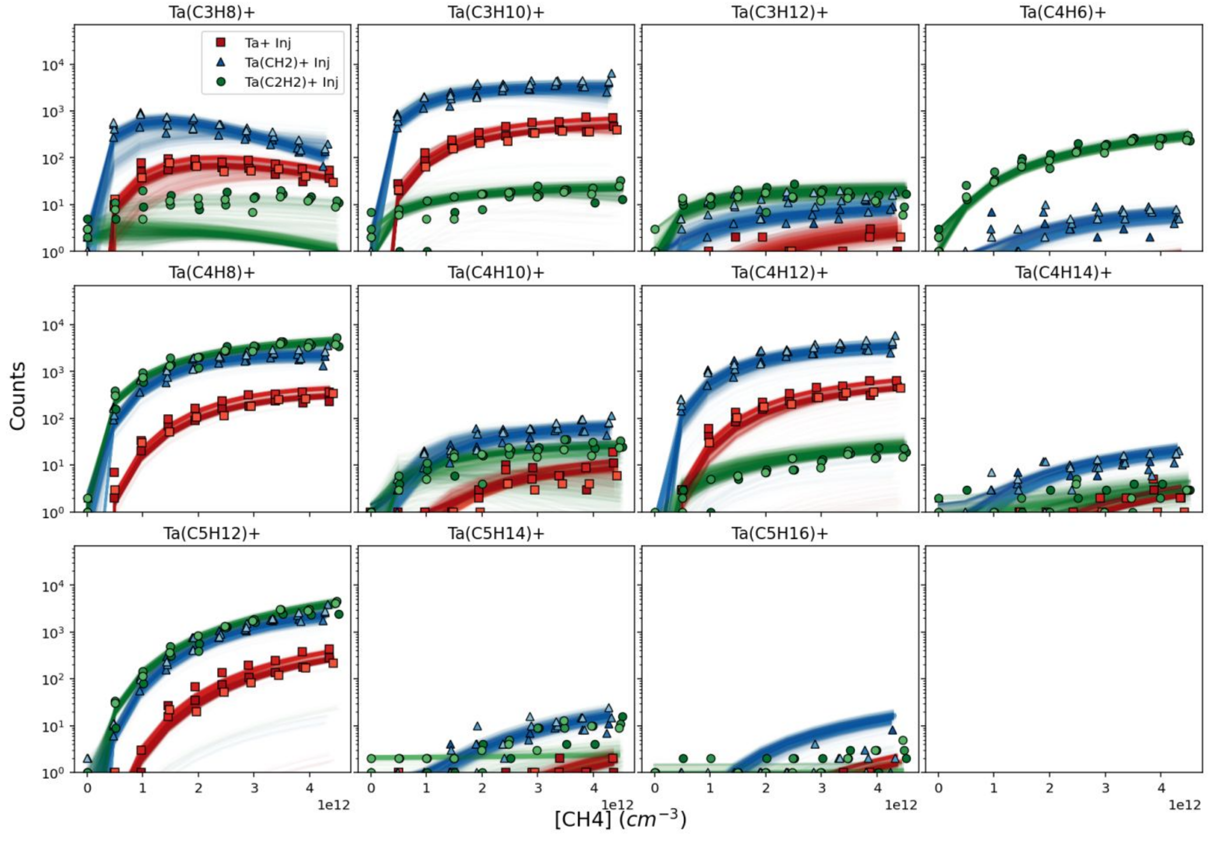

Ta(C3H8)+
Ta(C3H10)+
Ta(C3H12)+
Ta(C4H6)+
Ta+ Inj
Ta(CH2)+ Inj
Ta(C2H2)+ Inj
Ta(C4H8)+
Ta(C4H10)+
Ta(C4H12)+
Ta(C4H14)+
Ta(C5H12)+
Ta(C5H14)+
Ta(C5H16)+
Counts
[CH4] (cm^{-3})
1e12

Figure S3. As Figure S2 for data at 400K. Observed ion abundances from multiple experiments compared to modeled fits. Data from 10 experiments across 3 experimental conditions (injection of either Ta^+ (red squares) , $TaCH_2^+$ (blue triangles) , or $TaC_2H_2^+$ (green circles)) are shown in each panel; individual experiments are distinguished by shade. Curves are modeled abundances for varying sets of rate constants which reproduced the experimental values within an acceptable likelihood (see text).

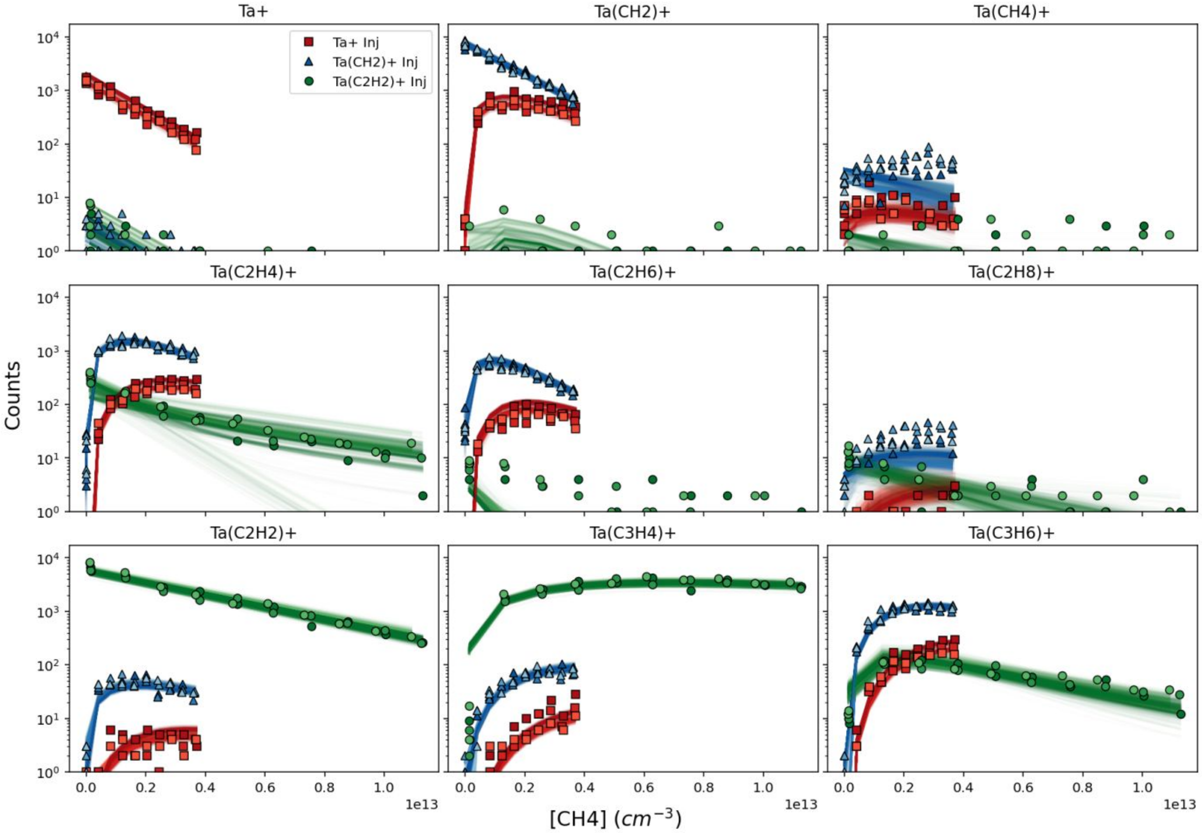
Ta+
Ta(CH2)+
Ta(CH4)+
Ta(C2H4)+
Ta(C2H6)+
Ta(C2H8)+
Ta(C2H2)+
Ta(C3H4)+
Ta(C3H6)+
Ta+ Inj
Ta(CH2)+ Inj
Ta(C2H2)+ Inj
Counts
[CH4] (cm^{-3})
1e13

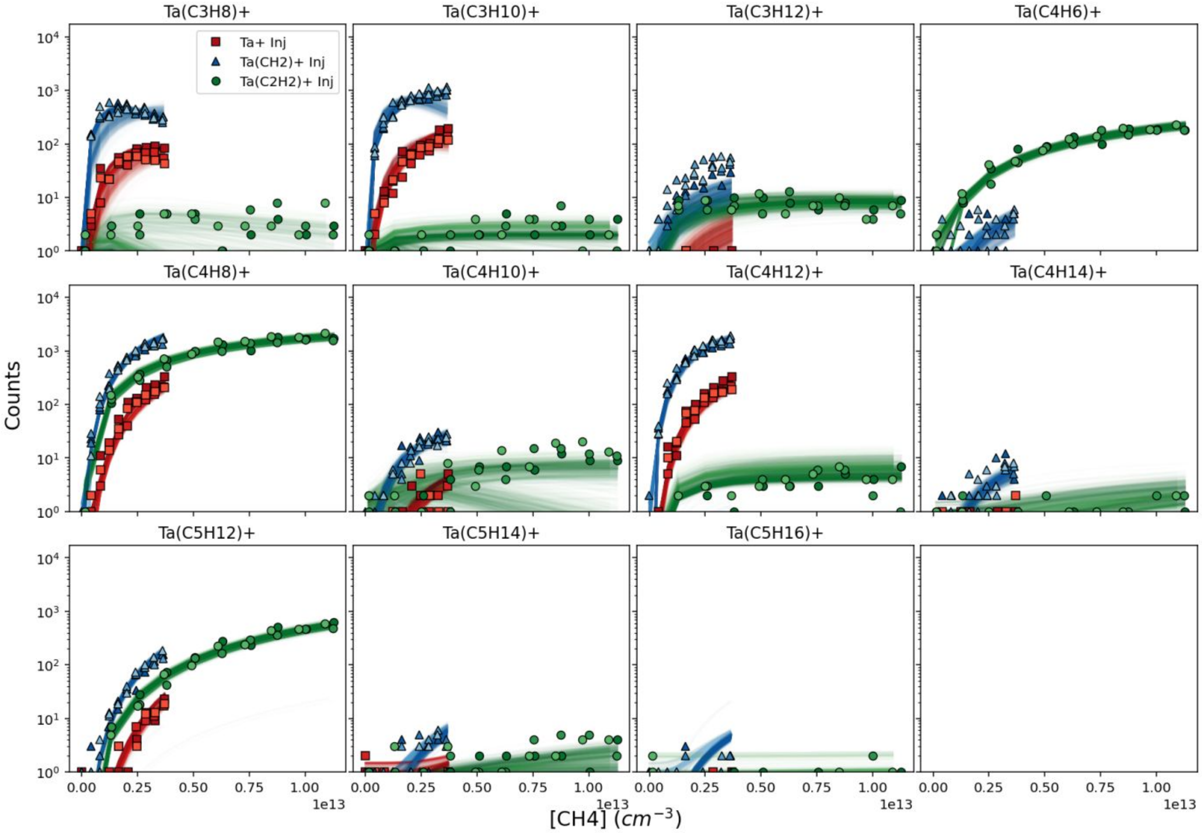
Ta(C3H8)+
Ta(C3H10)+
Ta(C3H12)+
Ta(C4H6)+
Ta(C4H8)+
Ta(C4H10)+
Ta(C4H12)+
Ta(C4H14)+
Ta(C5H12)+
Ta(C5H14)+
Ta(C5H16)+
Ta+ Inj
Ta(CH2)+ Inj
Ta(C2H2)+ Inj
Counts
[CH4] (cm^{-3})
1e13

Figure S4. As Figure S2 for data at 500K. Observed ion abundances from multiple experiments compared to modeled fits. Data from 10 experiments across 3 experimental conditions (injection of either Ta^+ (red squares) , $TaCH_2^+$ (blue triangles) , or $TaC_2H_2^+$ (green circles)) are shown in each panel; individual experiments are distinguished by shade. Curves are modeled abundances for varying sets of rate constants which reproduced the experimental values within an acceptable likelihood (see text).

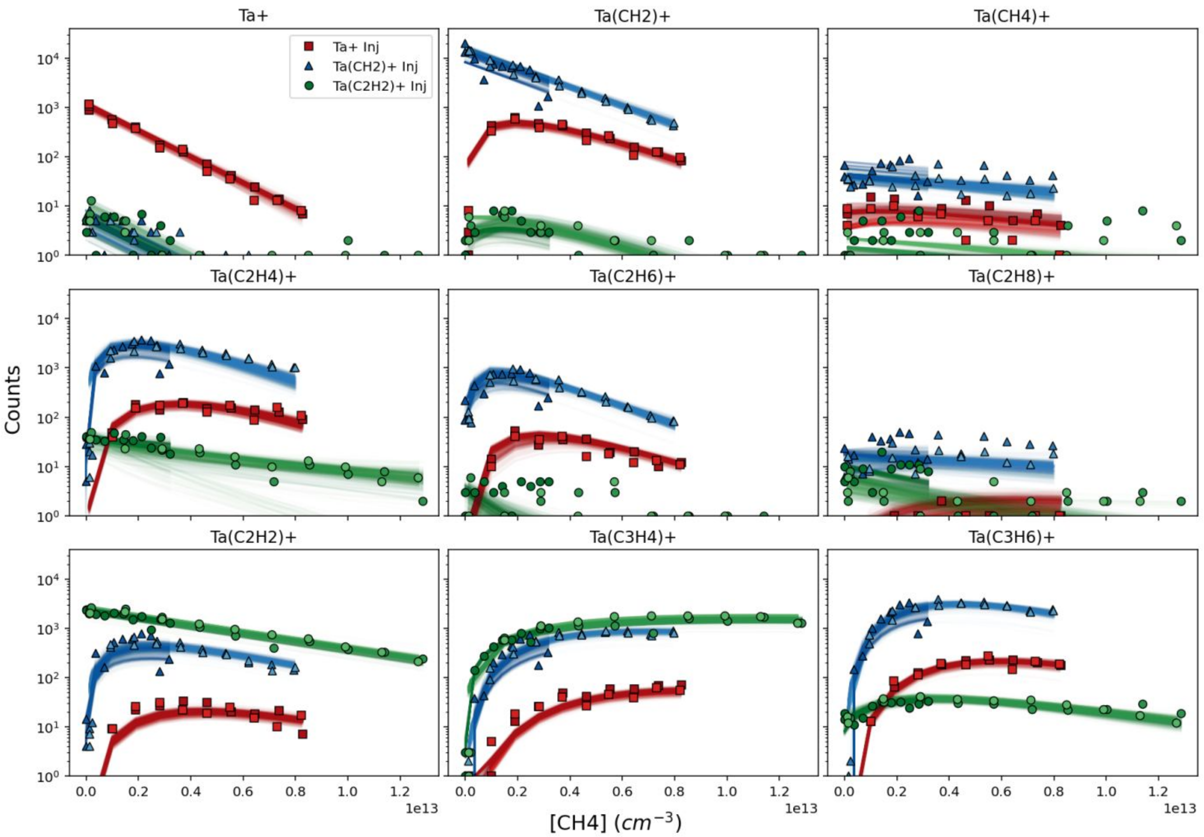

Ta+
Ta(CH2)+
Ta(CH4)+
Ta(C2H4)+
Ta(C2H6)+
Ta(C2H8)+
Ta(C2H2)+
Ta(C3H4)+
Ta(C3H6)+
Ta+ Inj
Ta(CH2)+ Inj
Ta(C2H2)+ Inj
Counts
[CH4] (cm^{-3})
1e13

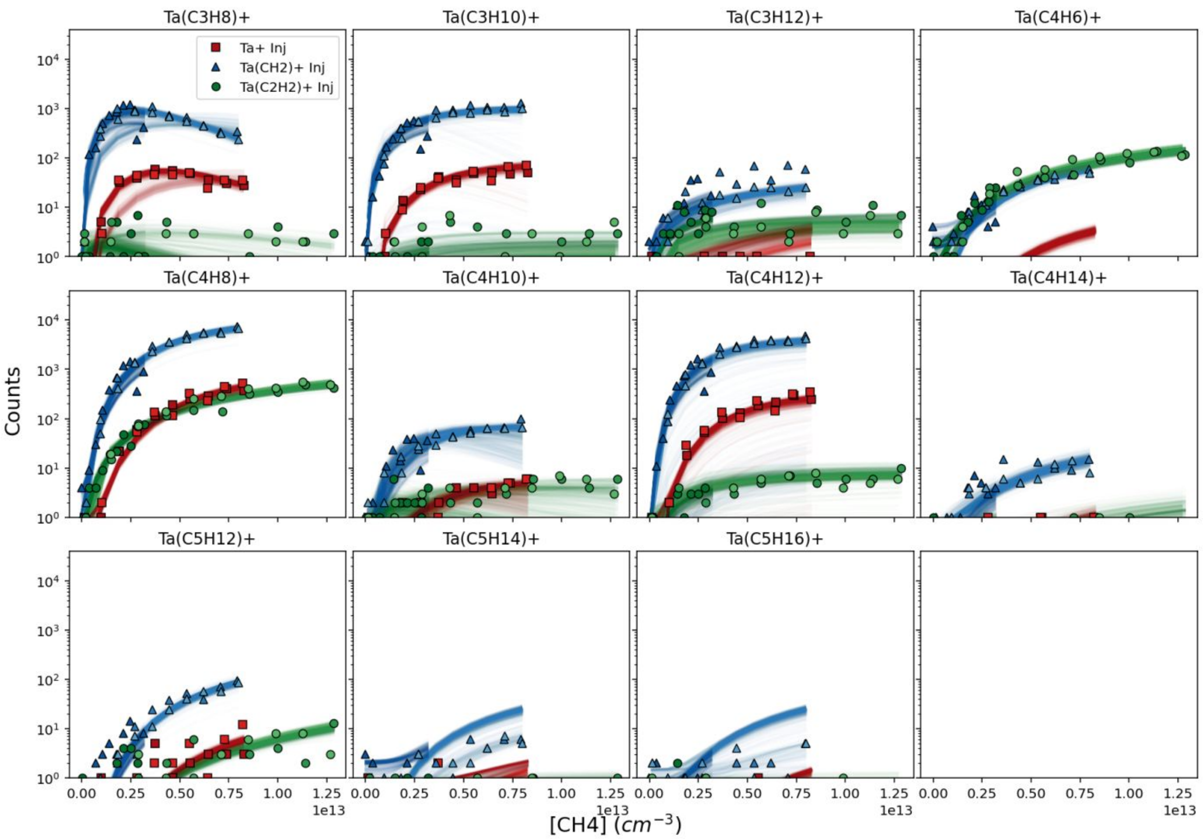

Ta(C3H8)+
Ta(C3H10)+
Ta(C3H12)+
Ta(C4H6)+
Ta(C4H8)+
Ta(C4H10)+
Ta(C4H12)+
Ta(C4H14)+
Ta(C5H12)+
Ta(C5H14)+
Ta(C5H16)+
Ta+ Inj
Ta(CH2)+ Inj
Ta(C2H2)+ Inj
Counts
[CH4] (cm^{-3})
1e13

Figure S5. As Figure 1 for data at 600K. Observed ion abundances from multiple experiments compared to modeled fits. Data from 8 experiments across 3 experimental conditions (injection of either Ta^+ (red squares) , $TaCH_2^+$ (blue triangles) , or $TaC_2H_2^+$ (green circles)) are shown in each panel; individual experiments are distinguished by shade. Curves are modeled abundances for varying sets of rate constants which reproduced the experimental values within an acceptable likelihood (see text).

Additional Rate Constant Figures

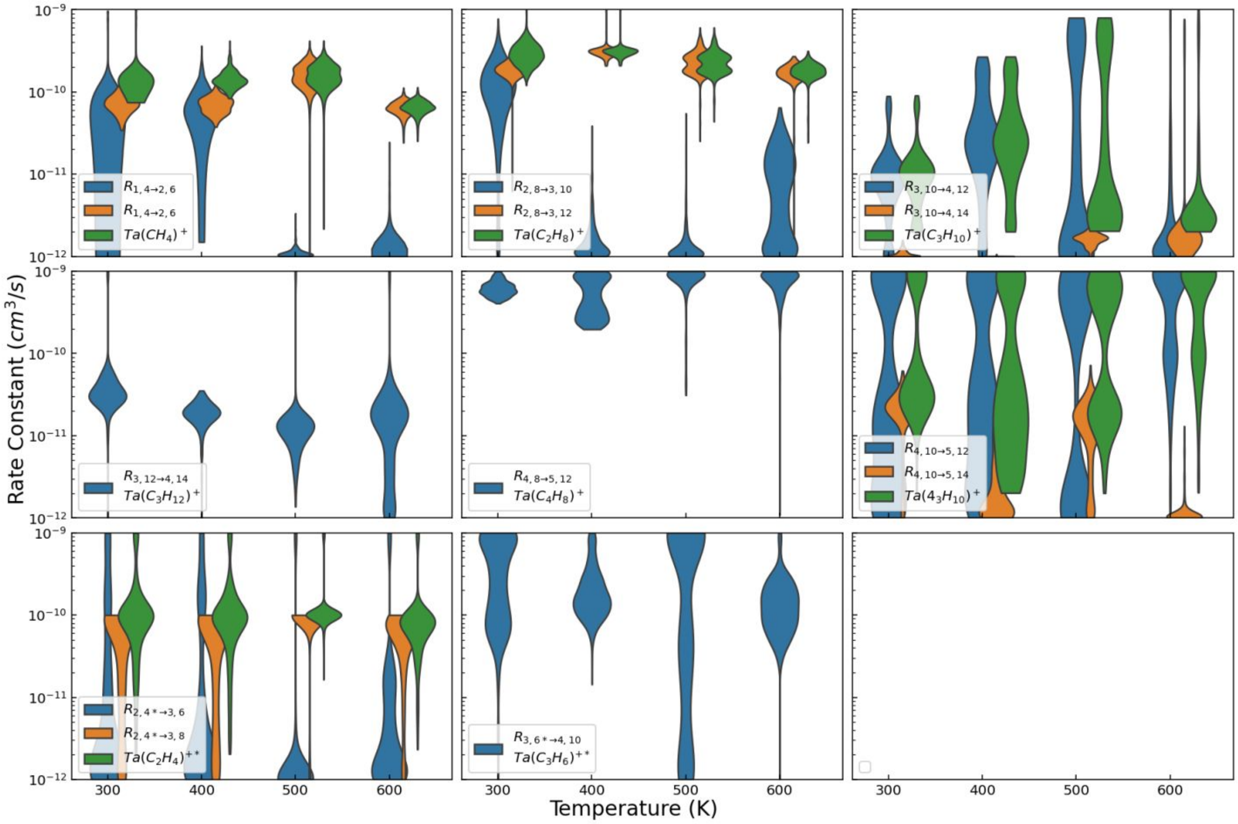

Figure S6. Derived rate constants for dehydrogenation reactions (blue), association processes (orange), and the summed total rate constant (green) for the indicated reactions. All measurements are at 300, 400, 500, or 600 K, with offsets to limit overlap.

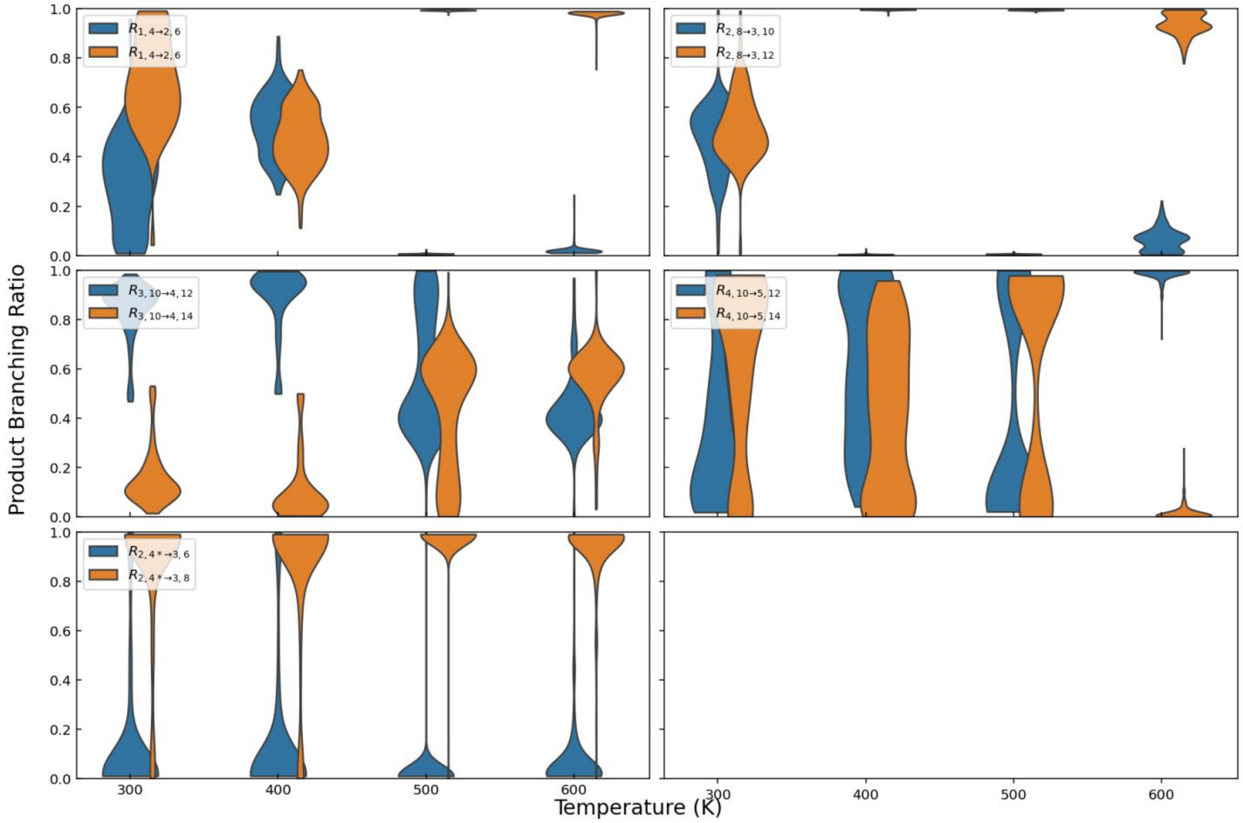

Figure S7. Derived product branching ratios for dehydrogenation reactions (blue) and association processes for the indicated reactions. All measurements are at 300, 400, 500, or 600 K with offsets to limit overlap

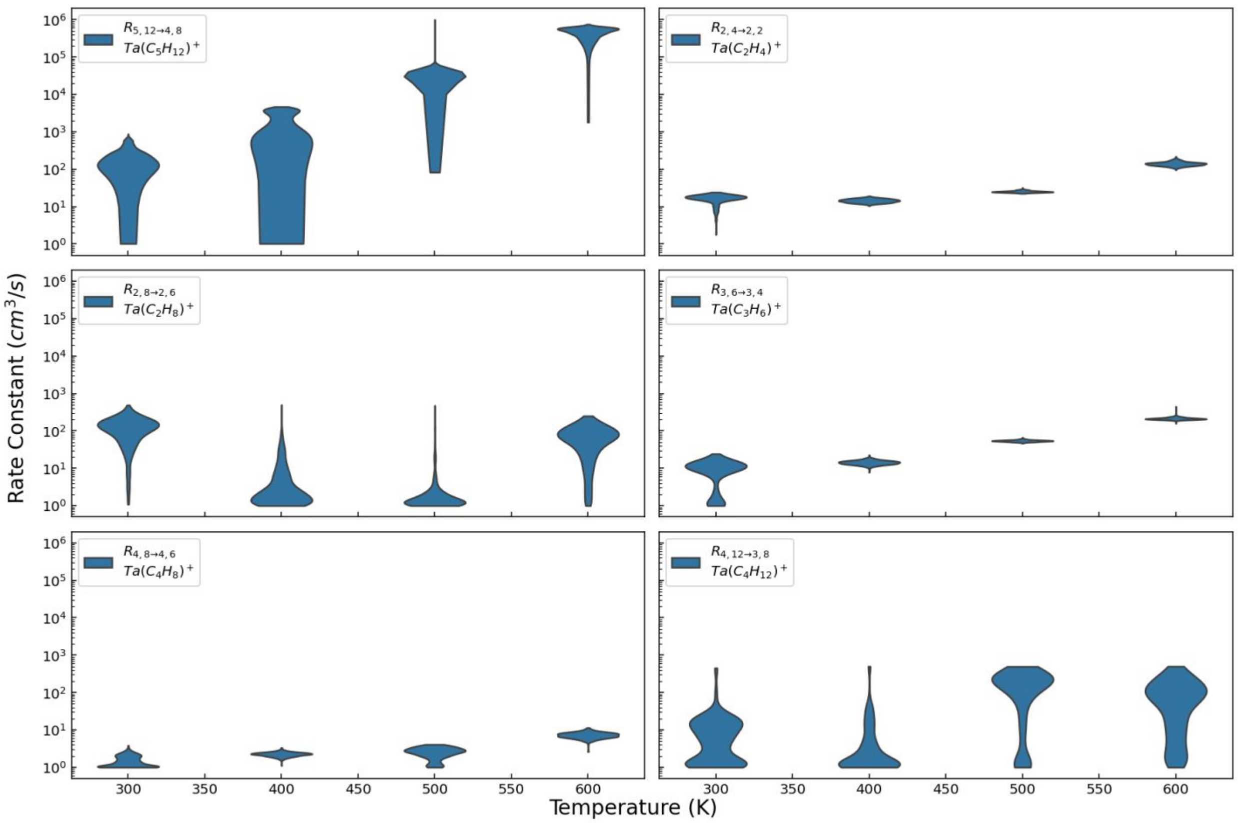

Figure S8. Derived rate constants for unimolecular rate constants for the indicated reactions. All measurements are at 300, 400, 500, or 600 K, with the blue and green offset to prevent overlap.

II. Analysis of results of Simon et al.

The elegant experiments of Simon et al. strongly suggest that the $HHTa(C_2H_2)^+$ isomer is formed from the sequential reactivity initiated by $Ta^+ + CH_4$, but do not report rate constants. Inspection of Figure 1 shows Ta^+, $TaCH_2^+$, $TaC_2H_4^+$, $TaC_3H_6^+$, and $TaC_4H_8^+$ in the relative abundances shown in Table S1 after an undefined extent of reaction with methane. Assuming only sequential dehydrogenation reactions occur and no discrimination in detection efficiency between masses, the abundances define the relative rate constants for each species. Defining the $Ta^+ + CH_4$ rate constant at 3.9×10^{-10} cm^3 s^{-1}, as found here and previously, yields the rate constants for the other species shown in Table 2. The rate constant of $TaCH_2^+ + CH_4$ is somewhat larger than found here, as expected due to the difference in pressure regimes (association of $TaCH_2^+ + CH_4$ occurs readily and competes with dehydrogenation) but the dehydrogenation rate constants for $TaC_2H_4^+$ and $TaC_3H_6^+$ are much *smaller* than found here. The ICR experiment of Irikura and Beauchamp similarly reports smaller rate constants for those reactions, while the ion trap experiment of Eckhard and the SIFT experiment of Shayesteh show rate constants much more similar to the present results. There is a trend for the ICR experiments,

in which the Ta^+ reactants have not been thermalized through collisions with a buffer gas, to show smaller rate constants for the $TaC_2H_4^+$ and $TaC_3H_6^+$ dehydrogenations.

III. $Ta^+ + CH_4 \rightarrow TaCH_2^+ + H_2$ exothermicity from equilibrium measurements

The measured rate constants for the reverse reaction $TaCH_2^+ + H_2 \rightarrow Ta^+ + CH_4$ and derived equilibrium constants for $Ta^+ + CH_4 \leftrightarrow TaCH_2^+ + H_2$ appear in Table S2 and Figure S9.

Table S2. Measured rate constants for $TaCH_2^+ + H_2 \rightarrow Ta^+ + CH_4$ and corresponding derived equilibrium constant for $Ta^+ + CH_4 \leftrightarrow TaCH_2^+ + H_2$.

T (K)	k ($cm^3\ s^{-1}$)	K
300	$8.4 \pm 2 \times 10^{-13}$	452±95
400	$1.3 \pm 0.1 \times 10^{-12}$	292±45
500	$1.6 \pm 0.2 \times 10^{-12}$	259±30
600	$1.6 \pm 0.1 \times 10^{-12}$	248±50

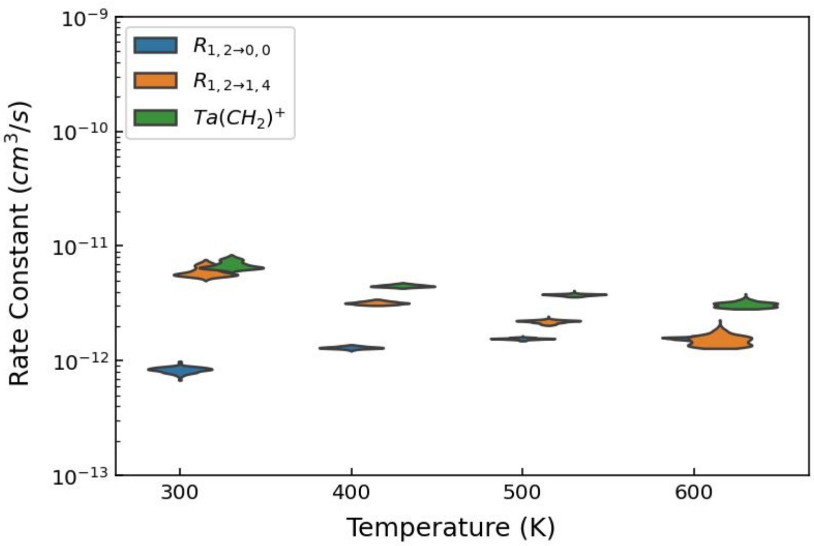

Figure S9. Experimentally derived total (green) rate constants for $TaCH_2^+ + H_2$ and partial rate constants for $TaCH_2^+ + H_2 \rightarrow Ta^+ + CH_4$ (blue) and $TaCH_2^+ + H_2 \rightarrow TaCH_4^+$ (orange) as a function of temperature.

The derived rate constants were determined using the SKA methodology described in the main text. The association reaction dominates at lower temperatures. If the bimolecular $R_{1,2\rightarrow0,0}$ and the association reaction $R_{1,2\rightarrow1,4}$ directly compete, the resulting $R_{1,2\rightarrow0,0}$ rate constant would be lowered relative to that obtained in a low-pressure environment and not appropriate for use in the below thermodynamic analysis. We make the assumption that here direct competition between the reactions is minimal as only "high" energy reactions can lead to an endothermic bimolecular product while association will be dominated by "low" energy reactions that must dispose of the smallest amount of energy to be stabilized to dissociation back to reactants.

A 2nd-law analysis of the data (Figure S10) indicates a reaction thermicity at 298.15 K of $\Delta_r H^o_{298.15K}$ = -3 ± 1 kJ mol^{-1}, but the linear fit to the data is somewhat unsatisfying.

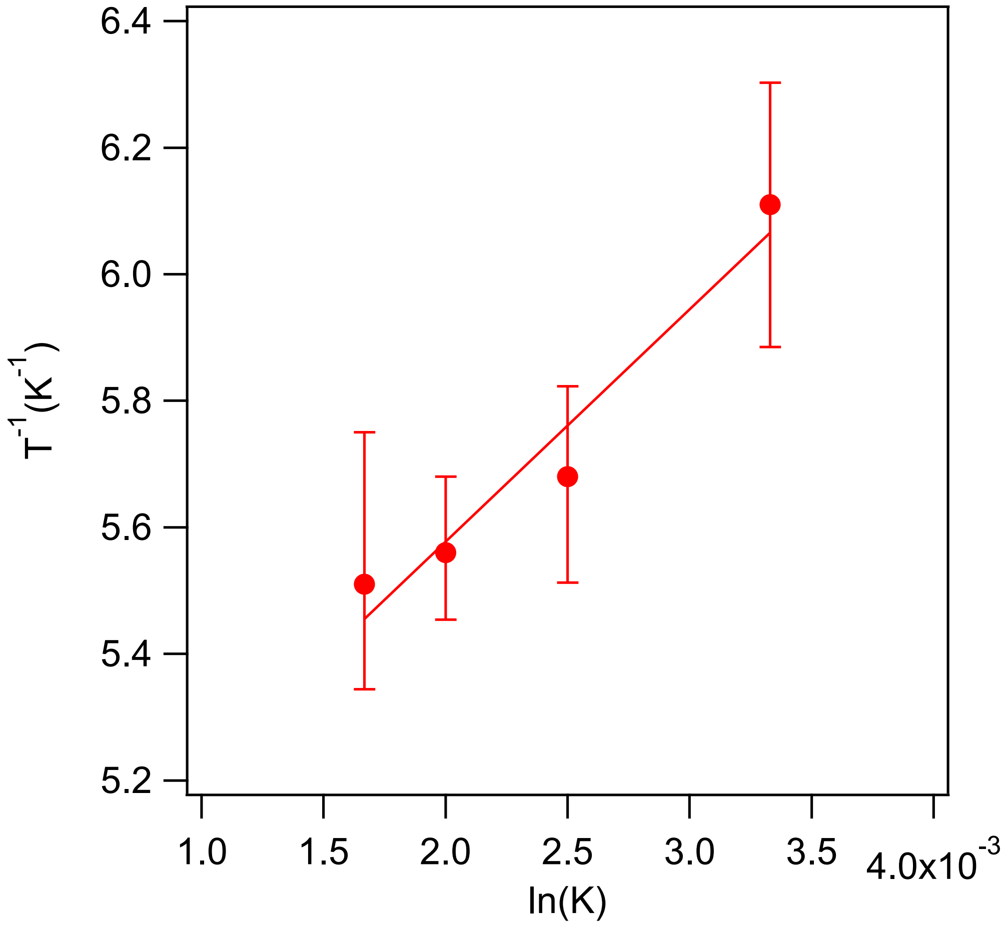

Figure S10. 2nd Law analysis of the $Ta^+ + CH_4$ ←→ $TaCH_2^+ + H_2$ equilibrium. The slope indicates $\Delta_r H^o_{298.15K}$ = -3 ± 1 kJ mol^{-1}.

A 3rd law analysis requires entropies and enthalpies. Those of Ta^+, H_2, and CH_4 as a function of temperature are compiled in the NIST/JANAF Thermochemical tables. Those for $TaCH_2^+$ are estimated following the methodology summarized previously [Nicholas S. Shuman, Thomas M. Miller, Shaun G. Ard, and Albert A. Viggiano *Kinetics of associative detachment of* O^- + N_2 *and dissociative attachment of* e^- + N_2O *up to 1300 K: Chemistry relevant to modeling of transient luminous events* Physical Chemistry Chemical Physics 25 31917-31927 (2023) DOI: 10.1039/d3cp03856d] and using vibrational modes calculated at the B3LYP/def2-TZVP level for the triplet ground state and are shown in Table S3. The resulting $\Delta_r H^o_{298.15K}$ (Figure S11) are inconsistent as a function of temperature, indicating an error in either the calculated thermodynamic quantities or in the measured equilibrium constants. Adjusting the calculated $S^o(TaCH_2^+)$ by 11 J mol^{-1} K^{-1} (about a 4% adjustment) yields a consistent value of $\Delta_r H^o_{298.15K}$ = -

2.5 kJ mol^{-1}, consistent with the 2nd law analysis. The need for adjustment to the calculated entropy is also visualized in Figure S12, noting the slope in the Gibbs free energy of reaction derived from experiment compared to that calculated with or without the adjustment.

Table S3. Enthalpies and entropies of the indicated species along with literature 0 K heats of formation where available.

	$TaCH_2^+$		H_2		Ta^+		CH_4	
T(K)	H(kJ mol^{-1})	S(J mol^{-1} K^{-1})	H(kJ mol^{-1})	S(J mol^{-1} K^{-1})	H(kJ mol^{-1})	S(J mol^{-1} K^{-1})	H(kJ mol^{-1})	S(J mol^{-1} K^{-1})
300	12.1	271	8.52	130.9	6.24	185.4	10.09	186.5
400	17.1	285	11.43	139.2	8.34	191.4	13.89	197.4
500	22.6	297	14.35	145.7	10.51	196.2	18.22	207.0
600	28.3	308	17.28	151.0	12.77	200.3	23.15	216.0
$\Delta_f H(0K)$			0		1543		-66.55	

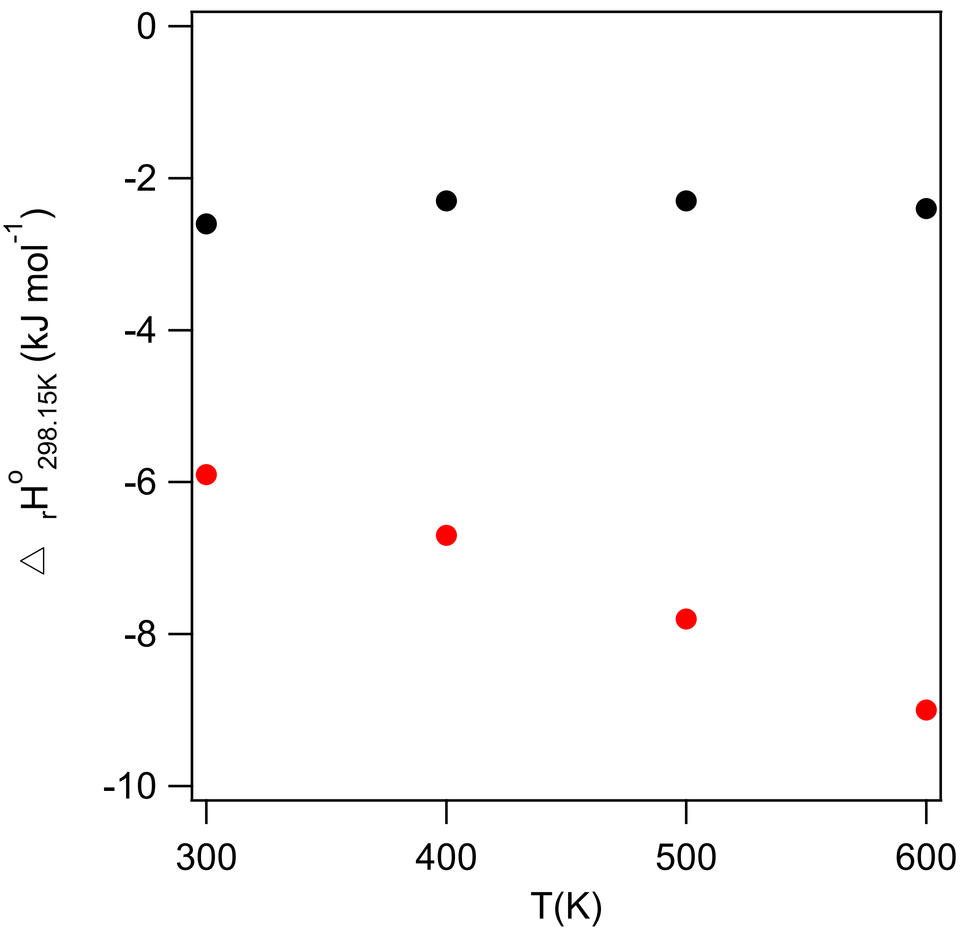

Figure S11. 3rd Law analysis of the $Ta^+ + CH_4 \leftarrow\rightarrow TaCH_2^+ + H_2$ equilibrium using $S^o(TaCH_2^+)$ as calculated (red points) or adjusted by 0.0011 kJ mol^{-1} K^{-1} (black points).

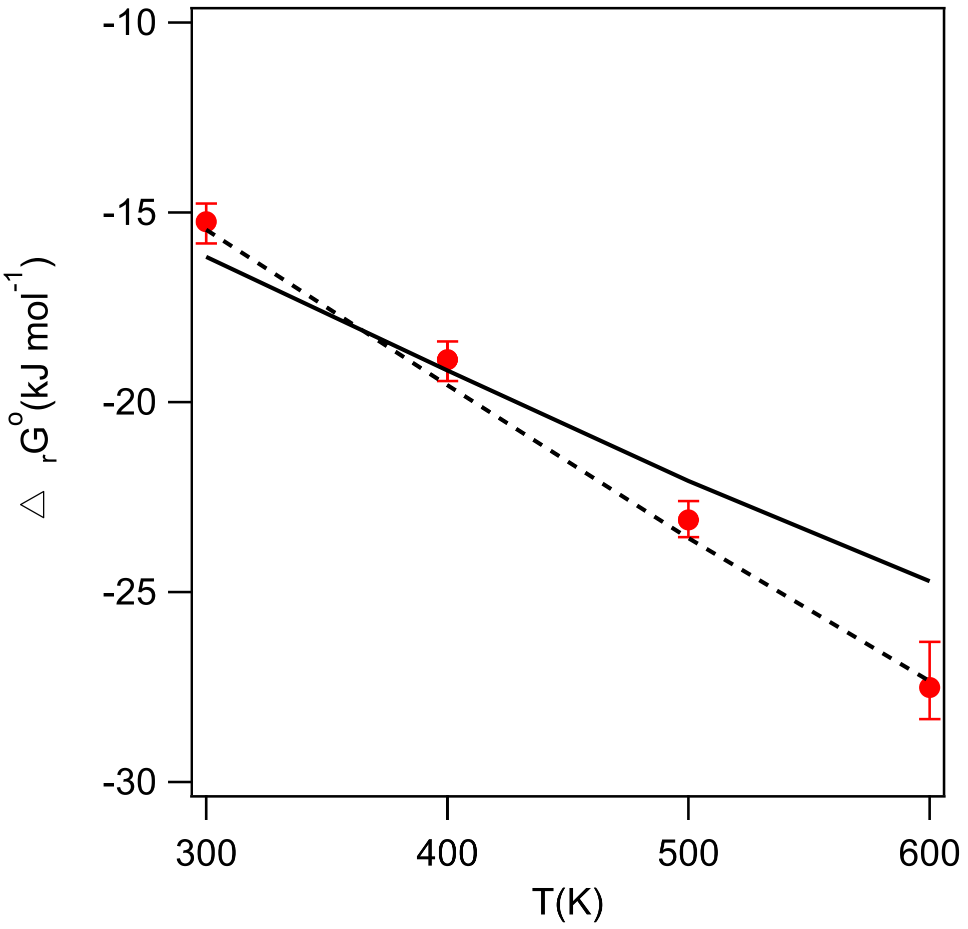

Figure S12. Empirical Gibbs energies of reaction (red points) for $Ta^+ + CH_4 \leftarrow\rightarrow TaCH_2^+ + H_2$ compared to those calculated (solid black line, $\Delta_rH^o(0K)$ = -10 kJ mol^{-1}) or using $S^o(TaCH_2^+)$ adjusted by 0.0011 kJ mol^{-1} K^{-1} (dashed black line, $\Delta_rH^o(0K)$ = -7 kJ mol^{-1}).

Using the adjusted entropy, the measured equilibrium constants are well fit assuming $\Delta_rH^o(0K)$ = -7 ±2 kJ mol^{-1} (Figure S13), which corresponds to $\Delta_rH^o(298.15K)$ = -2.7 ±2 kJ mol^{-1}, in agreement with the 2nd and 3rd law analyses. To encompass the possibility that the error was not in the calculated $S^o(TaCH_2^+)$, but a systematic artifact in the experiment, we convolute that error with the 3 kJ mol^{-1} difference in best-fit $\Delta_rH^o(0K)$ between using the as-calculated and adjusted $S^o(TaCH_2^+)$ to arrive at a ±4 kJ mol^{-1} uncertainty in the final value.

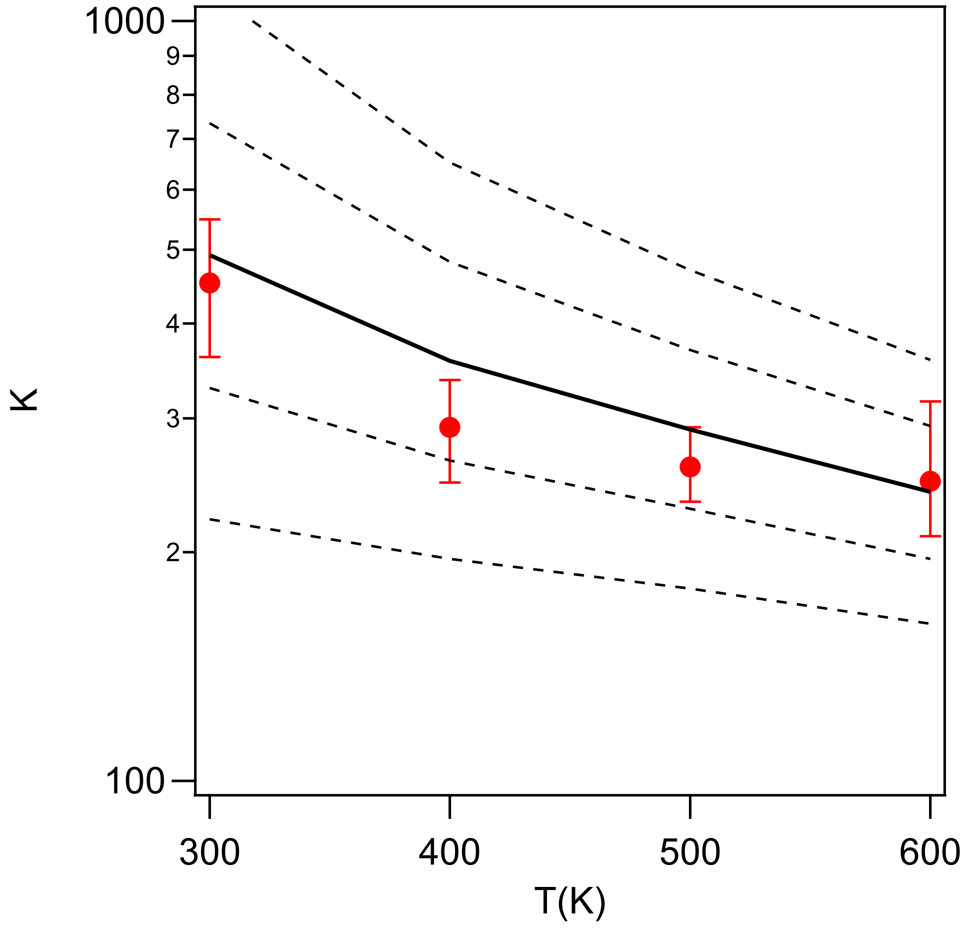

Figure S13. Empirical equilibrium constants (red points) for $Ta^+ + CH_4 \leftrightarrow TaCH_2^+ + H_2$ as a function of temperature compared to those calculated assuming $S^0(TaCH_2^+)$ as calculated adjusted by 0.0011 kJ mol^{-1} K^{-1} and $TaCH_2^+$ $\Delta_r H^0$(0K) = -7 kJ mol^{-1} (solid black line) or ±1 kJ mol^{-1} or ±2 kJ mol^{-1} of that value (dashed black lines).

Using enthalpies of formation from the Active Thermochemical Tables:

Species	$\Delta_f H^o$(0K) (kJ mol^{-1})
CH_4	-66.540 ±0.043
CH_2	391.076 ±0.094
H_2	0

giving a CH_2-H_2 0K BDE of 457.616 ±0.103 kJ mol^{-1}, the enthalpy of reaction implies:

Quantity	0 K (kJ mol^{-1})	298.15 K (kJ mol^{-1})
Ta^+-CH_2 BDE	465 ±4	469 ±4
$\Delta_f H^o$ $TaCH_2^+$	1469 ±4	1473 ±4

$\Delta_r H^o$ $Ta^+ + CH_4 \rightarrow TaCH_2^+ + H_2$	-7 ±4	-3 ±4

Comparing to literature values yields good agreement:

	Ta^+-CH_2 0K
This work	465 ±4
Parke et al. [a]	464 ±3
Irikura and Goddard [b]	481 ±21
Metz [c]	> 450 ±5

[a] Parke, L. G.; Hinton, C. S.; Armentrout, P. B. Experimental and Theoretical Studies of the Activation of Methane by Ta. *J. Phys. Chem. C* **2007**, *111*, 17773–17787. https://doi.org/10.1021/jp070855z.
[b] Irikura, K. K.; Goddard, W. A. Energetics of Third-Row Transition Metal Methylidene IonsMCH_2^+ (M = La, Hf, Ta, W, Re, Os, Ir, Pt, Au). *J. Am. Chem. Soc.* **1994**, *116*, 8733–8740.
[c] Metz, Ricardo B. Photofragment spectroscopy of covalently bound transition metal complexes: a window into C-H and C-C bond activation by transition metal ions *Int. Rev. Phys. Chem.* **2004**, *23*, 79-108.

The value of Irikura and Goddard is from *ab initio* calculations. The lower limit from Metz is from a lower limit on the thermicity of the $Ta^+ + CH_4 \rightarrow TaCH_2^+ + H_2$ reaction of -0.23 ±0.05 eV from photofragment spectroscopy. The value from Parke et al. is from GIB-MS and derived similarly to that here. The reported equilibrium constant from Parke et al. is 107 ±52, a smaller value than found here at 300 K (452 ±95). The derived $\Delta_r H^o$(0K) from Parke et al. (-10 ± 2 kJ mol^{-1}) is correspondingly larger than that found here (-7 ±4), but the derived bond energies happen to be the same due to Parke et al. relying on an experimental determination of the CH_2 enthalpy of formation [D. G. Leopold, K. K. Murray, A. E. S. Miller, and W. C. Lineberger, J. Chem. Phys. 83, 4849 (1985)], which is about 3 kJ mol^{-1} smaller than that favored by the ATcT relying primarily on high level *ab initio* calculations. Interpreting the Parke et al. determination along with the ATcT thermochemistry implies a Ta^+-CH_2 0K BDE 467 ±2 kJ mol^{-1}.

IV. Calculated thermal dissociation rate constants

The forward and reverse reaction rate constants k_a (association) and k_d (dissociation) of

$TaR^+ + H_2$ ←k_d, k_a→ $TaRH_2^+$

are related by the equilibrium constant at a given temperature

$$K_T = \frac{k_d(T)}{n_0 \frac{273.15K}{T} k_a(T)} \qquad (S1)$$

Where n_o is Loschmidt's number 2.69 ×10^{19} molecules cm^{-3}, i.e. the number density of molecules at standard temperature and pressure, accounting for the changing number of moles in the reaction.

K_T may be estimated using from enthalpies and entropies from quantum chemical calculations. k_a, the association rate constant, may be reliably estimated using statistical theory, as described elsewhere, yielding k_d via equation S1. Relevant calculated values are shown in Table S4.

Table S4.

			T(K)			
Species	Level	Quantity	300	400	500	600
$Ta(C_2H_4)^+$ (3)	B3LYP/def2-TZVP	H-E (kJ mol^{-1})	13.7	21.2	29.9	39.5
	B3LYP/def2-TZVP	S (J mol-1 K-1)	302.8	324.2	343.5	361.0
$HHTa(C_2H_2)^+$ (1)	B3LYP/def2-TZVP	H-E (kJ mol^{-1})	13.2	20.7	29.7	39.7
	B3LYP/def2-TZVP	S (J mol-1 K-1)	289.0	310.7	330.7	348.9
$Ta(C_2H_2)^+$ (3)	B3LYP/def2-TZVP	H-E (kJ mol^{-1})	11.4	17.0	23.5	30.6
	B3LYP/def2-TZVP	S (J mol-1 K-1)	288.2	304.3	318.7	331.6
H_2 (1)	B3LYP/def2-TZVP	H-E (kJ mol^{-1})	8.7	11.6	14.5	17.5

	B3LYP/def2-TZVP	S (J mol-1 K-1)	130.5	138.8	145.3	150.6
Reaction						
$Ta(C_2H_4)^+$ (3) → $Ta(C_2H_2)^+$ (3) + H_2 (1)	CCSD(T)/CBS//B3LYP/def2-TZVP	ΔE (kJ mol-1)	62	62	62	62
	CCSD(T)/CBS//B3LYP/def2-TZVP	ΔG (kJ mol-1)	62.0	33.7	21.9	9.9
	CCSD(T)/CBS//B3LYP/def2-TZVP	K	1.5×10^{-6}	1.5×10^{-3}	0.097	1.6
	Statistical theory	k_a (cm^3 s^{-1})	9×10^{-13}	3×10^{-13}	1×10^{-13}	6×10^{-14}
		**k_d(s^{-1})**	**32**	**8200**	**2×10^5**	**1×10^6**
$HHTa(C_2H_2)^+$ (1) → $Ta(C_2H_2)^+$ (3) + H_2 (1)	CCSD(T)/CBS//B3LYP/def2-TZVP	ΔE (kJ mol-1)	117	117	117	117
	CCSD(T)/CBS//B3LYP/def2-TZVP	ΔG (kJ mol-1)	110.9	79.0	65.9	52.6
	CCSD(T)/CBS//B3LYP/def2-TZVP	K	1.5×10^{-15}	4.0×10^{-10}	7.4×10^{-7}	1.1×10^{-4}
	Statistical theory	k_a (cm^3 s^{-1})	2×10^{-11}	7×10^{-12}	3×10^{-12}	1×10^{-12}
		**k_d(s^{-1})**	**8×10^{-7}**	**0.5**	**30**	**1400**

The forward association rate constants k_a were determined from statistical modeling of the reaction, as described elsewhere, for $TaC_2H_2^+ + H_2$ ←→ $TaC_2H_4^+$ using the optimized structures 054, 057, and 319 in the list below.

The dominant source of uncertainty is the reaction enthalpy derived from quantum chemical calculations. The uncertainties in k_d (shaded areas in Figure 4) were estimated by recalculating the equilibrium constant at the with the reaction enthalpies adjusted by ±0.15 eV, assuming that to be the uncertainty in the 0K reaction energies at the CCSD(T)/CBS//B3LYP/def2-TZVP level.

V. Density Functional Calculation Summary

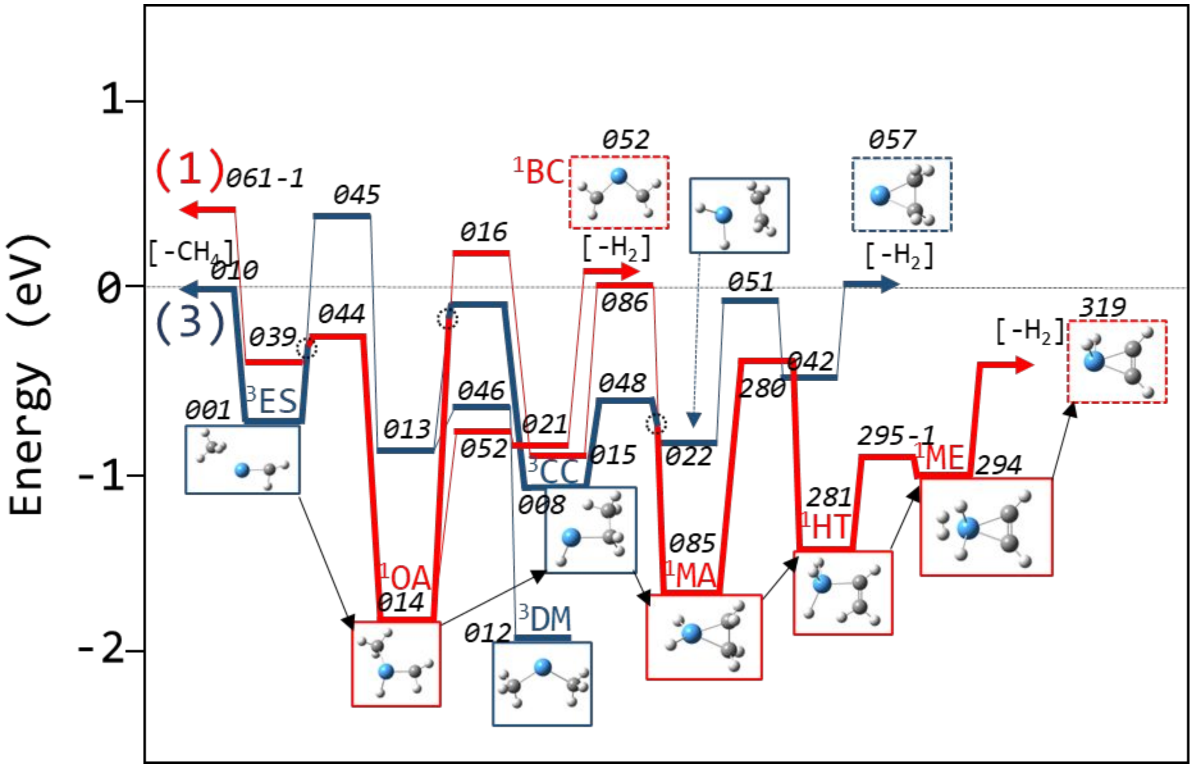

Figure S14. Figure 13 with additional labels corresponding to the structure IDs in Table S3.

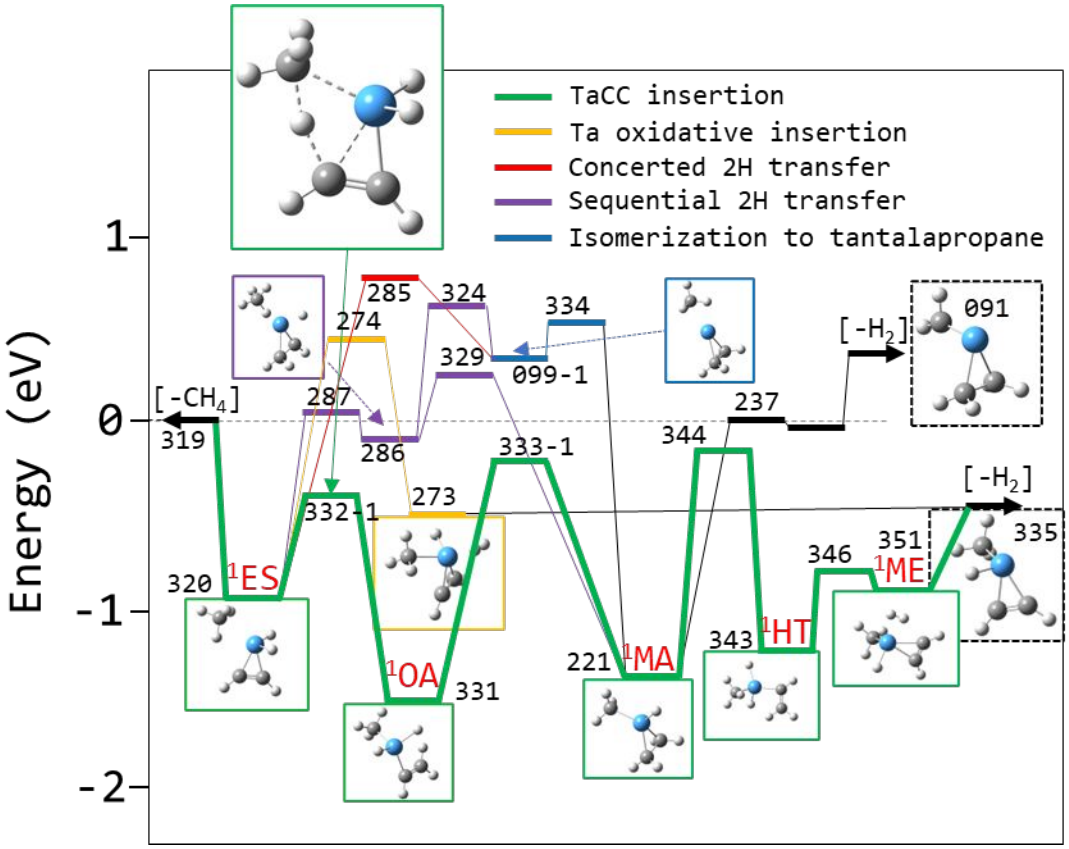

Figure S15. Figure 15 with additional labels corresponding to the structure IDs in Table S3.

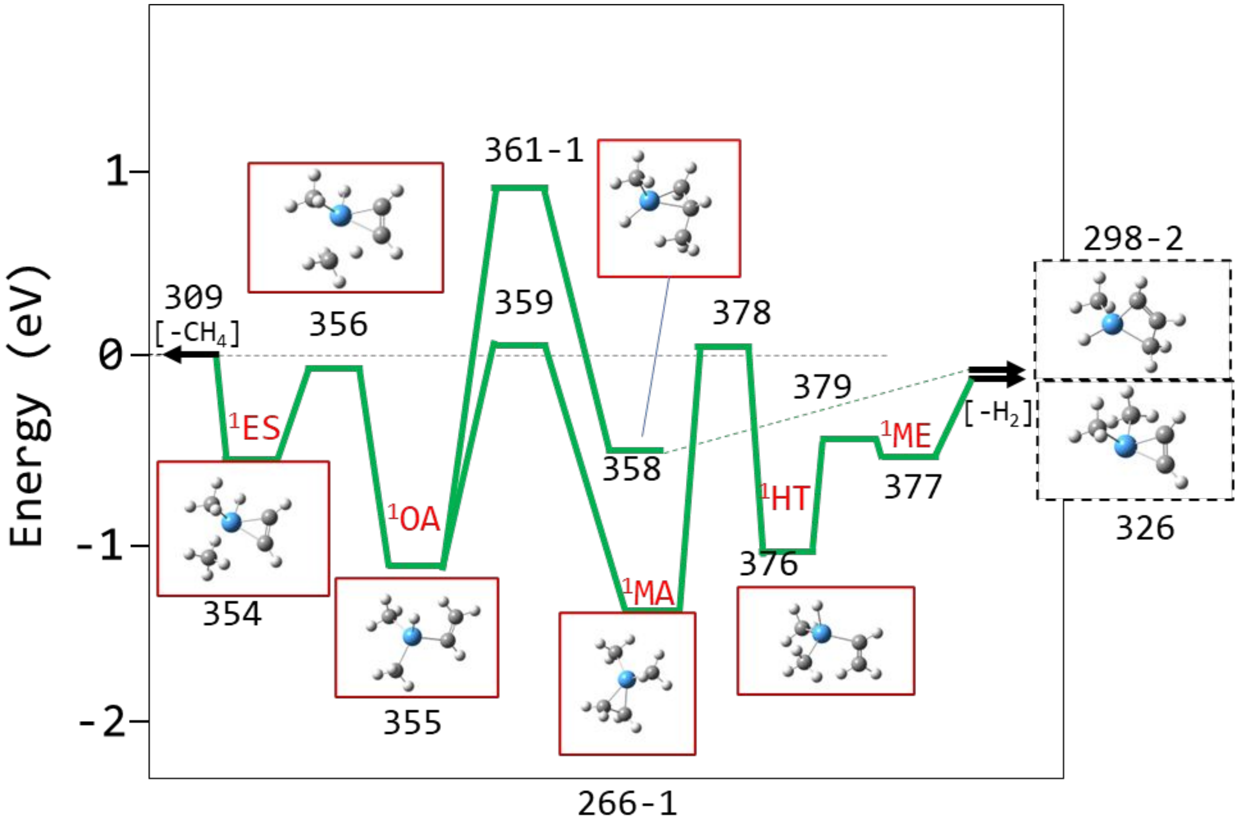

Figure S16. Figure 16 with additional labels corresponding to the structure IDs in Table S3.

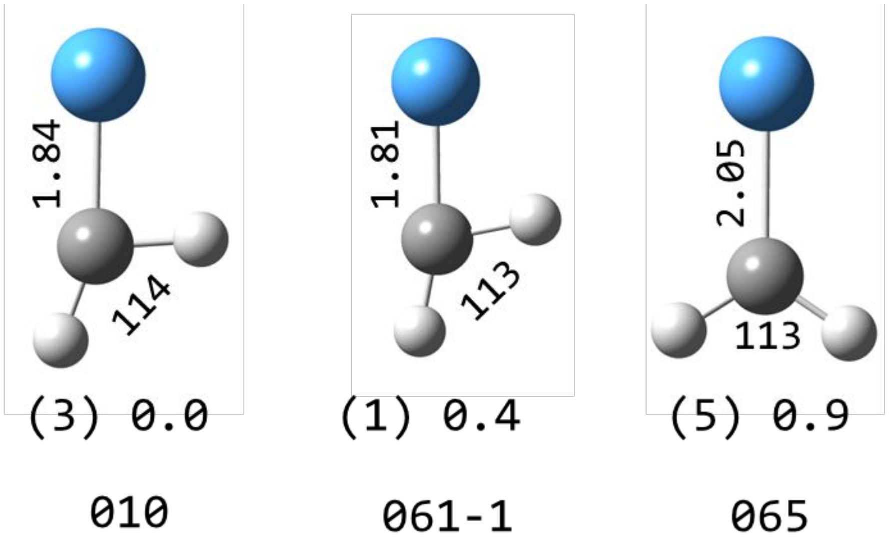

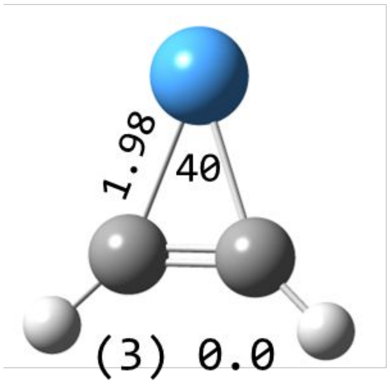

054

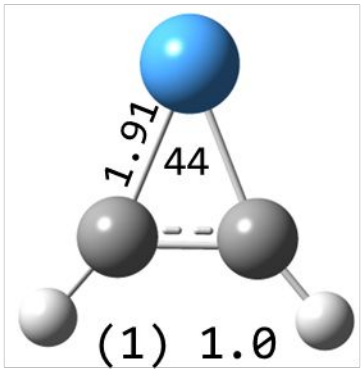

055-3

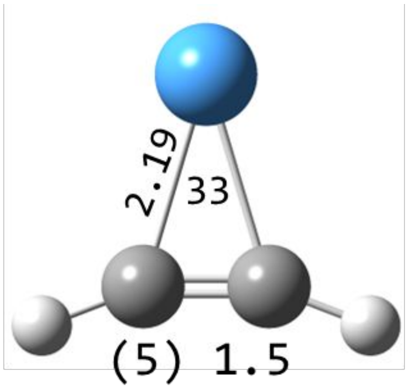

053

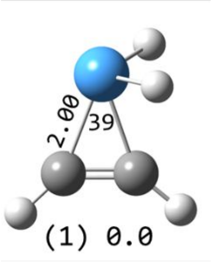

319

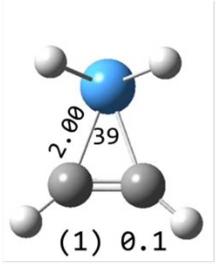

158

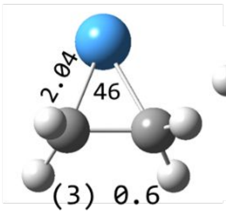

057

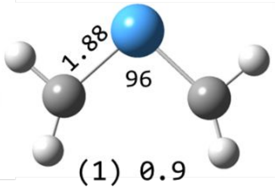

311

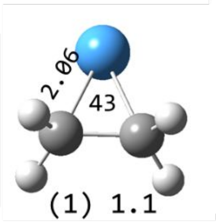

056-1

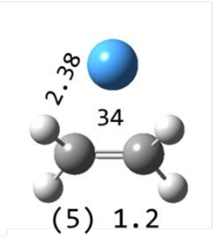

058

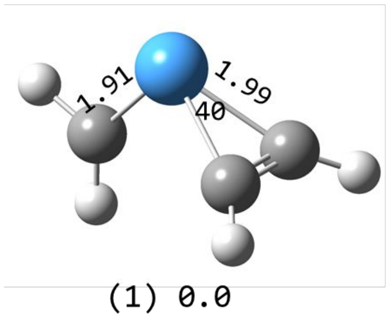

(1) 0.0

102

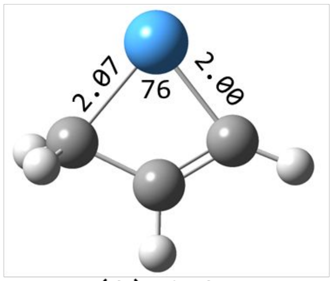

(3) 0.3

300

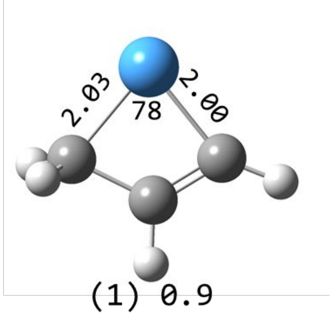

(1) 0.9

219

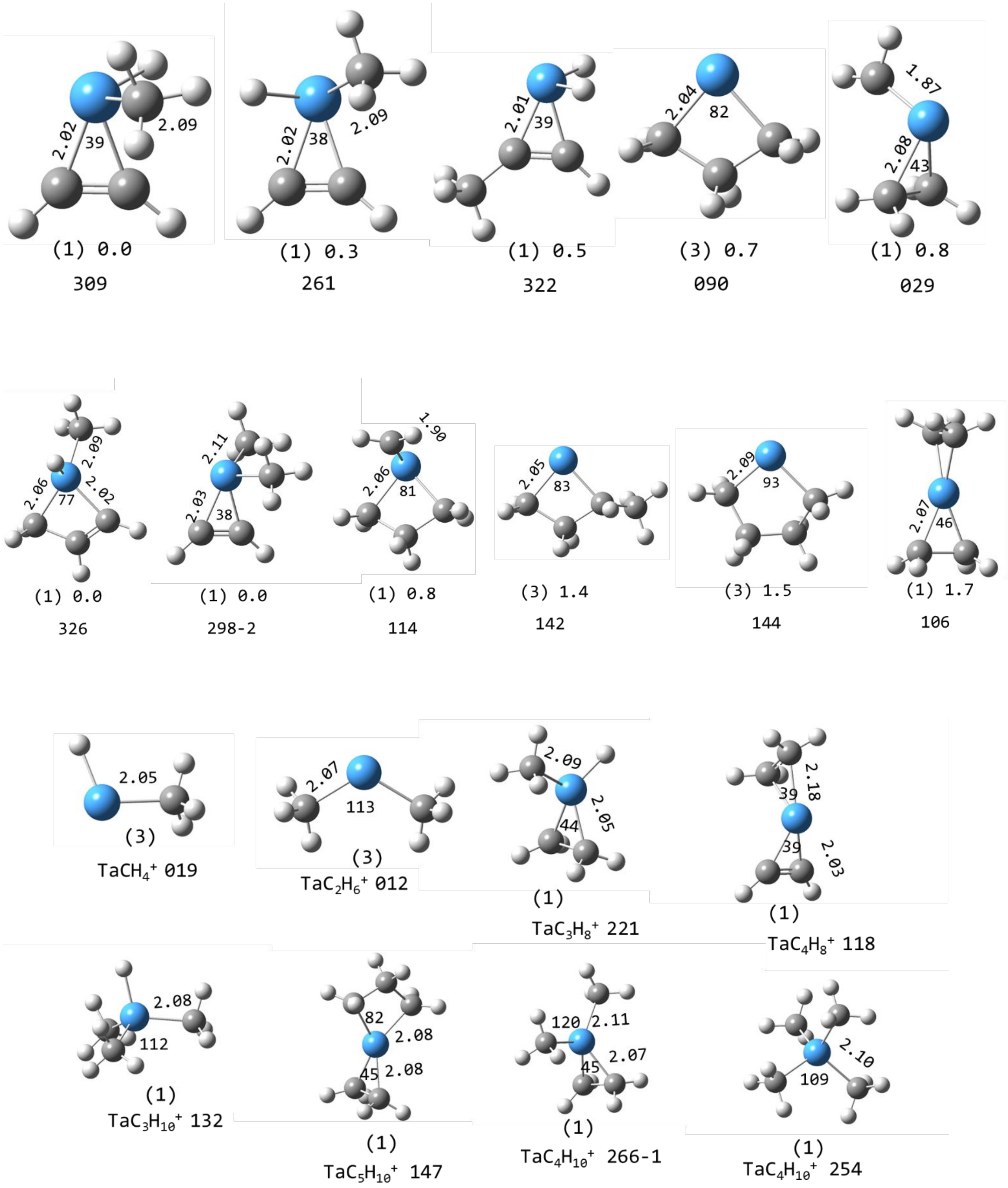

Figure S17. Figures 5 – 11 with labels added corresponding to ID numbers in Table S3 below. Ta-C bond lengths (Å), C-Ta-C bond angles (degrees), and multiplicities are indicated.

Table S3. Summary of stationary points (indicated by section within each molecular formula as minima or transition states) optimized at the B3LYP/def2-TZVP level using the following format:

ID number [for cross-referencing to Figures S14-S17]
Charge Nominal multiplicity (S^2 value [Blank indicates restricted calculation])
Energy [Hartree] Zero-point energy [Hartree]
Structure in cartesian coordinates [X,Y,Z (Å)]
****

Ta^+

Minima

292
Charge:1 Multiplicity:1 ()
E(Hartree):-56.5188567
****
063
Charge:1 Multiplicity:3 (2.0019)
E(Hartree):-56.5742145
****
064
Charge:1 Multiplicity:5 (6.0000)
E(Hartree):-56.5883252
****

TaH^+

Minima

059
Charge:1 Multiplicity:2 (0.7564)
E(Hartree):-57.182713 Zero-point:0.004474
Cartesian coordinates (Angstroms)
Ta 0.000000 ; 0.000000 ; 0.023338
H 0.000000 ; 0.000000 ; -1.703685
****
060
Charge:1 Multiplicity:4 (3.7500)
E(Hartree):-57.195588 Zero-point:0.004415
Cartesian coordinates (Angstroms)
Ta 0.000000 ; 0.000000 ; 0.023489
H 0.000000 ; 0.000000 ; -1.714724

****

TaH_2^+

Minima

250
Charge:1 Multiplicity:3 (2.0000)
E(Hartree):-57.799573 Zero-point:0.010454
Cartesian coordinates (Angstroms)
Ta 0.000000 ; 0.000000 ; 0.026179
H 0.000000 ; 1.429105 ; -0.955534
H 0.000000 ; -1.429105 ; -0.955534
****
251
Charge:1 Multiplicity:5 (6.0000)
E(Hartree):-57.711833 Zero-point:0.006968
Cartesian coordinates (Angstroms)
Ta 0.000000 ; -0.013983 ; 0.000000
H 1.790017 ; 0.510347 ; 0.000000
H -1.790017 ; 0.510391 ; 0.000000
****

$TaCH_2^+$

Minima

061
Charge:1 Multiplicity:1 ()
E(Hartree):-95.919835 Zero-point:0.021292
Cartesian coordinates (Angstroms)
Ta -0.189978 ; -0.004136 ; -0.000009
C 1.625007 ; -0.074509 ; 0.000079
H 1.439910 ; 1.079452 ; 0.000055
H 2.678469 ; -0.330489 ; 0.000146
****
061-1
Charge:1 Multiplicity:1 (0.0227)
E(Hartree):-95.932824 Zero-point:0.021231
Cartesian coordinates (Angstroms)
Ta -0.192469 ; -0.003957 ; -0.000007
C 1.641176 ; -0.064601 ; 0.000051
H 1.531857 ; 1.076345 ; 0.000056
H 2.671309 ; -0.399906 ; 0.000154
****
010
Charge:1 Multiplicity:3 (2.0001)
E(Hartree):-95.934513 Zero-point:0.021077
Cartesian coordinates (Angstroms)
Ta -0.194153 ; -0.003822 ; 0.000001
C 1.652685 ; -0.058590 ; -0.000057
H 1.593046 ; 1.076576 ; 0.000058
H 2.664009 ; -0.446028 ; 0.000184
****
065
Charge:1 Multiplicity:5 (6.0000)
E(Hartree):-95.900906 Zero-point:0.021385
Cartesian coordinates (Angstroms)
Ta -0.000165 ; -0.218004 ; 0.000000
C -0.000165 ; 1.840454 ; 0.000000
H -0.908833 ; 2.447464 ; 0.000000
H 0.921885 ; 2.424099 ; 0.000000
****
017
Charge:1 Multiplicity:1 (0.0000)
E(Hartree):-95.912002 Zero-point:0.018751
Cartesian coordinates (Angstroms)
Ta -0.022507 ; -0.164749 ; 0.000000
C -0.022507 ; 1.597459 ; 0.000000
H 1.720532 ; -0.238579 ; 0.000000
H 0.057485 ; 2.680479 ; 0.000000
****
009
Charge:1 Multiplicity:3 (2.0000)
E(Hartree):-95.890555 Zero-point:0.018668
Cartesian coordinates (Angstroms)
Ta 0.022401 ; -0.173078 ; 0.000000
C 0.022401 ; 1.689344 ; 0.000000
H -1.710759 ; -0.274219 ; 0.000000
H -0.058888 ; 2.772826 ; 0.000000
****
018
Charge:1 Multiplicity:5 (6.0000)
E(Hartree):-95.84989 Zero-point:0.016527
Cartesian coordinates (Angstroms)
Ta -0.021562 ; -0.182332 ; 0.000000
C -0.021562 ; 1.837222 ; 0.000000
H 1.658950 ; -0.636109 ; 0.000000
H 0.044454 ; 2.923030 ; 0.000000
****

Transition States

007
Charge:1 Multiplicity:3 (2.0001)
E(Hartree):-95.933272 Zero-point:0.020379
Cartesian coordinates (Angstroms)
Ta 0.000158 ; -0.200610 ; 0.000000
C 0.000158 ; 1.683554 ; 0.000000
H -0.928490 ; 2.262133 ; 0.000000
H 0.916043 ; 2.281085 ; 0.000000
****
062
Charge:1 Multiplicity:1 (0.0721)
E(Hartree):-95.930399 Zero-point:0.020444
Cartesian coordinates (Angstroms)
Ta -0.000169 ; -0.199751 ; 0.000000
C -0.000169 ; 1.675604 ; 0.000000
H -0.915719 ; 2.274242 ; 0.000000
H 0.929057 ; 2.253940 ; 0.000000
****

$TaCH_3^+$

Minima

066
Charge:1 Multiplicity:2 (0.7548)

E(Hartree):-96.554941   Zero-point:0.032450
Cartesian coordinates (Angstroms)
Ta 0.237376 ; 0.000435 ; 0.000024
C -1.807839 ; 0.002862 ; 0.000122
H -2.186761 ; 0.552389 ; -0.872648
H -2.107796 ; -1.056599 ; -0.056799
H -2.186855 ; 0.455287 ; 0.926941
****
067
Charge:1   Multiplicity:4 (3.7500)
E(Hartree):-96.565157   Zero-point:0.032668
Cartesian coordinates (Angstroms)
Ta 0.000000 ; 0.000000 ; 0.239557
C 0.000000 ; 0.000000 ; -1.824460
H 0.000000 ; 1.039523 ; -2.180304
H 0.900253 ; -0.519762 ; -2.180304
H -0.900253 ; -0.519762 ; -2.180304
****

$TaCH_4^+$

Minima

068
Charge:1   Multiplicity:3 (2.0013)
E(Hartree):-97.132783   Zero-point:0.044669
Cartesian coordinates (Angstroms)
C -2.080929 ; -0.004335 ; 0.013329
H -2.433321 ; -0.097642 ; 1.039535
H -1.585956 ; -0.956424 ; -0.318182
H -2.910796 ; 0.117008 ; -0.686002
H -1.515498 ; 0.967759 ; -0.126270
Ta 0.286728 ; -0.000064 ; 0.000150
****
069
Charge:1   Multiplicity:5 (6.0000)
E(Hartree):-97.14483   Zero-point:0.044947
Cartesian coordinates (Angstroms)
C -2.307180 ; 0.016982 ; 0.007037
H -2.074523 ; 0.866215 ; -0.640360
H -2.481918 ; 0.323055 ; 1.036069
H -3.199322 ; -0.463800 ; -0.398708
H -1.587274 ; -0.846663 ; -0.040564
Ta 0.317618 ; 0.000264 ; 0.000018
****
070-2
Charge:1   Multiplicity:1 ()
E(Hartree):-97.092404   Zero-point:0.044889
Cartesian coordinates (Angstroms)
C -2.096855 ; 0.000008 ; 0.010512
H -1.519968 ; 0.959456 ; -0.126261
H -2.596631 ; 0.001223 ; 0.977208
H -2.816323 ; -0.001021 ; -0.810684
H -1.519953 ; -0.959725 ; -0.124117
Ta 0.288137 ; 0.000000 ; 0.000285
****
071
Charge:1   Multiplicity:3 (2.0000)
E(Hartree):-97.140686   Zero-point:0.037444
Cartesian coordinates (Angstroms)
Ta -0.207057 ; -0.042206 ; -0.000017
C 1.710492 ; -0.062621 ; -0.000078
H 2.302021 ; -0.082635 ; 0.919124
H 2.303273 ; -0.080486 ; -0.918480
H -0.285616 ; 1.891323 ; 0.000451
H 0.532528 ; 1.728583 ; 0.000631
****
072
Charge:1   Multiplicity:5 (6.0000)
E(Hartree):-97.10067   Zero-point:0.036242
Cartesian coordinates (Angstroms)
Ta -0.188772 ; -0.066085 ; -0.000021
C 1.869110 ; 0.178525 ; -0.000004
H 2.385845 ; 1.138093 ; -0.001163
H 2.559813 ; -0.671048 ; 0.001433
H -1.189499 ; 1.643111 ; 0.399103
H -1.190472 ; 1.642928 ; -0.397816
****
291-1
Charge:1   Multiplicity:1 ()
E(Hartree):-97.149596   Zero-point:0.038168
Cartesian coordinates (Angstroms)
C -1.787271 ; 0.068815 ; -0.000049
H -2.001030 ; -0.524835 ; -0.916167
H -2.375450 ; 0.987294 ; -0.000007
H -2.001078 ; -0.524947 ; 0.915986
H 0.899239 ; 1.565507 ; -0.000025
Ta 0.221944 ; -0.026245 ; 0.000007
****
019
Charge:1   Multiplicity:3 (2.0000)
E(Hartree):-97.178049   Zero-point:0.038714
Cartesian coordinates (Angstroms)
C 1.823865 ; 0.044979 ; -0.000047
H 2.199980 ; -0.463130 ; 0.901403
H 2.153282 ; 1.092807 ; -0.000057
H 2.199928 ; -0.463129 ; -0.901520
H -0.788878 ; 1.620497 ; -0.000215
Ta -0.228870 ; -0.028177 ; 0.000009
****
020
Charge:1   Multiplicity:1 (0.0318)
E(Hartree):-97.131859   Zero-point:0.037565
Cartesian coordinates (Angstroms)
Ta -0.206406 ; -0.042623 ; -0.000004
C 1.707183 ; -0.061534 ; -0.000040
H 2.297749 ; -0.079002 ; 0.920016
H 2.298570 ; -0.078877 ; -0.919535
H -0.294603 ; 1.897965 ; 0.000053
H 0.522834 ; 1.740599 ; 0.000014
****

Transition states

073
Charge:1   Multiplicity:3 (2.0000)
E(Hartree):-97.139277   Zero-point:0.035944
Cartesian coordinates (Angstroms)
C 1.719442 ; -0.108940 ; 0.000016
H 2.313686 ; -0.140876 ; 0.916967
H 0.903707 ; 1.424940 ; -0.000159
H 2.313566 ; -0.141088 ; -0.917014

H -0.003744 ; 1.792467 ; 0.000022
Ta -0.217039 ; -0.031258 ; 0.000001
****

074
Charge:1 Multiplicity:3 (2.0000)
E(Hartree):-97.124096 Zero-point:0.038292
Cartesian coordinates (Angstroms)
C -1.814644 ; -0.013747 ; 0.000047
H -1.357049 ; -1.135186 ; -0.000290
H -2.427421 ; 0.063274 ; -0.901614
H -2.428768 ; 0.062281 ; 0.900858
H -1.111141 ; 1.218397 ; 0.000507
Ta 0.249483 ; -0.001730 ; 0.000003
****

290-1
Charge:1 Multiplicity:1 (0.0000)
E(Hartree):-97.098914 Zero-point:0.035746
Cartesian coordinates (Angstroms)
C 1.710908 ; 0.000016 ; 0.000040
H 0.964466 ; -1.381783 ; 0.000039
H 2.293362 ; -0.000052 ; 0.925081
H 2.292508 ; -0.000171 ; -0.925576
H 0.964760 ; 1.381762 ; -0.000041
Ta -0.229870 ; 0.000002 ; 0.000004
****

075
Charge:1 Multiplicity:5 (6.0000)
E(Hartree):-97.062346 Zero-point:0.034523
Cartesian coordinates (Angstroms)
Ta -0.248791 ; -0.358759 ; 0.204668
C -2.217669 ; 0.108515 ; -0.618962
H -2.807343 ; -0.662600 ; -1.114851
H -2.567626 ; 1.127110 ; -0.787393
H -1.429716 ; -0.247340 ; 1.613278
H -2.061454 ; -0.070872 ; 0.666752
****

076
Charge:1 Multiplicity:5 (6.0000)
E(Hartree):-97.086193 Zero-point:0.037133
Cartesian coordinates (Angstroms)
C 2.271743 ; -0.020036 ; -0.000004
H 2.392690 ; -1.099895 ; 0.001449
H 2.424301 ; 0.519051 ; 0.928899
H 2.423820 ; 0.516499 ; -0.930475
H -0.024785 ; 1.715089 ; 0.000023
Ta -0.285568 ; -0.020966 ; 0.000002
****

$TaC_2H_2^+$

Minima

054
Charge:1 Multiplicity:3 (2.0000)
E(Hartree):-134.080999 Zero-point:0.028837
Cartesian coordinates (Angstroms)
Ta 0.000000 ; 0.317104 ; 0.000000
C 0.673559 ; -1.546482 ; 0.000000
H 1.458382 ; -2.292928 ; 0.000000
C -0.673803 ; -1.546920 ; 0.000000
H -1.456920 ; -2.295245 ; 0.000000
****

053
Charge:1 Multiplicity:5 (6.0000)
E(Hartree):-134.022125 Zero-point:0.027230
Cartesian coordinates (Angstroms)
Ta 0.000000 ; 0.347208 ; 0.000000
C 0.626053 ; -1.754937 ; 0.000000
H 1.633356 ; -2.142634 ; 0.000000
C -0.626061 ; -1.755151 ; 0.000000
H -1.633311 ; -2.143034 ; 0.000000
****

055
Charge:1 Multiplicity:1 (0.0198)
E(Hartree):-134.074596 Zero-point:0.028885
Cartesian coordinates (Angstroms)
Ta 0.000000 ; 0.317723 ; 0.000000
C 0.670448 ; -1.552257 ; 0.000000
H 1.469644 ; -2.283333 ; 0.000000
C -0.670433 ; -1.552273 ; 0.000000
H -1.469737 ; -2.283233 ; 0.000000
****

055-3
Charge:1 Multiplicity:1 ()
E(Hartree):-134.042048 Zero-point:0.027697
Cartesian coordinates (Angstroms)
Ta 0.000000 ; 0.304036 ; 0.000000
C -0.715095 ; -1.467223 ; 0.000000
H -1.413232 ; -2.293977 ; 0.000000
C 0.715096 ; -1.467223 ; 0.000000
H 1.413223 ; -2.293986 ; 0.000000
****

165
Charge:1 Multiplicity:1 (0.0000)
E(Hartree):-133.963206 Zero-point:0.021343
Cartesian coordinates (Angstroms)
Ta -0.253721 ; 0.000022 ; -0.043254
C 1.628255 ; 0.636823 ; 0.031393
C 1.628018 ; -0.636976 ; 0.031254
H -0.507654 ; 0.902686 ; 1.391102
H -0.508374 ; -0.903352 ; 1.390560
****

288
Charge:1 Multiplicity:3 (2.0002)
E(Hartree):-133.953707 Zero-point:0.024599
Cartesian coordinates (Angstroms)
Ta 0.000000 ; 0.221983 ; 0.000000
C 1.393283 ; -0.924675 ; 0.000000
C -1.423601 ; -1.142799 ; 0.000000
H 2.140697 ; -1.711733 ; 0.000000
H -1.958793 ; -2.088196 ; 0.000000
****

289
Charge:1 Multiplicity:1 (0.0000)
E(Hartree):-134.042048 Zero-point:0.027709
Cartesian coordinates (Angstroms)
Ta 0.000000 ; 0.304089 ; 0.000000
C 0.714556 ; -1.467651 ; 0.000000
C -0.714508 ; -1.467548 ; 0.000000
H 1.413364 ; -2.293848 ; 0.000000
H -1.413651 ; -2.293467 ; 0.000000
****

$TaC_2H_3^+$

Minima

250
Charge:1 Multiplicity:1 (0.0000)
E(Hartree):-172.170645 Zero-point:0.033817
Cartesian coordinates (Angstroms)
C 1.152813 ; -1.248315 ; 0.000295
H 1.571829 ; -2.244196 ; -0.001360
C 1.688856 ; -0.000050 ; 0.000034
C 1.153011 ; 1.248242 ; 0.000091
H 1.571690 ; 2.244254 ; -0.000679
Ta -0.371392 ; 0.000009 ; -0.000007
****
251
Charge:1 Multiplicity:1 (0.0000)
E(Hartree):-172.108576 Zero-point:0.031199
Cartesian coordinates (Angstroms)
Ta 0.386303 ; 0.000016 ; 0.019416
C -1.414992 ; 0.759106 ; 0.307793
H -2.184592 ; 1.477301 ; 0.547542
C -1.415615 ; -0.757506 ; 0.310011
H -2.185045 ; -1.474774 ; 0.553006
C -1.141138 ; -0.002214 ; -1.037453
****

$TaC_2H_4^+$

Minima

057
Charge:1 Multiplicity:3 (2.0000)
E(Hartree):-135.284293 Zero-point:0.048983
Cartesian coordinates (Angstroms)
Ta -0.355820 ; 0.000002 ; 0.000031
C 1.521483 ; -0.794773 ; 0.000067
H 1.930688 ; -1.241115 ; 0.907340
H 1.927711 ; -1.240771 ; -0.908854
C 1.521512 ; 0.794764 ; 0.000068
H 1.927763 ; 1.240729 ; -0.908857
H 1.930754 ; 1.241077 ; 0.907338
****
058
Charge:1 Multiplicity:5 (6.0000)
E(Hartree):-135.272764 Zero-point:0.051999
Cartesian coordinates (Angstroms)
Ta -0.414562 ; 0.000008 ; 0.000002
C 1.860848 ; 0.688197 ; 0.000050
H 1.983407 ; 1.248842 ; -0.922763
H 1.983745 ; 1.249424 ; 0.922456
C 1.860732 ; -0.688264 ; 0.000026
H 1.983298 ; -1.249600 ; 0.922418
H 1.983105 ; -1.248876 ; -0.922749
****
056
Charge:1 Multiplicity:1 (0.0394)
E(Hartree):-135.286873 Zero-point:0.050332
Cartesian coordinates (Angstroms)
Ta -0.362569 ; 0.000000 ; -0.000060
C 1.576566 ; 0.749534 ; 0.000247
H 1.887353 ; 1.263662 ; -0.908310
H 1.887030 ; 1.263706 ; 0.908891
C 1.576563 ; -0.749534 ; 0.000266
H 1.886978 ; -1.263669 ; 0.908949
H 1.887394 ; -1.263708 ; -0.908249
****
056-1
Charge:1 Multiplicity:1 ()
E(Hartree):-135.274477 Zero-point:0.049930
Cartesian coordinates (Angstroms)
Ta 0.358331 ; 0.000000 ; -0.000062
C -1.560544 ; -0.748041 ; 0.000257
H -1.858066 ; -1.267971 ; -0.910382
H -1.857743 ; -1.268006 ; 0.910981
C -1.560544 ; 0.748041 ; 0.000280
H -1.857713 ; 1.267965 ; 0.911039
H -1.858097 ; 1.268012 ; -0.910323
****
005
Charge:1 Multiplicity:3 (2.0000)
E(Hartree):-135.256533 Zero-point:0.044318
Cartesian coordinates (Angstroms)
Ta 0.000058 ; -0.260845 ; -0.012042
C -1.411962 ; 1.091028 ; 0.034284
H -2.160866 ; 1.005896 ; 0.831813
H -1.545624 ; 1.966596 ; -0.597928
C 1.411496 ; 1.091541 ; 0.034289
H 1.544475 ; 1.967177 ; -0.597946
H 2.160576 ; 1.006576 ; 0.831667
****
026
Charge:1 Multiplicity:1 (0.3545)
E(Hartree):-135.253764 Zero-point:0.043798
Cartesian coordinates (Angstroms)
Ta -0.000044 ; -0.255551 ; -0.012591
C -1.435966 ; 1.070536 ; 0.031264
H -2.148226 ; 0.961154 ; 0.860704
H -1.616489 ; 1.945468 ; -0.589131
C 1.436393 ; 1.069969 ; 0.031295
H 1.618496 ; 1.943764 ; -0.590224
H 2.146890 ; 0.961833 ; 0.862432
****
311
Charge:1 Multiplicity:1 ()
E(Hartree):-135.277085 Zero-point:0.044654
Cartesian coordinates (Angstroms)
Ta 0.000071 ; -0.233452 ; 0.000001
C -1.392616 ; 1.025460 ; 0.000007
H -2.241967 ; 0.299804 ; -0.000087
H -1.684804 ; 2.066728 ; 0.000026
C 1.391871 ; 1.026130 ; -0.000003
H 1.685878 ; 2.066876 ; -0.000016
H 2.240204 ; 0.299023 ; -0.000019
****
079
Charge:1 Multiplicity:3 (2.0001)
E(Hartree):-135.27171 Zero-point:0.050677
Cartesian coordinates (Angstroms)
Ta 0.397415 ; -0.025496 ; 0.000000
C -1.267841 ; 0.834530 ; 0.000012
H -1.750616 ; 1.805254 ; -0.000158
H -2.732521 ; -0.496223 ; -0.897572
C -2.113655 ; -0.431514 ; 0.000018
H -1.507063 ; -1.369699 ; -0.000290

H -2.732113 ; -0.496210 ; 0.897850
****
158
Charge:1 Multiplicity:1 (0.0000)
E(Hartree):-135.299528 Zero-point:0.043809
Cartesian coordinates (Angstroms)
Ta -0.309285 ; -0.000016 ; -0.038082
C 1.570941 ; 0.667975 ; 0.003693
H 2.288863 ; 1.477288 ; 0.043119
C 1.570928 ; -0.667905 ; 0.003774
H 2.288974 ; -1.477118 ; 0.043068
H -0.426120 ; 1.055207 ; 1.324228
H -0.425115 ; -1.054603 ; 1.324801
****
159
Charge:1 Multiplicity:3 (2.0000)
E(Hartree):-135.271175 Zero-point:0.042153
Cartesian coordinates (Angstroms)
Ta -0.265395 ; -0.001012 ; -0.055409
C 1.603808 ; 0.673144 ; 0.126785
H 2.345194 ; 1.457715 ; 0.213636
C 1.605627 ; -0.668385 ; 0.135167
H 2.349996 ; -1.448971 ; 0.231676
H -2.273105 ; 0.398654 ; 1.031119
H -2.304855 ; -0.362074 ; 0.996720
****
243
Charge:1 Multiplicity:1 (0.0000)
E(Hartree):-135.281922 Zero-point:0.044844
Cartesian coordinates (Angstroms)
Ta -0.311227 ; -0.016842 ; -0.025358
C 1.270183 ; 0.914921 ; -0.037025
H 2.051878 ; 1.660859 ; 0.032338
H -0.569929 ; 0.185452 ; 1.680826
C 1.583891 ; -0.670988 ; 0.036912
H 2.035584 ; -0.985683 ; 0.981255
H 2.077564 ; -1.094773 ; -0.842604
****
319
Charge:1 Multiplicity:1 ()
E(Hartree):-135.307593 Zero-point:0.043616
Cartesian coordinates (Angstroms)
Ta -0.307654 ; -0.030020 ; -0.000008
C 1.596940 ; -0.648717 ; -0.000123
H 2.379656 ; -1.398449 ; -0.000188
C 1.548777 ; 0.686787 ; 0.000076
H 2.216013 ; 1.538400 ; 0.000412
H -0.505683 ; 0.912345 ; -1.452416
H -0.505569 ; 0.910747 ; 1.453089
****

Transition states

312
Charge:1 Multiplicity:1 (0.0000)
E(Hartree):-135.225251 Zero-point:0.044096
Cartesian coordinates (Angstroms)
Ta -0.124567 ; -0.250827 ; -0.008657
C 1.713618 ; 0.458815 ; 0.020195
H 2.362306 ; 0.310793 ; 0.894202
H 2.160537 ; 1.091876 ; -0.742610
C -0.672829 ; 1.630140 ; 0.017899
H -0.529423 ; 2.431900 ; -0.704999
H -1.144725 ; 1.942097 ; 0.956819
****
006
Charge:1 Multiplicity:3 (2.0000)
E(Hartree):-135.251635 Zero-point:0.045548
Cartesian coordinates (Angstroms)
Ta -0.303516 ; -0.000002 ; -0.007548
C 1.265438 ; 1.194451 ; 0.017831
H 1.423757 ; 1.842182 ; 0.887211
H 2.062273 ; 1.260446 ; -0.719299
C 1.265350 ; -1.194507 ; 0.017935
H 2.061466 ; -1.261518 ; -0.719891
H 1.424457 ; -1.840614 ; 0.888384
****
077
Charge:1 Multiplicity:3 (2.0000)
E(Hartree):-135.261169 Zero-point:0.039907
Cartesian coordinates (Angstroms)
Ta -0.406504 ; -0.092286 ; 0.003481
C 1.054607 ; 1.246112 ; 0.003836
H 1.443188 ; 2.256864 ; 0.029032
C 1.571940 ; 0.003787 ; -0.062240
H 2.562819 ; -0.432046 ; -0.113578
H 4.925129 ; -1.470631 ; -0.235984
H 4.984337 ; -1.116700 ; 0.416805
****
078
Charge:1 Multiplicity:3 (2.0001)
E(Hartree):-135.197292 Zero-point:0.044041
Cartesian coordinates (Angstroms)
Ta -0.365577 ; -0.022991 ; 0.001582
C 1.293730 ; 0.912169 ; 0.049571
H 1.875452 ; 1.824647 ; 0.075180
H 2.630275 ; -0.817710 ; 0.821339
C 1.695086 ; -0.444308 ; -0.191141
H 1.928355 ; -1.298534 ; 0.823515
H 2.320167 ; -0.837233 ; -0.986110
****
080
Charge:1 Multiplicity:3 (2.0003)
E(Hartree):-135.218467 Zero-point:0.046579
Cartesian coordinates (Angstroms)
Ta -0.360203 ; -0.015606 ; -0.001136
C 1.320685 ; 0.906165 ; 0.084738
H 1.809467 ; 1.728831 ; -0.434695
H 2.385140 ; 0.307041 ; 0.433347
C 1.683343 ; -0.674226 ; -0.050417
H 1.932510 ; -1.247140 ; 0.845443
H 2.143500 ; -1.041129 ; -0.967101
****
241
Charge:1 Multiplicity:1 (0.0000)
E(Hartree):-135.25102 Zero-point:0.045173
Cartesian coordinates (Angstroms)
Ta -0.375137 ; 0.180532 ; -0.647367
C 1.254231 ; 0.685801 ; 0.155268
H 1.993568 ; 1.381900 ; 0.533954
H -0.031229 ; 1.130219 ; 0.825418
C 1.079466 ; -0.800317 ; 0.439945
H 0.937629 ; -1.060251 ; 1.491697

H 1.700658 ; -1.517056 ; -0.094313
****
245
Charge:1 Multiplicity:1 (0.0000)
E(Hartree):-135.268985 Zero-point:0.042387
Cartesian coordinates (Angstroms)
Ta -0.340440 ; 0.215716 ; -0.247043
C 1.492197 ; 0.687179 ; -0.006611
H 2.331057 ; 1.330469 ; 0.222634
H -0.648939 ; 0.967852 ; 1.284850
C 1.309365 ; -0.732508 ; 0.166070
H 0.388239 ; -0.927802 ; 1.056661
H 2.027705 ; -1.540077 ; 0.228041
****
*

$TaC_2H_5^+$
Minima
082
Charge:1 Multiplicity:2 (0.7552)
E(Hartree):-135.888322 Zero-point:0.061878
Cartesian coordinates (Angstroms)
Ta 0.419640 ; -0.020291 ; -0.000010
C -1.463707 ; 0.841663 ; -0.000053
H -1.672180 ; 1.430951 ; 0.895491
H -1.673945 ; 1.431968 ; -0.894394
C -2.022715 ; -0.571651 ; -0.000075
H -2.582762 ; -0.817643 ; -0.899839
H -2.585513 ; -0.816780 ; 0.898266
H -1.200785 ; -1.367302 ; 0.001995
****
081
Charge:1 Multiplicity:4 (3.7500)
E(Hartree):-135.898027 Zero-point:0.061926
Cartesian coordinates (Angstroms)
Ta 0.420736 ; -0.020142 ; -0.000011
C -1.468828 ; 0.843315 ; -0.000072
H -1.681522 ; 1.429021 ; 0.896843
H -1.683586 ; 1.430185 ; -0.895595
C -2.025970 ; -0.573497 ; -0.000050
H -2.587971 ; -0.820691 ; -0.898288
H -2.590771 ; -0.819893 ; 0.896688
H -1.201111 ; -1.367164 ; 0.001899
****

$TaC_2H_6^+$

Minima
001
Charge:1 Multiplicity:3 (2.0001)
E(Hartree):-136.49587 Zero-point:0.067506
Cartesian coordinates (Angstroms)
Ta 0.000158 ; -0.200610 ; 0.000000
C 0.000158 ; 1.683554 ; 0.000000
H -0.928490 ; 2.262133 ; 0.000000
H 0.916043 ; 2.281085 ; 0.000000
C 0.192598 ; -4.083716 ; 0.029271
H 0.549253 ; -5.092526 ; 0.029271
H 0.549271 ; -3.579318 ; 0.902923
H 0.549271 ; -3.579318 ; -0.844380
H -0.877402 ; -4.083703 ; 0.029271
****
002
Charge:1 Multiplicity:5 (6.0000)
E(Hartree):-136.463499 Zero-point:0.067414
Cartesian coordinates (Angstroms)
Ta 0.080169 ; -0.103448 ; -0.000027
C 2.076646 ; 0.475579 ; 0.000129
H 2.482529 ; 1.486136 ; -0.000380
H 2.870510 ; -0.279855 ; 0.000646
C -2.351707 ; 0.336564 ; 0.000106
H -2.265897 ; -0.266577 ; 0.916381
H -1.675786 ; 1.226059 ; -0.001401
H -2.267104 ; -0.268497 ; -0.915012
H -3.346252 ; 0.781595 ; 0.000362
****
039
Charge:1 Multiplicity:1 (0.0146)
E(Hartree):-136.494012 Zero-point:0.067761
Cartesian coordinates (Angstroms)
Ta 0.115166 ; -0.114944 ; 0.000009
C 1.789630 ; 0.653752 ; -0.000010
H 2.090882 ; -0.464181 ; -0.000381
H 2.641155 ; 1.321132 ; 0.000048
C -2.373109 ; 0.346663 ; -0.000043
H -3.290205 ; 0.936000 ; 0.000297
H -1.859552 ; 0.655833 ; -0.932887
H -1.859440 ; 0.655068 ; 0.933008
H -2.629088 ; -0.715456 ; -0.000451
****
004
Charge:1 Multiplicity:3 (2.0000)
E(Hartree):-136.456174 Zero-point:0.060708
Cartesian coordinates (Angstroms)
Ta -0.022740 ; -0.216220 ; -0.051234
C 1.774731 ; 0.766354 ; 0.143805
H 2.392067 ; 0.919018 ; -0.750145
H 2.204437 ; 1.225701 ; 1.031961
C -1.316591 ; 1.219591 ; -0.022929
H -1.359156 ; 2.194344 ; 0.457329
H -2.194705 ; 1.013589 ; -0.656348
H -0.828789 ; -1.080551 ; 1.524932
H -1.302677 ; -0.403743 ; 1.407120
****
041
Charge:1 Multiplicity:3 (2.0017)
E(Hartree):-136.470839 Zero-point:0.073779
Cartesian coordinates (Angstroms)
C -1.903378 ; 0.758803 ; 0.023219
H -2.642779 ; 1.160607 ; -0.670589
H -0.986344 ; 1.319885 ; -0.389226
H -2.078211 ; 1.164605 ; 1.018592
C -1.903528 ; -0.758794 ; -0.023248
H -0.986651 ; -1.319658 ; 0.389308
H -2.642951 ; -1.160417 ; 0.670656
H -2.078224 ; -1.164506 ; -1.018637
Ta 0.469268 ; -0.000008 ; 0.000001
****
040
Charge:1 Multiplicity:1 (3.1264)
E(Hartree):-136.463409 Zero-point:0.073778
Cartesian coordinates (Angstroms)

C	-1.922234 ; 0.754715 ; 0.022928
H	-2.670321 ; 1.143771 ; -0.669176
H	-1.012971 ; 1.324429 ; -0.385299
H	-2.103891 ; 1.154961 ; 1.019196
C	-1.896001 ; -0.763667 ; -0.024405
H	-0.982789 ; -1.307198 ; 0.419750
H	-2.645665 ; -1.178247 ; 0.651401
H	-2.045764 ; -1.172673 ; -1.022550
Ta	0.470833 ; 0.001215 ; -0.000061
****
033
Charge:1 Multiplicity:1 (0.0102)
E(Hartree):-136.537512 Zero-point:0.066737
Cartesian coordinates (Angstroms)
Ta	0.000028 ; -0.231048 ; 0.000026
C	-1.733713 ; 0.888186 ; 0.000355
H	-1.642852 ; 1.975912 ; -0.002480
H	-2.284733 ; 0.562160 ; -0.899486
H	-2.289144 ; 0.566338 ; 0.898504
C	1.733241 ; 0.888721 ; 0.000248
H	2.287850 ; 0.560334 ; 0.896852
H	2.282573 ; 0.563651 ; -0.901018
H	1.647111 ; 1.976693 ; 0.002144
****
033-1
Charge:1 Multiplicity:1 ()
E(Hartree):-136.52279 Zero-point:0.065915
Cartesian coordinates (Angstroms)
Ta	-0.000003 ; 0.227953 ; -0.000002
C	-1.696631 ; -0.890802 ; -0.000040
H	-1.722211 ; -1.979227 ; 0.000551
H	-2.194267 ; -0.497725 ; 0.910428
H	-2.194720 ; -0.498583 ; -0.910559
C	1.696658 ; -0.890781 ; -0.000014
H	2.194615 ; -0.498125 ; -0.910443
H	2.194479 ; -0.498205 ; 0.910530
H	1.722183 ; -1.979208 ; -0.000051
****
012
Charge:1 Multiplicity:3 (2.0000)
E(Hartree):-136.542542 Zero-point:0.067041
Cartesian coordinates (Angstroms)
Ta	0.000044 ; -0.238311 ; 0.000004
C	-1.730274 ; 0.909728 ; 0.000132
H	-1.573743 ; 1.991107 ; -0.000883
H	-2.304033 ; 0.623377 ; -0.896433
H	-2.305378 ; 0.624960 ; 0.896136
C	1.729837 ; 0.910037 ; 0.000074
H	2.304099 ; 0.622586 ; 0.895882
H	2.302822 ; 0.624347 ; -0.897116
H	1.575632 ; 1.991730 ; 0.000910
****
042
Charge:1 Multiplicity:3 (2.0000)
E(Hartree):-136.486542 Zero-point:0.065308
Cartesian coordinates (Angstroms)
Ta	0.326327 ; -0.000077 ; -0.065121
C	-1.623967 ; -0.751615 ; 0.124155
H	-1.994688 ; -1.238213 ; -0.779875
H	-1.911879 ; -1.269535 ; 1.034835
C	-1.623979 ; 0.751909 ; 0.123264
H	-1.910786 ; 1.270463 ; 1.033964
H	-1.995776 ; 1.237833 ; -0.780630
H	1.739440 ; 0.397855 ; 1.380078
H	1.739481 ; -0.394551 ; 1.380952
****
043
Charge:1 Multiplicity:1 (0.0368)
E(Hartree):-136.470591 Zero-point:0.063625
Cartesian coordinates (Angstroms)
Ta	0.301719 ; 0.000082 ; -0.000046
C	-1.599622 ; -0.778764 ; 0.000089
H	-1.982693 ; -1.243168 ; -0.908497
H	-1.982379 ; -1.242889 ; 0.908966
C	-1.600035 ; 0.778354 ; 0.000033
H	-1.983032 ; 1.242007 ; 0.908999
H	-1.983655 ; 1.242478 ; -0.908446
H	2.553109 ; -0.001396 ; -0.382763
H	2.551124 ; -0.000528 ; 0.384375
****
043-1
Charge:1 Multiplicity:1 (0.0000)
E(Hartree):-136.463629 Zero-point:0.064024
Cartesian coordinates (Angstroms)
Ta	0.307608 ; 0.000038 ; -0.022751
C	-1.653346 ; -0.735508 ; 0.054791
H	-1.953567 ; -1.263412 ; -0.850581
H	-1.912679 ; -1.271036 ; 0.966540
C	-1.653495 ; 0.735303 ; 0.054844
H	-1.912952 ; 1.270688 ; 0.966639
H	-1.953858 ; 1.263225 ; -0.850467
H	2.559477 ; 0.380199 ; 0.385710
H	2.559278 ; -0.381226 ; 0.385171
****
013
Charge:1 Multiplicity:3 (2.0000)
E(Hartree):-136.499176 Zero-point:0.061955
Cartesian coordinates (Angstroms)
Ta	-0.026368 ; -0.205264 ; -0.033581
C	-1.723421 ; 0.974962 ; 0.072732
H	-2.682869 ; 0.584450 ; 0.422646
H	-1.763534 ; 2.035639 ; -0.181220
C	1.753739 ; 0.851835 ; 0.096857
H	2.164771 ; 0.860246 ; -0.929819
H	1.640895 ; 1.888675 ; 0.431512
H	-0.073457 ; -1.669879 ; 0.935370
H	2.457134 ; 0.324383 ; 0.755385
****
014
Charge:1 Multiplicity:1 (0.0000)
E(Hartree):-136.532297 Zero-point:0.062791
Cartesian coordinates (Angstroms)
Ta	-0.079154 ; -0.228010 ; -0.037161
C	-1.214495 ; 1.258186 ; 0.001732
H	-2.068224 ; 0.709591 ; 0.467987
H	-1.372432 ; 2.324352 ; -0.104440
C	1.757318 ; 0.715704 ; 0.056045
H	2.496192 ; -0.016988 ; -0.311468
H	1.890181 ; 1.674661 ; -0.445241
H	-0.321202 ; -0.735303 ; 1.615492
H	1.896785 ; 0.845096 ; 1.143776
****
022
Charge: Multiplicity: (2.0000)

E(Hartree):-136.496866   Zero-point:0.064166
Cartesian coordinates (Angstroms)
Ta 0.391506 ; -0.010454 ; -0.023923
C -1.803966 ; -0.706229 ; -0.056167
H -1.947346 ; -1.160192 ; -1.037899
H -1.860864 ; -1.388816 ; 0.792043
C -1.937654 ; 0.656034 ; 0.116653
H -2.037964 ; 1.098605 ; 1.102377
H -2.106377 ; 1.320311 ; -0.724702
H 0.627702 ; 1.709016 ; -0.193256
H 1.194629 ; -0.514604 ; 1.444910
****
008
Charge: Multiplicity: (2.0000)
E(Hartree):-136.512696   Zero-point:0.068091
Cartesian coordinates (Angstroms)
Ta -0.405474 ; -0.029697 ; -0.025526
C 1.472392 ; 0.851496 ; -0.030687
H 1.694364 ; 1.373281 ; -0.966431
H 1.664226 ; 1.506458 ; 0.820496
C 2.041304 ; -0.554809 ; 0.075706
H 2.582267 ; -0.740784 ; 1.000960
H 2.626829 ; -0.862519 ; -0.788936
H 1.222317 ; -1.359776 ; 0.116785
H -1.272537 ; 0.471085 ; 1.410399
****
015
Charge:1   Multiplicity:1 (0.0113)
E(Hartree):-136.50658   Zero-point:0.068171
Cartesian coordinates (Angstroms)
Ta -0.403989 ; -0.029718 ; -0.024944
C 1.471704 ; 0.851894 ; -0.023311
H 1.660085 ; 1.354391 ; -0.979094
H 1.696353 ; 1.524203 ; 0.804227
C 2.038166 ; -0.553702 ; 0.075149
H 2.578355 ; -0.748950 ; 0.998979
H 2.620081 ; -0.859185 ; -0.792875
H 1.216398 ; -1.358390 ; 0.115178
H -1.339302 ; 0.468208 ; 1.363463
****
021
Charge:1   Multiplicity:1 (0.0000)
E(Hartree):-136.493866   Zero-point:0.061480
Cartesian coordinates (Angstroms)
Ta -0.019761 ; -0.234953 ; -0.047172
C 1.486521 ; 0.967801 ; -0.042386
H 1.537903 ; 2.051124 ; 0.010666
H 0.652051 ; 0.110542 ; 1.701578
H 2.471539 ; 0.485185 ; -0.119733
C -1.389282 ; 1.071229 ; 0.013221
H -2.337132 ; 0.545838 ; 0.228943
H 0.016828 ; -0.423825 ; 1.860482
H -1.482048 ; 2.148561 ; -0.063411
****
085
Charge:1   Multiplicity:1 (0.0000)
E(Hartree):-136.527335   Zero-point:0.063541
Cartesian coordinates (Angstroms)
Ta 0.328077 ; -0.000062 ; 0.000010
C -1.550330 ; -0.787738 ; 0.000140
H -1.932789 ; -1.248413 ; -0.911796
H 1.192452 ; 0.001324 ; 1.532277
H -1.934260 ; -1.248421 ; 0.911397
C -1.550025 ; 0.787988 ; -0.000097
H -1.932934 ; 1.249114 ; 0.911398
H 1.192482 ; 0.000609 ; -1.532232
H -1.932425 ; 1.248785 ; -0.912011
****
275
Charge:1   Multiplicity:1 (0.0000)
E(Hartree):-136.492656   Zero-point:0.057123
Cartesian coordinates (Angstroms)
Ta 0.268737 ; 0.000399 ; -0.041771
C -1.608682 ; 0.664661 ; 0.183731
H -2.322683 ; 1.474646 ; 0.260017
C -1.608090 ; -0.667456 ; 0.177696
H -2.321289 ; -1.478843 ; 0.246218
H 0.228437 ; 1.087344 ; -1.391015
H 0.230508 ; -1.074272 ; -1.400810
H 1.933674 ; -0.390679 ; 1.580379
H 1.934205 ; 0.369431 ; 1.585951
****
276
Charge:1   Multiplicity:1 (0.0347)
E(Hartree):-136.478646   Zero-point:0.064872
Cartesian coordinates (Angstroms)
Ta -0.320907 ; 0.000112 ; -0.057062
C 1.652700 ; -0.739347 ; 0.121502
H 1.901062 ; -1.271672 ; 1.036125
H 1.999969 ; -1.249572 ; -0.777480
C 1.652835 ; 0.738879 ; 0.122461
H 1.999919 ; 1.250003 ; -0.776092
H 1.901788 ; 1.270088 ; 1.037571
H -2.103717 ; -0.389552 ; 1.091405
H -2.106002 ; 0.385361 ; 1.090189
****
278
Charge:1   Multiplicity:1 (0.0000)
E(Hartree):-136.483661   Zero-point:0.061262
Cartesian coordinates (Angstroms)
Ta -0.296525 ; -0.054482 ; -0.008470
C 1.647132 ; -0.553707 ; 0.297192
H 2.107172 ; -0.521294 ; 1.288781
C 1.290018 ; 0.844540 ; -0.369881
H 2.030791 ; 1.527519 ; -0.766739
H 2.175487 ; -1.216435 ; -0.394277
H -0.842639 ; -0.373503 ; -1.645441
H -1.060926 ; 1.227243 ; 1.409752
H -0.386481 ; 1.588682 ; 1.162396
****
281
Charge: Multiplicity: (0.0000)
E(Hartree):-136.51524   Zero-point:0.060716
Cartesian coordinates (Angstroms)
Ta -0.343786 ; -0.008764 ; -0.000007
C 1.554106 ; 0.755016 ; 0.000058
H 2.112768 ; 1.680438 ; 0.000086
H -0.517984 ; -1.753702 ; 0.000834
H -0.944498 ; 0.731112 ; 1.470264
C 1.975633 ; -0.499575 ; -0.000067
H 1.233266 ; -1.362891 ; -0.000016
H -0.944724 ; 0.730476 ; -1.470511
H 2.979121 ; -0.918281 ; -0.000077
****

294
Charge:1   Multiplicity:1 ()
E(Hartree):-136.492546   Zero-point:0.056890
Cartesian coordinates (Angstroms)
Ta   -0.272044 ; -0.000294 ; 0.045130
C   1.602002 ; 0.668497 ; -0.170536
H   2.319253 ; 1.476742 ; -0.233626
C   1.601750 ; -0.666547 ; -0.180510
H   2.318071 ; -1.474503 ; -0.256269
H   -0.227169 ; 1.078810 ; 1.401406
H   -0.227072 ; -1.099225 ; 1.385424
H   -1.478751 ; 0.035090 ; -1.981526
H   -2.067606 ; -0.007173 ; -1.503610
****
297
Charge:1   Multiplicity:5 (6.0000)
E(Hartree):-136.410309   Zero-point:0.060085
Cartesian coordinates (Angstroms)
Ta   -0.062507 ; -0.138310 ; 0.000063
C   -1.923286 ; 0.774859 ; -0.000167
H   -2.822461 ; 0.151776 ; -0.000647
H   -2.134068 ; 1.844819 ; 0.000218
C   2.328011 ; 0.575848 ; -0.000206
H   2.636504 ; 0.114973 ; -0.936447
H   2.112339 ; 1.642912 ; 0.000916
H   -0.297470 ; -1.876058 ; -0.000800
H   2.639829 ; 0.113956 ; 0.934398
****
314
Charge:1   Multiplicity:1 (0.0000)
E(Hartree):-136.50534   Zero-point:0.064786
Cartesian coordinates (Angstroms)
Ta   0.351774 ; -0.000005 ; -0.040118
C   -1.610439 ; -0.744241 ; 0.022764
H   -1.876776 ; -1.278110 ; -0.893025
H   -1.954929 ; -1.252015 ; 0.917714
C   -1.610430 ; 0.744259 ; 0.022737
H   -1.954799 ; 1.252028 ; 0.917738
H   -1.876886 ; 1.278146 ; -0.893001
H   0.654636 ; 1.038926 ; 1.302989
H   0.654450 ; -1.038720 ; 1.303189
****

Transition states

003
Charge:1   Multiplicity:3 (2.0000)
E(Hartree):-136.455262   Zero-point:0.059338
Cartesian coordinates (Angstroms)
Ta   -0.009428 ; -0.217657 ; -0.036608
C   1.779451 ; 0.817829 ; 0.088851
H   2.484123 ; 0.820301 ; -0.750679
H   2.117880 ; 1.437188 ; 0.917953
C   -1.441126 ; 1.102709 ; -0.095647
H   -1.522915 ; 2.115425 ; 0.293949
H   -2.349829 ; 0.772296 ; -0.620939
H   -0.762431 ; -0.754008 ; 1.588722
H   -1.308506 ; -0.025453 ; 1.284177
****
044
Charge:1   Multiplicity:1 (0.0000)
E(Hartree):-136.478085   Zero-point:0.062336
Cartesian coordinates (Angstroms)
Ta   -0.130974 ; -0.728474 ; -0.385716
C   1.131580 ; 0.618866 ; -0.217479
H   1.277825 ; 0.182464 ; 0.828064
H   1.698843 ; 1.519171 ; -0.412550
C   -1.855670 ; 0.319378 ; 0.077947
H   -2.796613 ; -0.224680 ; -0.061509
H   -1.328189 ; -0.164755 ; 1.067790
H   -1.328569 ; 0.390918 ; -1.221305
H   -2.042794 ; 1.360979 ; 0.324349
****
045
Charge:1   Multiplicity:3 (2.0000)
E(Hartree):-136.451237   Zero-point:0.061703
Cartesian coordinates (Angstroms)
Ta   -0.265253 ; 0.481809 ; -0.357403
C   1.392344 ; -0.552262 ; -0.023335
H   2.216026 ; 0.062770 ; 0.375429
H   1.660385 ; -1.603684 ; -0.100608
C   -2.079894 ; -0.423574 ; 0.141634
H   -2.350974 ; -1.473156 ; 0.053606
H   -1.449172 ; -0.337415 ; 1.127696
H   -1.607948 ; -0.138040 ; -1.406161
H   -2.958929 ; 0.220408 ; 0.231184
****
046
Charge:1   Multiplicity:3 (2.0000)
E(Hartree):-136.483029   Zero-point:0.061615
Cartesian coordinates (Angstroms)
Ta   -0.108765 ; 0.051002 ; 0.627713
C   -1.867683 ; 0.567880 ; -0.250787
H   -2.818293 ; 0.431244 ; 0.276683
H   -1.981449 ; 1.135495 ; -1.175149
C   1.621139 ; 0.984318 ; -0.075664
H   2.122974 ; 0.205150 ; -0.681083
H   1.473590 ; 1.857029 ; -0.715358
H   -0.716408 ; 1.582828 ; 1.222444
H   2.273903 ; 1.244614 ; 0.769908
****
047
Charge:1   Multiplicity:3 (2.0001)
E(Hartree):-136.447488   Zero-point:0.060685
Cartesian coordinates (Angstroms)
Ta   0.115832 ; 0.270043 ; -0.199361
C   -1.537490 ; 1.327560 ; 0.132791
H   -2.088221 ; 1.679322 ; -0.751038
H   -2.061075 ; 1.533886 ; 1.062676
C   -1.214126 ; -1.140522 ; 0.205504
H   -1.820361 ; -1.292489 ; 1.096464
H   -1.473582 ; -1.850482 ; -0.592962
H   1.554245 ; 0.109431 ; 1.258800
H   1.268952 ; -0.632681 ; 1.238749
****
049
Charge:1   Multiplicity:3 (2.0000)
E(Hartree):-136.455262   Zero-point:0.059342
Cartesian coordinates (Angstroms)
Ta   0.068793 ; -0.204654 ; 0.571892
C   -1.695246 ; 0.687001 ; -0.045532
H   -2.457457 ; 0.983039 ; 0.684561
H   -1.967143 ; 0.958008 ; -1.064497
C   1.506964 ; 1.068715 ; 0.245086
H   1.626641 ; 1.871409 ; -0.479781
H   2.373235 ; 0.966038 ; 0.915837

H 0.926813 ; -1.283955 ; -0.691158
H 1.458580 ; -0.486600 ; -0.635838
****
048
Charge:1 Multiplicity:3 (2.0000)
E(Hartree):-136.491755 Zero-point:0.064368
Cartesian coordinates (Angstroms)
Ta -0.460342 ; -0.333186 ; 0.888714
C -0.620046 ; 1.183943 ; -0.702869
H -0.960064 ; 0.671386 ; -1.605276
H -1.307273 ; 1.942206 ; -0.326274
C 0.757277 ; 1.321956 ; -0.471585
H 1.145808 ; 2.113551 ; 0.156861
H 1.484910 ; 0.851604 ; -1.122359
H 1.242191 ; 0.083819 ; 0.846910
H -1.283456 ; 0.224283 ; 2.334584
****
050
Charge:1 Multiplicity:3 (2.0000)
E(Hartree):-136.487758 Zero-point:0.061661
Cartesian coordinates (Angstroms)
Ta -0.175335 ; -0.115386 ; -0.142244
C -1.699401 ; 1.142957 ; -0.560487
H -2.633375 ; 0.759334 ; -0.984091
H -1.739154 ; 2.206252 ; -0.324532
H -1.316192 ; 0.020138 ; 1.204909
C 1.639509 ; 0.881586 ; 0.149083
H 1.894508 ; 0.831583 ; 1.218645
H 2.435220 ; 0.392558 ; -0.434013
H 1.593226 ; 1.940538 ; -0.128563
****
023
Charge:1 Multiplicity:3 (2.0000)
E(Hartree):-136.487758 Zero-point:0.061661
Cartesian coordinates (Angstroms)
Ta -0.175335 ; -0.115386 ; -0.142244
C -1.699401 ; 1.142957 ; -0.560487
H -2.633375 ; 0.759334 ; -0.984091
H -1.739154 ; 2.206252 ; -0.324532
H -1.316192 ; 0.020138 ; 1.204909
C 1.639509 ; 0.881586 ; 0.149083
H 1.894508 ; 0.831583 ; 1.218645
H 2.435220 ; 0.392558 ; -0.434013
H 1.593226 ; 1.940538 ; -0.128563
****
051
Charge:1 Multiplicity:3 (2.0000)
E(Hartree):-136.469319 Zero-point:0.063206
Cartesian coordinates (Angstroms)
Ta 0.477381 ; -0.009924 ; 0.016595
C -1.692598 ; -0.697821 ; 0.145628
H -1.914620 ; -1.249354 ; -0.766948
H -1.823601 ; -1.248909 ; 1.072625
C -1.684783 ; 0.704460 ; 0.133464
H -1.809479 ; 1.273108 ; 1.050709
H -1.900310 ; 1.242567 ; -0.788616
H 1.549706 ; 0.595614 ; 1.288745
H 1.542476 ; -0.605672 ; 1.299421
****
052
Charge:1 Multiplicity:3 (2.0000)
E(Hartree):-136.471714 Zero-point:0.063797
Cartesian coordinates (Angstroms)
Ta -0.271696 ; -0.074455 ; 0.061361
C 1.125724 ; 1.339191 ; 0.015072
H 1.524752 ; 1.700467 ; -0.939389
H 1.628181 ; 1.781886 ; 0.876035
C 1.792525 ; -0.825928 ; -0.019650
H 1.676173 ; -1.424796 ; -0.940623
H 2.770752 ; -0.352472 ; -0.048973
H -0.969387 ; -0.457256 ; 1.630956
H 1.742955 ; -1.470339 ; 0.871012
****
016
Charge:1 Multiplicity:1 (0.0000)
E(Hartree):-136.458994 Zero-point:0.065271
Cartesian coordinates (Angstroms)
Ta -0.236360 ; -0.766291 ; 0.257683
C -0.738383 ; 1.089550 ; 0.121122
H -1.139837 ; 1.308384 ; -0.883385
H -0.845592 ; 1.913652 ; 0.819340
C 1.298026 ; 0.964475 ; 0.082203
H 1.839197 ; 0.459173 ; -0.728148
H 1.202856 ; 2.011630 ; -0.200215
H -0.260468 ; -1.320055 ; 1.909292
H 1.865529 ; 0.886772 ; 1.008830
****
052-1
Charge:1 Multiplicity:1 (0.0000)
E(Hartree):-136.493065 Zero-point:0.060084
Cartesian coordinates (Angstroms)
Ta 0.175478 ; -0.069029 ; 0.575718
C -1.363291 ; 1.046505 ; 0.159893
H -1.419156 ; 2.093844 ; -0.121813
H -0.526649 ; 0.136050 ; -1.115961
H -2.345997 ; 0.558317 ; 0.214472
C 1.516008 ; 1.251172 ; 0.398750
H 2.457708 ; 0.699244 ; 0.219571
H 0.182658 ; -0.510465 ; -1.211679
H 1.614309 ; 2.330135 ; 0.360856
****
086
Charge: Multiplicity: (0.0000)
E(Hartree): Zero-point:
Cartesian coordinates (Angstroms)
Ta -0.345116 ; 0.028717 ; -0.114726
C 1.535786 ; 0.960944 ; -0.192980
H 1.938048 ; 1.369218 ; -1.118613
H 1.548112 ; 1.698263 ; 0.614447
C 1.878464 ; -0.422623 ; 0.209069
H 2.075367 ; -0.588668 ; 1.262669
H 2.556182 ; -0.992040 ; -0.423578
H 0.861087 ; -1.262882 ; -0.311334
H -0.510079 ; -0.142179 ; 1.611819
****
277
Charge:1 Multiplicity:1 (0.0000)
E(Hartree):-136.447396 Zero-point:0.060076
Cartesian coordinates (Angstroms)
Ta 0.669563 ; -0.111677 ; 0.067684
C -1.110957 ; 0.839364 ; -0.338815
H -1.626321 ; 1.534400 ; 0.318999
C -1.126870 ; -0.665774 ; 0.016437
H -1.967325 ; -1.348986 ; -0.010855

H -1.323463 ; 1.043489 ; -1.394982
H -0.185163 ; -1.196392 ; -1.107381
H 2.057674 ; -0.104574 ; 1.647766
H 1.347679 ; -0.004621 ; 2.001543
****
280
Charge:1 Multiplicity:1 (0.0000)
E(Hartree):-136.477009 Zero-point:0.059422
Cartesian coordinates (Angstroms)
Ta -0.343087 ; -0.009465 ; -0.010203
C 1.502674 ; 0.735840 ; 0.114900
H 2.079429 ; 1.623793 ; -0.111831
H -0.668637 ; -1.370584 ; 1.062396
H 0.635026 ; 1.030056 ; 1.192183
C 1.901141 ; -0.561855 ; -0.133920
H 1.377271 ; -1.444839 ; 0.268075
H -1.663240 ; 0.634503 ; -0.993205
H 2.862622 ; -0.825904 ; -0.558650
****
295-1
Charge:1 Multiplicity:1 (0.0000)
E(Hartree):-136.499275 Zero-point:0.058382
Cartesian coordinates (Angstroms)
Ta -0.408611 ; 0.145039 ; -0.345978
C 1.405529 ; -0.534062 ; 0.614180
H 1.955079 ; -1.143292 ; 1.319404
C 1.621541 ; 0.378435 ; -0.288493
H 2.436719 ; 0.930649 ; -0.733643
H -0.370252 ; -0.279794 ; -2.047121
H -0.672656 ; 1.839207 ; 0.023074
H -0.016223 ; -1.074468 ; 0.958410
H -1.035889 ; -1.113644 ; 0.850295
****
313
Charge:1 Multiplicity:1 (0.0000)
E(Hartree):-136.459519 Zero-point:0.059738
Cartesian coordinates (Angstroms)
Ta -0.276039 ; -0.000836 ; -0.034183
C 1.209273 ; 1.286672 ; -0.013702
H 1.166941 ; 2.123417 ; -0.724632
H 2.091997 ; 1.311598 ; 0.618686
C 1.217927 ; -1.278700 ; -0.014512
H 2.098831 ; -1.296116 ; 0.620772
H 1.185659 ; -2.115345 ; -0.725901
H -0.474257 ; -0.917448 ; 1.435872
H -0.481509 ; 0.907052 ; 1.439810
****
381-1
Charge:1 Multiplicity:1 ()
E(Hartree):-136.45809 Zero-point:0.064235
Cartesian coordinates (Angstroms)
Ta -0.132535 ; -0.261253 ; -0.000019
C -1.064478 ; 1.417384 ; -0.000113
H -1.314706 ; 1.941956 ; 0.925317
H 0.633324 ; 1.381469 ; -0.000138
H -1.314781 ; 1.944669 ; -0.923882
C 1.883265 ; 0.597980 ; 0.000029
H 2.203778 ; 0.064354 ; 0.909655
H 2.204397 ; 0.063880 ; -0.909057
H 2.350297 ; 1.582969 ; -0.000036
****

$TaC_2H_8^+$

Minima

246
Charge: Multiplicity: (6.0000)
E(Hartree):-137.707032 Zero-point:0.091615
Cartesian coordinates (Angstroms)
C -2.477570 ; -0.000002 ; 0.013103
H -1.941230 ; 0.946399 ; -0.234696
H -2.808985 ; 0.000296 ; 1.048347
H -3.327350 ; -0.000164 ; -0.669794
H -1.941337 ; -0.946598 ; -0.234223
Ta -0.000008 ; 0.000002 ; -0.000008
C 2.477626 ; -0.000022 ; -0.013067
H 3.327144 ; 0.000271 ; 0.670171
H 1.941095 ; 0.946415 ; 0.234235
H 2.809428 ; -0.000056 ; -1.048174
H 1.941445 ; -0.946580 ; 0.234470
****
247
Charge:1 Multiplicity:3 (2.0008)
E(Hartree):-137.704329 Zero-point:0.091747
Cartesian coordinates (Angstroms)
C -2.404691 ; 0.000709 ; -0.009655
H -1.853055 ; -0.971056 ; 0.132891
H -2.816494 ; 0.047920 ; -1.016048
H -3.200437 ; -0.045641 ; 0.734048
H -1.866790 ; 0.961034 ; 0.219991
Ta -0.000102 ; 0.000071 ; -0.000059
C 2.405402 ; -0.001226 ; 0.010153
H 3.202359 ; 0.044796 ; -0.732374
H 1.867798 ; -0.961517 ; -0.220253
H 2.815736 ; -0.048163 ; 1.017081
H 1.854100 ; 0.970571 ; -0.133994
****
248
Charge:1 Multiplicity:1 (0.0000)
E(Hartree):-137.734373 Zero-point:0.083606
Cartesian coordinates (Angstroms)
Ta 0.000032 ; -0.289959 ; -0.046136
C 1.474157 ; 1.187535 ; 0.047058
H 1.348936 ; 1.875459 ; -0.798503
H 1.614223 ; 1.739588 ; 0.975359
H 2.367755 ; 0.555088 ; -0.120333
C -1.474378 ; 1.187304 ; 0.047031
H -2.367682 ; 0.554399 ; -0.120363
H -1.614748 ; 1.739133 ; 0.975414
H -1.349557 ; 1.875378 ; -0.798440
H 0.915424 ; -0.710640 ; 1.345104
H -0.915351 ; -0.710440 ; 1.345146
****
249
Charge:1 Multiplicity:1 (0.0119)
E(Hartree):-137.732693 Zero-point:0.082046
Cartesian coordinates (Angstroms)
Ta 0.000007 ; -0.180691 ; -0.000016
C 1.777898 ; 0.913534 ; 0.000030
H 1.628647 ; 1.997988 ; 0.000276
H 2.358207 ; 0.628760 ; 0.891280
H 2.358225 ; 0.629163 ; -0.891333
C -1.778128 ; 0.913207 ; 0.000044

H	-2.358392 ; 0.628315 ; -0.891204
H	-2.358354 ; 0.628559 ; 0.891385
H	-1.629211 ; 1.997697 ; -0.000104
H	0.000791 ; -2.140258 ; -0.408532
H	0.000963 ; -2.140220 ; 0.408960
****
268
Charge:1 Multiplicity:3 (2.0000)
E(Hartree):-137.741687 Zero-point:0.082476
Cartesian coordinates (Angstroms)
Ta	0.000023 ; -0.184054 ; -0.000014
C	1.762192 ; 0.933118 ; 0.000002
H	1.596808 ; 2.015142 ; 0.000347
H	2.342336 ; 0.656859 ; 0.893427
H	2.342728 ; 0.657463 ; -0.893324
C	-1.763095 ; 0.931786 ; 0.000008
H	-2.343924 ; 0.656381 ; -0.893160
H	-2.343408 ; 0.656618 ; 0.893591
H	-1.596646 ; 2.013713 ; -0.000187
H	0.003098 ; -2.204861 ; -0.398696
H	0.002774 ; -2.204781 ; 0.398980
****
304
Charge:1 Multiplicity:1 ()
E(Hartree):-137.785494 Zero-point:0.080960
Cartesian coordinates (Angstroms)
Ta	-0.000001 ; 0.213740 ; -0.000020
C	-1.706279 ; -0.945476 ; -0.000230
H	-1.574750 ; -2.031069 ; -0.000597
H	-2.274005 ; -0.641500 ; 0.896246
H	-2.276825 ; -0.641092 ; -0.894623
C	1.706295 ; -0.945458 ; -0.000293
H	2.277237 ; -0.641522 ; -0.894543
H	2.273664 ; -0.641138 ; 0.896328
H	1.574677 ; -2.031044 ; -0.000189
H	-0.000134 ; 1.186481 ; -1.457754
H	0.000080 ; 1.183491 ; 1.459710
****
316
Charge:1 Multiplicity:3 (2.0000)
E(Hartree):-137.737172 Zero-point:0.084257
Cartesian coordinates (Angstroms)
C	1.977376 ; 0.672102 ; 0.021891
H	2.266266 ; 1.104280 ; -0.944374
H	2.561145 ; -0.255584 ; 0.184255
H	2.197387 ; 1.379556 ; 0.827047
H	0.236798 ; -1.860398 ; 0.132772
Ta	0.069710 ; -0.127124 ; -0.006710
C	-2.409721 ; 0.474118 ; 0.019626
H	-3.274938 ; 1.136124 ; 0.051442
H	-1.726420 ; 0.989512 ; -0.695917
H	-2.045562 ; 0.409979 ; 1.057470
H	-2.709442 ; -0.500722 ; -0.371976
****
317
Charge:1 Multiplicity:1 (0.0000)
E(Hartree):-137.726689 Zero-point:0.081980
Cartesian coordinates (Angstroms)
C	1.813825 ; 0.836836 ; -0.000142
H	2.289759 ; 0.402514 ; -0.900906
H	2.289568 ; 0.405536 ; 0.902154
H	1.937469 ; 1.919000 ; -0.001761
H	0.382652 ; -1.896341 ; -0.001705
Ta	0.024830 ; -0.176158 ; 0.000065
C	-1.941787 ; 0.693951 ; -0.000133
H	-2.113085 ; 1.772621 ; 0.000238
H	-1.468467 ; 0.457025 ; -1.097771
H	-1.469540 ; 0.455941 ; 1.097623
H	-2.893197 ; 0.158503 ; -0.000941
****

$TaC_3H_4^+$

Minima

101
Charge:1 Multiplicity:3 (2.0000)
E(Hartree):-173.386914 Zero-point:0.051598
Cartesian coordinates (Angstroms)
Ta	-0.139137 ; 0.283101 ; -0.032113
C	1.740875 ; -0.246247 ; 0.501048
H	2.637837 ; -0.148720 ; 1.101496
C	1.320459 ; -1.034034 ; -0.486235
H	1.655748 ; -1.902612 ; -1.036955
C	-1.467075 ; -1.251867 ; 0.274479
H	-2.175604 ; -1.446582 ; -0.539852
H	-1.526547 ; -1.975582 ; 1.083800
****
102
Charge:1 Multiplicity:1 (0.0000)
E(Hartree):-173.424499 Zero-point:0.052803
Cartesian coordinates (Angstroms)
Ta	-0.185275 ; -0.252780 ; 0.000008
C	1.568709 ; 0.410139 ; -0.674179
H	2.279518 ; 0.705352 ; -1.435825
C	1.568584 ; 0.410490 ; 0.674299
H	2.280506 ; 0.705592 ; 1.434986
C	-1.126540 ; 1.409744 ; -0.000058
H	-2.211441 ; 1.194387 ; 0.000399
H	-0.888055 ; 2.465363 ; -0.000528
****
103
Charge:1 Multiplicity:3 (2.0000)
E(Hartree):-173.378651 Zero-point:0.052932
Cartesian coordinates (Angstroms)
C	1.670918 ; -0.121209 ; -0.000134
C	1.283363 ; -1.355465 ; 0.000013
H	0.003333 ; -1.720745 ; 0.000510
H	1.757814 ; -2.326513 ; -0.000086
C	1.399476 ; 1.256413 ; 0.000072
H	1.652879 ; 1.800771 ; -0.908770
H	1.653200 ; 1.800519 ; 0.908981
Ta	-0.427257 ; 0.024213 ; -0.000005
****
104
Charge:1 Multiplicity:1 (0.0000)
E(Hartree):-173.369345 Zero-point:0.055035
Cartesian coordinates (Angstroms)
C	1.470141 ; 0.024368 ; -0.000193
C	2.618645 ; -0.655157 ; 0.000027
H	2.636702 ; -1.739361 ; -0.000224
H	3.580833 ; -0.150086 ; 0.000595
C	0.925759 ; 1.392532 ; -0.000027

H	0.996049 ; 1.993150 ; -0.910018
H	0.995962 ; 1.992732 ; 0.910263
Ta	-0.524614 ; -0.091327 ; 0.000007
****
219
Charge:1 Multiplicity:1 (0.0000)
E(Hartree):-173.394392 Zero-point:0.056403
Cartesian coordinates (Angstroms)
C	-0.990518 ; -1.240204 ; -0.000608
H	-1.388759 ; -2.245601 ; 0.000338
C	-1.920403 ; -0.000082 ; 0.000196
H	-2.542248 ; 0.000266 ; 0.900191
H	-2.545565 ; 0.000414 ; -0.897350
C	-0.990049 ; 1.240450 ; -0.000292
H	-1.389030 ; 2.245552 ; -0.000649
Ta	0.428376 ; -0.000022 ; 0.000023
****
299
Charge:1 Multiplicity:1 (0.0074)
E(Hartree):-173.410131 Zero-point:0.055617
Cartesian coordinates (Angstroms)
Ta	-0.437289 ; -0.003332 ; -0.000014
C	1.827940 ; 0.168690 ; -0.000030
H	2.916884 ; 0.192554 ; -0.000484
C	1.077170 ; 1.299258 ; 0.000152
H	1.441331 ; 2.315878 ; -0.000049
C	1.198006 ; -1.245903 ; 0.000034
H	1.473002 ; -1.798934 ; -0.901839
H	1.472205 ; -1.798527 ; 0.902431
****
299-1
Charge:1 Multiplicity:1 ()
E(Hartree):-173.398549 Zero-point:0.055253
Cartesian coordinates (Angstroms)
Ta	-0.432531 ; 0.001856 ; 0.000008
C	1.822263 ; 0.145283 ; 0.000121
H	2.911235 ; 0.142886 ; 0.000556
C	1.090427 ; 1.286051 ; -0.000139
H	1.473163 ; 2.296112 ; -0.000086
C	1.158427 ; -1.255021 ; -0.000114
H	1.382134 ; -1.816061 ; -0.910880
H	1.381547 ; -1.816304 ; 0.910636
****
300
Charge:1 Multiplicity:3 (2.0000)
E(Hartree):-173.415997 Zero-point:0.055787
Cartesian coordinates (Angstroms)
Ta	-0.440255 ; -0.006864 ; -0.000019
C	1.831752 ; 0.183477 ; -0.000125
H	2.920368 ; 0.229819 ; -0.000344
C	1.065571 ; 1.306571 ; 0.000179
H	1.418060 ; 2.327228 ; 0.000110
C	1.227618 ; -1.237668 ; 0.000013
H	1.526104 ; -1.785592 ; -0.897465
H	1.524423 ; -1.784656 ; 0.898694
****
353

Charge:1 Multiplicity:1 ()
E(Hartree):-173.399409 Zero-point:0.057039
Cartesian coordinates (Angstroms)
Ta	-0.559234 ; -0.084393 ; 0.000000
C	0.842925 ; 1.325134 ; 0.000033
H	1.092333 ; 2.379634 ; -0.000016
C	1.448455 ; 0.142606 ; -0.000153
C	2.775171 ; -0.496994 ; -0.000121
H	3.578048 ; 0.240789 ; -0.006325
H	2.879195 ; -1.136436 ; 0.881025
H	2.875168 ; -1.147754 ; -0.873268
****

$TaC_3H_6^+$

Minima
027
Charge:1 Multiplicity:3 (2.0002)
E(Hartree):-174.575586 Zero-point:0.067829
Cartesian coordinates (Angstroms)
C	0.573615 ; -1.624519 ; 0.747736
H	1.601616 ; -1.806751 ; 1.064003
H	-0.112273 ; -2.411430 ; 1.064508
C	-1.694247 ; 0.315710 ; 0.747356
H	-2.366565 ; -0.482740 ; 1.064281
H	-2.032207 ; 1.303528 ; 1.063655
Ta	0.000017 ; -0.000054 ; -0.271774
C	1.120438 ; 1.309156 ; 0.747390
H	0.764142 ; 2.290297 ; 1.064382
H	2.145184 ; 1.108967 ; 1.063775
****
028
Charge:1 Multiplicity:5 (6.0000)
E(Hartree):-174.54159 Zero-point:0.066709
Cartesian coordinates (Angstroms)
C	-0.637351 ; 1.992031 ; -0.000061
H	-1.685827 ; 2.296708 ; -0.000787
H	0.039200 ; 2.848995 ; 0.000391
C	2.043963 ; -0.444172 ; -0.000265
H	2.832190 ; 0.311361 ; -0.000616
H	2.447686 ; -1.458633 ; 0.000094
Ta	0.000068 ; -0.000048 ; 0.000056
C	-1.407100 ; -1.547521 ; -0.000124
H	-1.147978 ; -2.608157 ; 0.000670
H	-2.487322 ; -1.388789 ; -0.001173
****
029
Charge:1 Multiplicity:1 (0.0000)
E(Hartree):-174.641183 Zero-point:0.073205
Cartesian coordinates (Angstroms)
C	-1.601845 ; -0.387716 ; -0.764304
H	-1.704548 ; -1.349412 ; -1.253169
H	-2.178719 ; 0.402617 ; -1.250252
C	-1.601902 ; -0.386902 ; 0.764497
H	-2.178118 ; 0.404654 ; 1.249345
H	-1.705818 ; -1.347849 ; 1.254562
Ta	0.228619 ; 0.249256 ; -0.000066
C	1.163948 ; -1.375426 ; 0.000353
H	1.122786 ; -2.456537 ; 0.000291
H	2.194004 ; -0.948935 ; 0.000758
****
092
Charge:1 Multiplicity:3 (2.0000)
E(Hartree):-174.622941 Zero-point:0.077647
Cartesian coordinates (Angstroms)
Ta	-0.485991 ; 0.041220 ; -0.008692

C 1.695780 ; 1.069016 ; 0.200259
H 2.211086 ; 1.826683 ; -0.385950
H 1.990701 ; 1.100769 ; 1.247981
H 0.587937 ; 1.633663 ; 0.229767
C 1.608556 ; -0.238538 ; -0.447306
H 1.882236 ; -0.306682 ; -1.495169
C 1.102417 ; -1.398841 ; 0.246872
H 1.281178 ; -1.506267 ; 1.319313
H 1.083669 ; -2.347019 ; -0.280394
****
095
Charge:1 Multiplicity:1 (0.0190)
E(Hartree):-174.613887 Zero-point:0.077478
Cartesian coordinates (Angstroms)
Ta -0.484146 ; 0.041599 ; -0.008612
C 1.691438 ; 1.076712 ; 0.196465
H 2.208199 ; 1.825211 ; -0.400493
H 1.974756 ; 1.128792 ; 1.246458
H 0.579964 ; 1.643023 ; 0.219591
C 1.605521 ; -0.240364 ; -0.432542
H 1.859957 ; -0.306857 ; -1.486155
C 1.097934 ; -1.410027 ; 0.243221
H 1.263560 ; -1.539925 ; 1.314165
H 1.086877 ; -2.344927 ; -0.307772
****
096
Charge:1 Multiplicity:1 (0.0105)
E(Hartree):-174.644647 Zero-point:0.078645
Cartesian coordinates (Angstroms)
Ta -0.469044 ; -0.000004 ; 0.000014
C 1.927086 ; -0.000004 ; -0.000425
H 2.536831 ; 0.000110 ; 0.897183
H 2.535519 ; -0.000040 ; -0.898919
C 1.067619 ; 1.342447 ; 0.000081
H 1.198101 ; 1.938665 ; -0.903927
H 1.198653 ; 1.938476 ; 0.904116
C 1.067683 ; -1.342420 ; 0.000162
H 1.197894 ; -1.937735 ; 0.904830
H 1.198873 ; -1.939330 ; -0.903238
****
096-1
Charge:1 Multiplicity:1 ()
E(Hartree):-174.628561 Zero-point:0.077891
Cartesian coordinates (Angstroms)
Ta -0.465921 ; -0.000003 ; 0.000055
C 1.933648 ; -0.000001 ; -0.000510
H 2.537408 ; 0.000108 ; 0.900344
H 2.536220 ; -0.000051 ; -0.902155
C 1.058535 ; 1.331465 ; -0.000072
H 1.158209 ; 1.921247 ; -0.912028
H 1.158794 ; 1.921138 ; 0.911880
C 1.058574 ; -1.331446 ; 0.000032
H 1.158120 ; -1.920410 ; 0.912563
H 1.158909 ; -1.921950 ; -0.911343
****
090
Charge:1 Multiplicity:3 (2.0000)
E(Hartree):-174.651599 Zero-point:0.078896
Cartesian coordinates (Angstroms)
Ta -0.470867 ; -0.000014 ; -0.000029
C 1.927252 ; 0.000025 ; -0.000338
H 2.539225 ; 0.000035 ; 0.895974
H 2.537617 ; 0.000032 ; -0.897728
C 1.073433 ; 1.344971 ; 0.000328
H 1.212241 ; 1.942450 ; -0.901520
H 1.212944 ; 1.942199 ; 0.902206
C 1.073640 ; -1.344895 ; 0.000188
H 1.211853 ; -1.941099 ; 0.903012
H 1.213441 ; -1.943173 ; -0.900895
****
091
Charge:1 Multiplicity:1 (0.0000)
E(Hartree):-174.641183 Zero-point:0.073206
Cartesian coordinates (Angstroms)
Ta 0.228580 ; 0.249201 ; 0.000075
C -1.601775 ; -0.386795 ; -0.764589
H -2.178141 ; 0.404752 ; -1.249252
H -1.705791 ; -1.347839 ; -1.254401
C -1.601576 ; -0.387792 ; 0.764329
H -1.705433 ; -1.349398 ; 1.253064
H -2.177595 ; 0.403281 ; 1.250185
C 1.163799 ; -1.375322 ; -0.000253
H 2.193002 ; -0.946497 ; -0.001886
H 1.124961 ; -2.456489 ; -0.000137
****
024
Charge:1 Multiplicity:3 (2.0001)
E(Hartree):-174.606964 Zero-point:0.072756
Cartesian coordinates (Angstroms)
C 1.626635 ; 0.502706 ; -0.763588
H 1.648651 ; 1.469994 ; -1.259963
H 2.277857 ; -0.237411 ; -1.234333
C 1.626875 ; 0.501444 ; 0.763991
H 2.277725 ; -0.240081 ; 1.233088
H 1.649988 ; 1.467766 ; 1.262175
Ta -0.161915 ; -0.234836 ; -0.000108
C -1.816529 ; 0.998613 ; 0.000516
H -1.826995 ; 2.088220 ; 0.000915
H -2.829282 ; 0.577960 ; 0.000479
****
025
Charge:1 Multiplicity:5 (6.0000)
E(Hartree):-174.586849 Zero-point:0.074207
Cartesian coordinates (Angstroms)
C 1.924587 ; 0.481087 ; -0.690134
H 1.789997 ; 1.395204 ; -1.258375
H 2.308108 ; -0.371191 ; -1.249274
C 1.924471 ; 0.481490 ; 0.690084
H 2.307895 ; -0.370468 ; 1.249781
H 1.789800 ; 1.395936 ; 1.257775
Ta -0.224522 ; -0.246798 ; 0.000021
C -1.747755 ; 1.173149 ; -0.000143
H -1.605403 ; 2.253507 ; -0.000476
H -2.808085 ; 0.898892 ; 0.000156
****
160
Charge:1 Multiplicity:1 (0.0000)
E(Hartree):-174.644777 Zero-point:0.071634
Cartesian coordinates (Angstroms)
Ta 0.548430 ; -0.082093 ; -0.037493
C -1.452209 ; 0.121003 ; -0.024452
C -0.852613 ; 1.322625 ; 0.011008
H -1.143337 ; 2.364571 ; 0.065450
H 0.162495 ; -1.147663 ; 1.270048

H 1.097509 ; 0.769841 ; 1.363041
C -2.807918 ; -0.466099 ; 0.001942
H -3.062514 ; -0.687699 ; 1.043081
H -3.550219 ; 0.233128 ; -0.386029
H -2.862885 ; -1.404567 ; -0.549610
****
261
Charge:1 Multiplicity:1 (0.0000)
E(Hartree):-174.656518 Zero-point:0.071664
Cartesian coordinates (Angstroms)
Ta -0.093948 ; -0.291728 ; -0.065880
C 1.247516 ; 1.183972 ; -0.358462
H 1.510082 ; 2.131777 ; -0.810822
C 1.770076 ; 0.283732 ; 0.462232
H 2.677164 ; 0.136376 ; 1.033706
H -0.168465 ; -0.489946 ; 1.651607
C -1.594412 ; 1.123265 ; 0.262625
H -1.775104 ; 1.474448 ; -0.771577
H -2.453716 ; 0.515236 ; 0.591980
H -1.470808 ; 1.982464 ; 0.915995
****
264
Charge:1 Multiplicity:1 (0.0000)
E(Hartree):-174.609494 Zero-point:0.072876
Cartesian coordinates (Angstroms)
C -1.024914 ; 1.231153 ; -0.050305
H -1.446208 ; 2.227217 ; -0.081003
C -1.936364 ; -0.019529 ; -0.011319
H -2.568115 ; -0.050855 ; -0.903692
H -2.559968 ; 0.001862 ; 0.885978
C -0.986173 ; -1.250896 ; 0.043979
H -1.384556 ; -2.253344 ; 0.120939
Ta 0.423848 ; -0.004381 ; -0.048068
H 0.062268 ; 0.606247 ; 1.723492
H 0.640354 ; 0.024312 ; 1.869135
****
309
Charge:1 Multiplicity:1 (0.0000)
E(Hartree):-174.668578 Zero-point:0.070929
Cartesian coordinates (Angstroms)
Ta 0.098310 ; -0.284843 ; 0.006844
C -1.738938 ; 0.351596 ; -0.548367
H -2.626642 ; 0.351499 ; -1.169842
C -1.312409 ; 1.057302 ; 0.494348
H -1.636825 ; 1.863953 ; 1.137589
C 1.600782 ; 1.148565 ; -0.207131
H 1.869412 ; 1.398917 ; 0.832591
H 0.019557 ; -0.785216 ; 1.681544
H 1.466905 ; 2.063142 ; -0.780452
H 2.434321 ; 0.556503 ; -0.634112
****
322
Charge:1 Multiplicity:1 (0.0000)
E(Hartree):-174.652304 Zero-point:0.071395
Cartesian coordinates (Angstroms)
Ta 0.542280 ; -0.102434 ; 0.000000
C -1.466513 ; 0.085384 ; 0.000001
C -0.860045 ; 1.287283 ; 0.000005
H -1.131393 ; 2.335126 ; -0.000026
H 1.118972 ; 0.653415 ; -1.463466
H 1.119011 ; 0.653435 ; 1.463441
C -2.852840 ; -0.437361 ; -0.000017
H -3.013862 ; -1.070419 ; 0.876332
H -3.589235 ; 0.365822 ; -0.000621
H -3.013569 ; -1.071556 ; -0.875580
****
335
Charge:1 Multiplicity:1 ()
E(Hartree):-174.668579 Zero-point:0.070930
Cartesian coordinates (Angstroms)
Ta -0.098311 ; -0.284892 ; 0.006817
C 1.312356 ; 1.057158 ; 0.494553
H 1.636448 ; 1.863549 ; 1.138254
C 1.738823 ; 0.351861 ; -0.548440
H 2.626463 ; 0.352379 ; -1.170004
C -1.600712 ; 1.148803 ; -0.207030
H -1.465609 ; 2.063741 ; -0.779487
H -0.019559 ; -0.785678 ; 1.681391
H -1.869629 ; 1.398534 ; 0.832756
H -2.434250 ; 0.557628 ; -0.635063
****
364
Charge:1 Multiplicity:3 (2.0000)
E(Hartree):-174.636955 Zero-point:0.074088
Cartesian coordinates (Angstroms)
Ta 0.280545 ; -0.270813 ; -0.045534
C -2.503991 ; 0.719779 ; -0.244784
H -3.575736 ; 0.773042 ; -0.071545
C -1.704220 ; -0.083700 ; 0.477884
H -2.131814 ; -0.694196 ; 1.277299
C 1.324502 ; 1.514926 ; 0.187351
H 0.767452 ; 2.360251 ; 0.594057
H 2.177022 ; 1.282218 ; 0.846771
H -2.131611 ; 1.363733 ; -1.036448
H 1.717160 ; 1.778271 ; -0.808876
****
365
Charge:1 Multiplicity:1 ()
E(Hartree):-174.638154 Zero-point:0.072894
Cartesian coordinates (Angstroms)
Ta 0.085145 ; -0.290046 ; 0.011478
C -1.578440 ; 0.611401 ; -0.697308
H -2.425578 ; -0.032611 ; -0.959362
C -1.200831 ; 0.721818 ; 0.866381
H -1.695280 ; 1.430639 ; 1.517294
C 1.703742 ; 1.053049 ; -0.119227
H 1.453170 ; 1.840179 ; -0.841617
H 1.957951 ; 1.534374 ; 0.828581
H -1.626671 ; 1.586689 ; -1.180939
H 2.574006 ; 0.496515 ; -0.500899
****
366-1
Charge:1 Multiplicity:3 (2.0000)
E(Hartree):-174.640453 Zero-point:0.074874
Cartesian coordinates (Angstroms)
Ta -0.017596 ; -0.026661 ; 0.000436
C 1.818714 ; 0.753576 ; -0.000929
H 2.548363 ; 1.553290 ; -0.001346
C 1.895469 ; -0.593797 ; -0.001382
H 2.714857 ; -1.301415 ; -0.002547
C -2.609193 ; 0.080663 ; -0.001327
H -3.666380 ; -0.183858 ; -0.002807
H -2.204795 ; -0.367540 ; -0.928295
H -2.530487 ; 1.170529 ; -0.001505

H	-2.206964 ; -0.367390 ; 0.926526
****
367
Charge:1 Multiplicity:1 ()
E(Hartree):-174.61409 Zero-point:0.074373
Cartesian coordinates (Angstroms)
Ta	0.020437 ; -0.316697 ; -0.035327
C	-1.029912 ; 1.360434 ; -0.300065
H	-1.104729 ; 2.385090 ; -0.637692
C	-1.765075 ; 0.473396 ; 0.384278
H	-2.781999 ; 0.429113 ; 0.754211
C	1.905490 ; 0.918427 ; 0.183782
H	2.182674 ; 1.954597 ; 0.371100
H	1.263568 ; 0.649238 ; 1.136049
H	1.496141 ; 0.910372 ; -0.878482
H	2.789459 ; 0.276904 ; 0.225697
****
368
Charge:1 Multiplicity:3 (2.0000)
E(Hartree):-174.614867 Zero-point:0.067159
Cartesian coordinates (Angstroms)
Ta	0.131108 ; -0.240853 ; 0.016931
C	-2.048217 ; 0.223542 ; -0.470795
H	-2.716649 ; -0.292014 ; -1.142304
C	-1.657438 ; 1.017042 ; 0.409330
H	-1.755146 ; 1.831395 ; 1.112476
C	1.748526 ; 1.064139 ; -0.206377
H	2.608023 ; 0.488908 ; 0.179578
H	0.443028 ; -1.578399 ; 1.125706
H	1.669867 ; 1.997757 ; 0.359340
H	1.922730 ; 1.306306 ; -1.263729
****

Transition states

369-1
Charge: Multiplicity: (2.0000)
E(Hartree):-174.605665 Zero-point:0.071685
Cartesian coordinates (Angstroms)
Ta	-0.051868 ; -0.333516 ; 0.000001
C	1.089517 ; 1.446292 ; 0.000010
H	1.227934 ; 2.520879 ; 0.000026
C	1.859845 ; 0.374824 ; -0.000013
H	2.925406 ; 0.194254 ; -0.000030
C	-1.835416 ; 1.003212 ; -0.000006
H	-2.029706 ; 2.076890 ; 0.000021
H	-2.316451 ; 0.593323 ; -0.901327
H	-0.387987 ; 1.422136 ; 0.000012
H	-2.316513 ; 0.593254 ; 0.901251
****

$TaC_3H_8^+$

Minima

097
Charge:1 Multiplicity:3 (2.0000)
E(Hartree):-175.862456 Zero-point:0.089668
Cartesian coordinates (Angstroms)
Ta	-0.000004 ; 0.017716 ; -0.111743
C	-0.000879 ; 2.060028 ; 0.295919
H	-0.908265 ; 2.653731 ; 0.420674
H	0.906061 ; 2.654457 ; 0.420510
C	1.781778 ; -0.993317 ; 0.301760
H	1.652455 ; -1.961320 ; 0.795269
H	2.211953 ; -1.167710 ; -0.703961
H	2.495647 ; -0.385293 ; 0.868672
C	-1.780951 ; -0.994689 ; 0.301796
H	-2.210732 ; -1.168462 ; -0.704243
H	-1.651041 ; -1.963041 ; 0.794451
H	-2.495445 ; -0.387736 ; 0.869042
****
098
Charge:1 Multiplicity:1 (0.0000)
E(Hartree):-175.890186 Zero-point:0.091165
Cartesian coordinates (Angstroms)
Ta	0.000034 ; 0.034644 ; -0.231741
C	-0.000329 ; 1.734019 ; 0.634905
H	-0.913194 ; 2.231849 ; 0.966673
H	0.911998 ; 2.232777 ; 0.966710
C	1.694729 ; -0.900337 ; 0.578625
H	1.978725 ; -1.683470 ; -0.144603
H	2.542313 ; -0.227473 ; 0.730116
H	1.450909 ; -1.384323 ; 1.530611
C	-1.694572 ; -0.900768 ; 0.578505
H	-2.542262 ; -0.227568 ; 0.728180
H	-1.977864 ; -1.684635 ; -0.144180
H	-1.452053 ; -1.383613 ; 1.531354
****
099
Charge:1 Multiplicity:1 (0.0271)
E(Hartree):-175.846342 Zero-point:0.096586
Cartesian coordinates (Angstroms)
Ta	-0.069339 ; 0.028511 ; -0.000018
C	1.928805 ; 0.669133 ; 0.000161
H	2.282239 ; 1.160162 ; -0.904934
H	2.282073 ; 1.160186 ; 0.905307
C	1.848249 ; -0.824361 ; 0.000172
H	2.145682 ; -1.350402 ; 0.905756
H	2.145835 ; -1.350418 ; -0.905353
C	-2.632299 ; -0.086938 ; -0.000144
H	-2.752232 ; -1.170042 ; -0.000141
H	-2.148790 ; 0.266666 ; 0.936636
H	-2.148726 ; 0.266655 ; -0.936892
H	-3.612897 ; 0.388919 ; -0.000176
****
099-1
Charge:1 Multiplicity:1 ()
E(Hartree):-175.836063 Zero-point:0.096323
Cartesian coordinates (Angstroms)
Ta	0.073467 ; -0.024333 ; -0.000012
C	-1.905812 ; -0.681408 ; 0.000143
H	-2.251570 ; -1.177905 ; -0.905297
H	-2.251433 ; -1.177929 ; 0.905621
C	-1.833661 ; 0.813721 ; 0.000153
H	-2.128235 ; 1.341639 ; 0.906235
H	-2.128375 ; 1.341655 ; -0.905874
C	2.570676 ; 0.074084 ; -0.000157
H	2.784141 ; 1.143233 ; -0.000164
H	2.060607 ; -0.221996 ; 0.951013
H	2.060511 ; -0.221990 ; -0.951276
H	3.504025 ; -0.488814 ; -0.000204
****
323-1
Charge:1 Multiplicity:3 (2.0000)
E(Hartree):-175.849354 Zero-point:0.096523
Cartesian coordinates (Angstroms)

Ta -0.064418 ; -0.046458 ; -0.094645
C 1.904485 ; -0.625013 ; 0.327535
H 2.145635 ; -0.994603 ; 1.321219
H 2.412754 ; -1.181179 ; -0.460229
C 1.807681 ; 0.872953 ; 0.143089
H 2.255643 ; 1.285580 ; -0.760362
H 1.978948 ; 1.496052 ; 1.017462
C -2.623012 ; 0.139113 ; 0.287682
H -2.690507 ; 1.214169 ; 0.123057
H -1.935740 ; -0.107745 ; 1.129923
H -3.586666 ; -0.234108 ; 0.634691
H -2.412510 ; -0.409022 ; -0.646518
****
221
Charge:1 Multiplicity:1 (0.0000)
E(Hartree):-175.892365 Zero-point:0.091204
Cartesian coordinates (Angstroms)
Ta -0.126931 ; -0.234268 ; 0.000001
C 1.593075 ; 0.558315 ; 0.787247
H 1.602530 ; 1.545365 ; 1.245916
H 2.307118 ; -0.129355 ; 1.243014
C 1.593874 ; 0.557078 ; -0.787245
H 2.308441 ; -0.131307 ; -1.241113
H 1.603877 ; 1.543470 ; -1.247335
C -1.824810 ; 0.988234 ; -0.000360
H -1.836156 ; 1.637155 ; -0.887811
H -0.326516 ; -1.991112 ; 0.002417
H -1.835061 ; 1.638420 ; 0.886203
H -2.731081 ; 0.367150 ; 0.000771
****
226
Charge:1 Multiplicity:3 (2.0000)
E(Hartree):-175.861616 Zero-point:0.096361
Cartesian coordinates (Angstroms)
Ta 0.226090 ; -0.297853 ; -0.000002
C -1.872846 ; -0.274059 ; 0.000018
H -2.315769 ; -0.722072 ; -0.893234
H -2.315765 ; -0.722041 ; 0.893286
C -1.799005 ; 1.237008 ; -0.000009
H -2.210159 ; 1.696426 ; 0.897073
H -2.210199 ; 1.696397 ; -0.897088
C 1.586095 ; 1.319025 ; 0.000011
H 2.194845 ; 1.052717 ; -0.885576
H -0.725770 ; 1.609694 ; -0.000040
H 2.194876 ; 1.052673 ; 0.885563
H 1.397919 ; 2.387663 ; 0.000041
****
244
Charge:1 Multiplicity:1 (0.0000)
E(Hartree):-175.863793 Zero-point:0.089156
Cartesian coordinates (Angstroms)
Ta -0.017550 ; -0.187432 ; 0.018893
C 1.648370 ; 0.789050 ; 0.579599
H 2.257987 ; 1.275477 ; 1.329744
C 1.718649 ; 0.505996 ; -0.721490
H 2.414757 ; 0.654314 ; -1.536651
H 0.368187 ; -1.155400 ; 1.412002
H 0.488035 ; -1.645591 ; -0.786471
C -2.426095 ; 0.666593 ; -0.089183
H -1.707937 ; 1.392632 ; -0.515851
H -2.329814 ; 0.549735 ; 0.997198
H -3.394409 ; 1.135354 ; -0.268596
H -2.461165 ; -0.293834 ; -0.624150
****
273
Charge:1 Multiplicity:1 (0.0000)
E(Hartree):-175.850035 Zero-point:0.085078
Cartesian coordinates (Angstroms)
Ta -0.106093 ; -0.242354 ; 0.022258
C 1.362825 ; 1.009069 ; -0.589647
H 1.692035 ; 1.818714 ; -1.228283
C 1.839890 ; 0.222367 ; 0.360276
H 2.761314 ; 0.072626 ; 0.907592
H -0.129719 ; -0.159143 ; 1.757042
C -1.472082 ; 1.333478 ; 0.101792
H -1.643291 ; 1.457664 ; -0.987245
H -2.365576 ; 0.875643 ; 0.558273
H -1.278099 ; 2.305528 ; 0.545760
H -0.626001 ; -2.267635 ; -0.992800
H -1.049659 ; -1.801029 ; -1.419685
****
286
Charge:1 Multiplicity:1 (0.0000)
E(Hartree):-175.845502 Zero-point:0.091333
Cartesian coordinates (Angstroms)
Ta -0.001022 ; -0.242008 ; -0.001422
C 1.183548 ; 0.910710 ; 0.813571
H 1.756707 ; 1.508917 ; 1.509388
H 0.403645 ; -1.666169 ; 0.936441
C 1.700691 ; 0.596766 ; -0.691005
H 2.641597 ; 0.038623 ; -0.696031
H 1.715673 ; 1.453579 ; -1.368194
C -2.360046 ; 0.710185 ; -0.112004
H -3.183958 ; 1.344628 ; 0.218997
H -1.489168 ; 1.283912 ; 0.299900
H -2.538795 ; -0.273427 ; 0.342856
H -2.376256 ; 0.670547 ; -1.202897
****
301
Charge:1 Multiplicity:1 ()
E(Hartree):-175.845631 Zero-point:0.093429
Cartesian coordinates (Angstroms)
Ta -0.461267 ; 0.000033 ; -0.059084
C 1.915272 ; -0.000083 ; -0.336850
H 1.958193 ; -0.000096 ; -1.429272
H 2.949367 ; -0.000644 ; 0.008941
C 1.185838 ; -1.229709 ; 0.287820
H 1.524101 ; -1.456938 ; 1.294275
H 1.121335 ; -2.134603 ; -0.324770
C 1.186367 ; 1.229489 ; 0.287761
H 1.122038 ; 2.134446 ; -0.324738
H 1.524409 ; 1.456644 ; 1.294304
H -1.126536 ; -0.989506 ; 1.180931
H -1.125313 ; 0.990146 ; 1.181057
****
320
Charge:1 Multiplicity:1 ()
E(Hartree):-175.870785 Zero-point:0.089857
Cartesian coordinates (Angstroms)
Ta -0.002534 ; -0.257623 ; -0.019374
C -1.117758 ; 1.433112 ; -0.109896
H -1.241121 ; 2.497088 ; -0.267288
C -1.896122 ; 0.394409 ; 0.173835
H -2.943458 ; 0.201203 ; 0.362101

H -0.506097 ; -1.280338 ; -1.355961
H -0.215737 ; -0.921143 ; 1.586125
C 2.304815 ; 0.716692 ; 0.066855
H 3.120397 ; 1.429559 ; 0.195016
H 2.143872 ; 0.260204 ; 1.059003
H 1.467340 ; 1.373776 ; -0.266525
H 2.614201 ; -0.019170 ; -0.682907
****
331
Charge:1 Multiplicity:1 ()
E(Hartree):-175.883701 Zero-point:0.088482
Cartesian coordinates (Angstroms)
Ta 0.216075 ; -0.249197 ; -0.042542
C -2.125581 ; 0.748942 ; -0.330077
H -3.161574 ; 1.047148 ; -0.474185
C -1.681799 ; -0.013041 ; 0.680091
H -2.309983 ; -0.387221 ; 1.480177
H 0.126097 ; -0.502497 ; -1.774182
H 1.122637 ; -1.555298 ; 0.716154
C 1.329586 ; 1.473374 ; 0.234642
H 0.916963 ; 2.317554 ; 0.787661
H 2.222102 ; 1.080825 ; 0.757765
H -1.470303 ; 1.135369 ; -1.125468
H 1.647350 ; 1.799825 ; -0.770272
****
339
Charge:1 Multiplicity:1 ()
E(Hartree):-175.824877 Zero-point:0.094866
Cartesian coordinates (Angstroms)
Ta -0.472503 ; -0.020248 ; -0.014395
C 1.022202 ; 1.393693 ; -0.007663
H 1.090606 ; 1.943837 ; -0.949785
H 1.080000 ; 2.049639 ; 0.860137
C 1.995314 ; 0.164038 ; 0.048889
H 2.567737 ; 0.184921 ; 0.971408
H 2.656949 ; 0.184118 ; -0.813431
H -2.093358 ; 0.298351 ; 0.643176
C 1.283878 ; -1.265400 ; 0.001745
H 0.612169 ; -1.391788 ; 0.953624
H 0.775125 ; -1.466558 ; -1.006724
H 1.995089 ; -2.078407 ; 0.134594
****
340
Charge:1 Multiplicity:3 (2.0000)
E(Hartree):-175.84242 Zero-point:0.096139
Cartesian coordinates (Angstroms)
Ta -0.519817 ; -0.062068 ; -0.007323
C 0.911851 ; 1.402091 ; -0.250762
H 1.164304 ; 1.584786 ; -1.300990
H 0.871089 ; 2.335366 ; 0.307627
C 1.905368 ; 0.351372 ; 0.382949
H 1.879570 ; 0.365324 ; 1.473568
H 2.902851 ; 0.687591 ; 0.091178
H -1.979969 ; 0.251441 ; 0.941568
C 1.805521 ; -1.103472 ; -0.157865
H 0.858935 ; -1.693964 ; 0.049511
H 1.984846 ; -1.164607 ; -1.232195
H 2.528591 ; -1.734902 ; 0.358372
****
341
Charge:1 Multiplicity:1 ()
E(Hartree):-175.861726 Zero-point:0.091614
Cartesian coordinates (Angstroms)
Ta 0.530993 ; -0.082683 ; -0.007974
C -0.830463 ; 1.386847 ; -0.277020
H -0.767883 ; 2.297705 ; 0.320089
H 0.895292 ; -1.132668 ; -1.375117
H -1.297438 ; 1.537599 ; -1.251168
C -1.424972 ; 0.112665 ; 0.525452
H 1.705306 ; 0.156770 ; 1.281763
H -1.562053 ; 0.357305 ; 1.584092
C -2.586864 ; -0.624767 ; -0.111713
H -2.828643 ; -1.514788 ; 0.469855
H -3.471570 ; 0.015930 ; -0.127872
H -2.381705 ; -0.930489 ; -1.139835
****
343
Charge:1 Multiplicity:1 ()
E(Hartree):-175.883701 Zero-point:0.088485
Cartesian coordinates (Angstroms)
Ta 0.216042 ; -0.249205 ; -0.042533
C -1.682250 ; -0.013684 ; 0.679609
H -2.310466 ; -0.388913 ; 1.479187
H 1.123719 ; -1.555229 ; 0.714911
C -2.125314 ; 0.749514 ; -0.329853
H -1.468900 ; 1.135975 ; -1.124300
H 0.126919 ; -0.501530 ; -1.774343
H -3.161017 ; 1.048464 ; -0.474163
C 1.329652 ; 1.473243 ; 0.235119
H 2.223030 ; 1.082140 ; 0.757648
H 1.646188 ; 1.798748 ; -0.770663
H 0.916907 ; 2.317897 ; 0.787356
****
345
Charge:1 Multiplicity:1 ()
E(Hartree):-175.850035 Zero-point:0.085069
Cartesian coordinates (Angstroms)
Ta -0.106040 ; -0.242464 ; -0.022511
C 1.839961 ; 0.223073 ; -0.359967
H 2.761494 ; 0.073378 ; -0.907104
C 1.362425 ; 1.009112 ; 0.590167
H 1.690291 ; 1.818900 ; 1.229313
H -0.129744 ; -0.158973 ; -1.757349
H -1.044063 ; -1.797093 ; 1.427583
H -0.629822 ; -2.266465 ; 0.994607
C -1.472342 ; 1.333164 ; -0.101547
H -2.366026 ; 0.875775 ; -0.558002
H -1.643311 ; 1.456977 ; 0.987550
H -1.278133 ; 2.305302 ; -0.545206
****
351
Charge:1 Multiplicity:1 (0.0000)
E(Hartree):-175.866669 Zero-point:0.087104
Cartesian coordinates (Angstroms)
Ta -0.114025 ; -0.244754 ; -0.081689
C 1.809780 ; 0.338611 ; 0.537465
H 2.693846 ; 0.205743 ; 1.144413
C 1.338373 ; 1.164071 ; -0.352727
H 1.576077 ; 2.066091 ; -0.898443
H -0.107674 ; -0.546122 ; -1.813485
H 0.840265 ; -1.391878 ; 1.258846
H 0.158900 ; -1.816264 ; 1.202303
C -1.638269 ; 1.086253 ; 0.429703
H -1.929781 ; 1.534999 ; -0.535051

H -2.455731 ; 0.409015 ; 0.745928
H -1.511373 ; 1.871841 ; 1.172112
****
348
Charge:1 Multiplicity:1 ()
E(Hartree):-175.883994 Zero-point:0.087883
Cartesian coordinates (Angstroms)
Ta 0.159782 ; -0.223397 ; 0.049301
C -2.182529 ; 0.230121 ; -0.440330
H -3.218854 ; 0.331425 ; -0.752231
C -1.489485 ; 0.953081 ; 0.429326
H -1.831309 ; 1.789346 ; 1.023092
H 0.508266 ; -1.046895 ; 1.563330
H -1.701694 ; -0.616972 ; -1.004040
H 0.043202 ; -1.331896 ; -1.310698
C 1.728718 ; 1.066394 ; -0.328669
H 2.433604 ; 0.700354 ; 0.449332
H 2.158093 ; 0.840984 ; -1.316213
H 1.604387 ; 2.144075 ; -0.213537
****
350
Charge:1 Multiplicity:1 ()
E(Hartree):-175.841793 Zero-point:0.092747
Cartesian coordinates (Angstroms)
Ta 0.778513 ; 0.037531 ; -0.041017
H 1.155241 ; 0.683644 ; 1.538565
H 1.090786 ; -1.672639 ; 0.088451
C -1.051287 ; -0.191364 ; 0.133087
H -0.803341 ; 0.598618 ; 0.954608
C -2.458642 ; -0.647994 ; 0.093600
H -2.674888 ; -1.170315 ; 1.032095
H -2.541135 ; -1.393803 ; -0.701347
C -3.468154 ; 0.491120 ; -0.124247
H -3.425007 ; 1.220606 ; 0.685352
H -3.288975 ; 1.006368 ; -1.067917
H -4.475617 ; 0.077217 ; -0.150221
****
349-1
Charge:1 Multiplicity:1 (0.0000)
E(Hartree):-175.860416 Zero-point:0.091849
Cartesian coordinates (Angstroms)
Ta -0.454031 ; -0.048367 ; 0.006076
H -1.709850 ; 0.119101 ; 1.241813
C 1.000718 ; 1.434612 ; -0.330347
H 1.278417 ; 1.491876 ; -1.387209
H -0.817128 ; -0.965588 ; -1.447098
H 0.971930 ; 2.407114 ; 0.153111
C 1.534114 ; 0.311217 ; 0.488670
H 1.847433 ; 0.516813 ; 1.506964
C 1.820084 ; -1.025341 ; -0.102665
H 2.302632 ; -1.042031 ; -1.080153
H 2.310700 ; -1.699036 ; 0.595526
H 0.830637 ; -1.620352 ; -0.360485
****

Transition states

222
Charge:1 Multiplicity:1 (0.0000)
E(Hartree):-175.822448 Zero-point:0.090766
Cartesian coordinates (Angstroms)
Ta -0.212454 ; -0.058080 ; -0.515220
C 1.599159 ; 0.739646 ; 0.065515
H 2.173126 ; 1.201334 ; -0.744571
H 1.784667 ; 1.220335 ; 1.020865
C 1.627223 ; -0.822417 ; 0.020505
H 1.830867 ; -1.350316 ; 0.946798
H 2.216528 ; -1.215840 ; -0.814222
C -2.352495 ; -0.108664 ; -0.089750
H -2.887485 ; -1.018074 ; 0.188653
H -1.308257 ; -0.118906 ; 0.914422
H -2.282945 ; -0.074483 ; -1.232588
H -2.919335 ; 0.763536 ; 0.239968
****
223
Charge:1 Multiplicity:3 (2.0000)
E(Hartree):-175.831658 Zero-point:0.094364
Cartesian coordinates (Angstroms)
Ta -0.213948 ; -0.868525 ; 0.549336
C 1.860207 ; -0.613272 ; 0.147029
H 2.416326 ; -0.200246 ; 0.988901
H 2.332930 ; -1.503417 ; -0.268638
C 1.197340 ; 0.309438 ; -0.764896
H 1.206149 ; 0.073426 ; -1.825813
H 1.289764 ; 1.374749 ; -0.570236
C -1.876206 ; 0.208859 ; -0.457625
H -2.331226 ; 0.589552 ; 0.471530
H -0.471951 ; 0.365995 ; -0.712571
H -2.411957 ; -0.701368 ; -0.773695
H -2.026796 ; 0.963860 ; -1.230557
****
237
Charge:1 Multiplicity:3 (2.0000)
E(Hartree):-175.76141 Zero-point:0.088113
Cartesian coordinates (Angstroms)
Ta 0.076569 ; -0.279121 ; -0.013173
C -1.600373 ; 0.745159 ; -0.711960
H -1.522663 ; 1.747990 ; -1.122073
H -2.294675 ; 0.117890 ; -1.275964
C -1.649944 ; 0.583116 ; 0.801019
H -2.400572 ; -0.117136 ; 1.171863
H -1.597359 ; 1.488820 ; 1.399259
C 1.934022 ; 0.846397 ; -0.130698
H 1.837770 ; 1.931611 ; -0.164292
H 2.433486 ; 0.607052 ; 1.068233
H 2.699890 ; 0.492624 ; -0.826820
H 3.152342 ; 1.058980 ; 0.961253
****
274
Charge:1 Multiplicity:1 (0.0000)
E(Hartree):-175.817784 Zero-point:0.086339
Cartesian coordinates (Angstroms)
Ta -0.296482 ; -0.206831 ; 0.507143
C 1.051252 ; 0.714507 ; -0.642345
H 1.415931 ; 1.078060 ; -1.594249
C 1.432620 ; 0.786619 ; 0.645726
H 2.278905 ; 1.210234 ; 1.172384
H -1.417448 ; 1.159288 ; 0.926657
C -1.962770 ; 0.816293 ; -0.410528
H -1.631296 ; 0.078528 ; -1.218130
H -2.958291 ; 0.515800 ; -0.075082
H -2.000478 ; 1.802471 ; -0.863635
H 0.406853 ; -2.089216 ; 1.060484
H 0.240218 ; -2.178858 ; 0.286620
****

285
Charge:1   Multiplicity:1 (0.0000)
E(Hartree):-175.811843   Zero-point:0.090132
Cartesian coordinates (Angstroms)
Ta 0.055581 ; -0.015120 ; -0.059715
C -1.842370 ; -0.660161 ; 0.225932
H -1.393724 ; -1.154759 ; -0.768598
H -2.666046 ; -1.279877 ; 0.552240
C -1.705456 ; 0.763125 ; 0.245363
H -2.424358 ; 1.553803 ; 0.406030
H -0.944054 ; 1.184989 ; -0.988810
C 2.446779 ; 0.013205 ; 0.221951
H 2.325571 ; 0.481043 ; -0.771768
H 1.866596 ; 0.510974 ; 1.046278
H 2.306998 ; -1.079170 ; 0.199201
H 3.477876 ; 0.189764 ; 0.525138
****
287
Charge:1   Multiplicity:1 (0.0000)
E(Hartree):-175.834669   Zero-point:0.088332
Cartesian coordinates (Angstroms)
Ta -0.020614 ; 0.222485 ; -0.040618
C 1.652890 ; 1.023766 ; 0.518154
H 2.369289 ; 1.431732 ; 1.217380
C 1.795021 ; 0.497576 ; -0.785802
H 2.576550 ; 0.606413 ; -1.525799
H 0.241525 ; -0.946817 ; 1.226805
H 1.382715 ; -0.750768 ; -0.812705
C -2.512291 ; 0.707795 ; 0.087456
H -1.796791 ; 1.562813 ; 0.141186
H -2.352122 ; -0.062403 ; 0.856099
H -3.459754 ; 1.190541 ; 0.326318
H -2.603772 ; 0.278646 ; -0.914946
****
324
Charge:1   Multiplicity:1 ()
E(Hartree):-175.815667   Zero-point:0.091173
Cartesian coordinates (Angstroms)
Ta 0.048632 ; 0.071545 ; -0.069865
C -1.702934 ; 0.535846 ; 0.482728
H -2.535369 ; 1.181534 ; 0.732570
H -1.152877 ; 1.141474 ; -0.859436
C -1.767487 ; -0.923449 ; 0.023419
H -2.388484 ; -1.088610 ; -0.860920
H -1.909801 ; -1.700719 ; 0.771596
C 2.507405 ; -0.243724 ; 0.218293
H 3.545185 ; -0.198817 ; 0.547123
H 1.935447 ; -0.070514 ; 1.160459
H 2.397204 ; 0.547278 ; -0.544555
H 2.336639 ; -1.246460 ; -0.193360
****
329
Charge:1   Multiplicity:1 ()
E(Hartree):-175.822457   Zero-point:0.088666
Cartesian coordinates (Angstroms)
Ta 0.040484 ; -0.305374 ; -0.000760
C -0.947104 ; 1.161456 ; -0.656250
H -1.195654 ; 2.039949 ; -1.237161
H -0.081488 ; -1.399056 ; -1.372162
C -1.751859 ; 0.565448 ; 0.537272
H -2.669143 ; 0.041864 ; 0.260923
H -1.895575 ; 1.259583 ; 1.367320
C 1.928199 ; 0.930175 ; 0.191629
H 2.264139 ; 0.567811 ; 1.171946
H 0.569451 ; 1.416080 ; -0.115294
H 2.177875 ; 1.991782 ; 0.147137
H 2.499682 ; 0.431784 ; -0.603110
****
332-1
Charge:1   Multiplicity:1 ()
E(Hartree):-175.847631   Zero-point:0.086243
Cartesian coordinates (Angstroms)
Ta 0.060099 ; -0.310138 ; 0.000017
C -1.117286 ; 1.475997 ; 0.000047
H -1.266673 ; 2.549127 ; 0.000449
C -1.862234 ; 0.395351 ; -0.000148
H -2.917284 ; 0.160585 ; -0.000250
H -0.109373 ; -1.196596 ; -1.513857
H -0.109315 ; -1.196935 ; 1.513708
C 1.814573 ; 1.048488 ; -0.000070
H 2.036328 ; 2.116233 ; -0.000662
H 2.282109 ; 0.611653 ; 0.898141
H 0.405191 ; 1.466093 ; 0.000551
H 2.281502 ; 0.610904 ; -0.898289
****
333-1
Charge:1   Multiplicity:1 ()
E(Hartree):-175.842432   Zero-point:0.086896
Cartesian coordinates (Angstroms)
Ta -0.143112 ; -0.166846 ; -0.079276
C 2.067844 ; 0.056002 ; 0.628208
H 2.914452 ; 0.290287 ; 1.262103
C 1.559832 ; 0.886915 ; -0.336092
H 1.911910 ; 1.886472 ; -0.552653
H -0.063087 ; -1.922511 ; 0.152731
H 0.951570 ; 0.386517 ; -1.498067
C -1.967058 ; 0.722033 ; 0.417968
H -2.497711 ; 0.060754 ; 1.118815
H -1.948900 ; 1.737586 ; 0.822864
H 1.745589 ; -0.994108 ; 0.754557
H -2.530323 ; 0.745079 ; -0.533727
****
334
Charge:1   Multiplicity:1 ()
E(Hartree):-175.834032   Zero-point:0.087902
Cartesian coordinates (Angstroms)
Ta -0.148418 ; -0.186428 ; -0.000062
C 1.698932 ; 0.387246 ; 0.780211
H 2.331148 ; -0.381838 ; 1.226245
H 1.829832 ; 1.357329 ; 1.252755
C -1.681896 ; 1.039344 ; 0.000311
H -2.127439 ; 1.455707 ; 0.908183
H -2.128063 ; 1.456124 ; -0.907047
H -1.522928 ; -1.509512 ; -0.000408
H -2.004160 ; -0.634323 ; 0.000023
C 1.698878 ; 0.387960 ; -0.779987
H 2.331630 ; -0.380182 ; -1.226834
H 1.829000 ; 1.358619 ; -1.251589
****
337
Charge:1   Multiplicity:1 (0.0000)
E(Hartree):-175.778132   Zero-point:0.086516
Cartesian coordinates (Angstroms)
Ta -0.358017 ; -0.312909 ; 0.817099

C 1.240831 ; 0.649159 ; 1.301278
H 1.815669 ; 1.211801 ; 2.026627
C 1.288169 ; 0.685503 ; -0.117080
H 1.329571 ; 1.541532 ; -0.781310
C -1.637703 ; 0.979362 ; -0.242900
H -1.742387 ; 0.466571 ; -1.214856
H 0.123486 ; -1.638911 ; -0.227935
H -1.421239 ; 2.031178 ; -0.411398
H -2.611286 ; 0.882700 ; 0.274195
H 2.494233 ; 0.141115 ; -0.576245
H 1.807032 ; -0.287955 ; -0.845771
****
344
Charge:1 Multiplicity:1 ()
E(Hartree):-175.842432 Zero-point:0.086894
Cartesian coordinates (Angstroms)
Ta 0.143101 ; -0.166826 ; 0.079245
C -2.067938 ; 0.055995 ; -0.628167
H -2.914757 ; 0.290422 ; -1.261739
H -1.745665 ; -0.994088 ; -0.754786
C -1.559798 ; 0.886855 ; 0.336112
H -0.951105 ; 0.386323 ; 1.498193
H -1.911907 ; 1.886357 ; 0.552861
C 1.967162 ; 0.721927 ; -0.417918
H 1.948935 ; 1.737359 ; -0.823123
H 0.063243 ; -1.922628 ; -0.151928
H 2.498098 ; 0.060524 ; -1.118423
H 2.530238 ; 0.745395 ; 0.533876
****
346
Charge:1 Multiplicity:1 ()
E(Hartree):-175.863261 Zero-point:0.085398
Cartesian coordinates (Angstroms)
Ta -0.129340 ; -0.245818 ; -0.063451
C 1.887162 ; 0.336868 ; 0.511168
H 2.783575 ; 0.244269 ; 1.108530
C 1.404778 ; 1.077240 ; -0.440196
H 1.685521 ; 1.919367 ; -1.055549
H -0.219186 ; -0.801431 ; -1.732843
H 1.084914 ; -0.879757 ; 1.166860
H 0.285971 ; -1.475916 ; 1.269539
C -1.660290 ; 1.101027 ; 0.372553
H -2.048656 ; 1.381147 ; -0.622396
H -2.418465 ; 0.466786 ; 0.870166
H -1.501767 ; 1.999429 ; 0.966488
****
352
Charge:1 Multiplicity:1 ()
E(Hartree):-175.811386 Zero-point:0.089222
Cartesian coordinates (Angstroms)
Ta -0.423313 ; -0.016302 ; -0.016446
H -1.721303 ; 0.314803 ; -1.181270
C 1.289950 ; -1.434467 ; 0.255402
H 0.920763 ; -1.427600 ; 1.288527
H -0.771453 ; 0.036503 ; 1.717774
H 1.860981 ; -2.329290 ; 0.038339
C 1.464463 ; -0.261476 ; -0.502227
H 2.088790 ; -0.178817 ; -1.381723
C 1.274749 ; 1.553168 ; 0.218544
H 1.066787 ; 1.824247 ; 1.255864
H 2.362311 ; 1.498346 ; 0.121214
H 0.920011 ; 2.308518 ; -0.488472

****

$TaC_3H_{10}^+$

Minima

121
Charge:1 Multiplicity:3 (2.0000)
E(Hartree):-177.098024 Zero-point:0.113175
Cartesian coordinates (Angstroms)
Ta 0.054496 ; -0.000026 ; -0.041355
C 1.169765 ; 1.764271 ; 0.110249
H 2.241188 ; 1.574498 ; 0.231146
H 1.015060 ; 2.357877 ; -0.802959
H 0.813515 ; 2.348137 ; 0.971255
C 1.170973 ; -1.763576 ; 0.110284
H 0.815833 ; -2.347896 ; 0.971402
H 1.017011 ; -2.357447 ; -0.802858
H 2.242170 ; -1.572170 ; 0.230908
C -2.602412 ; -0.000544 ; 0.108311
H -3.577112 ; -0.000111 ; -0.382794
H -2.518794 ; -0.903065 ; 0.714372
H -1.938893 ; -0.000953 ; -0.798786
H -2.518124 ; 0.902111 ; 0.714195
****
122
Charge:1 Multiplicity:1 (0.0111)
E(Hartree):-177.089902 Zero-point:0.112689
Cartesian coordinates (Angstroms)
Ta 0.048550 ; 0.000030 ; -0.028528
C 1.181711 ; 1.747789 ; 0.076321
H 2.255365 ; 1.555576 ; 0.168581
H 1.000848 ; 2.324569 ; -0.843137
H 0.849944 ; 2.340174 ; 0.941098
C 1.180455 ; -1.748515 ; 0.076310
H 0.847143 ; -2.340855 ; 0.940554
H 0.999319 ; -2.324624 ; -0.843540
H 2.254306 ; -1.558009 ; 0.169444
C -2.576377 ; 0.000522 ; 0.078779
H -3.586269 ; 0.001271 ; -0.335130
H -2.455721 ; -0.904146 ; 0.678834
H -1.969333 ; -0.000085 ; -0.861302
H -2.454473 ; 0.905141 ; 0.678674
****
123
Charge:1 Multiplicity:1 (0.0000)
E(Hartree):-177.05906 Zero-point:0.115071
Cartesian coordinates (Angstroms)
Ta 0.000039 ; 0.046895 ; 0.006543
C -0.000016 ; -1.800946 ; -0.085211
H -0.000043 ; -1.655801 ; 1.064145
H 0.000191 ; -2.834874 ; -0.402485
C 2.271167 ; 0.602888 ; -0.032025
H 3.282063 ; 0.197179 ; -0.014750
H 1.834915 ; 0.149417 ; -0.976264
H 1.844332 ; 0.282038 ; 0.969681
H 2.316369 ; 1.690899 ; -0.100533
C -2.271510 ; 0.602798 ; -0.032028
H -1.834821 ; 0.149694 ; -0.976208
H -2.317446 ; 1.690729 ; -0.100823
H -3.281987 ; 0.196103 ; -0.014607
H -1.844283 ; 0.282831 ; 0.969782

****
125
Charge:1   Multiplicity:3 (2.0009)
E(Hartree):-177.040175   Zero-point:0.120468
Cartesian coordinates (Angstroms)
C   -2.216801 ; -0.754233 ; -0.005780
H   -2.955700 ; -1.155076 ; 0.687792
H   -1.297417 ; -1.315992 ; 0.388587
H   -2.409263 ; -1.152799 ; -1.000642
C   -2.210953 ; 0.762673 ; 0.043841
H   -1.300151 ; 1.318320 ; -0.378190
H   -2.967025 ; 1.168699 ; -0.627875
H   -2.372137 ; 1.163121 ; 1.043622
Ta   0.189388 ; -0.001839 ; -0.009239
C   2.591957 ; 0.007429 ; 0.040252
H   3.343488 ; -0.170451 ; -0.728705
H   2.042008 ; -0.971910 ; 0.127900
H   3.060594 ; 0.225917 ; 0.998125
H   2.045042 ; 0.929225 ; -0.306063
****
128
Charge:1   Multiplicity:3 (2.0000)
E(Hartree):-177.05391   Zero-point:0.108620
Cartesian coordinates (Angstroms)
Ta   0.078513 ; -0.044478 ; -0.094777
C   1.610951 ; -1.366793 ; 0.388462
H   1.608951 ; -2.409982 ; 0.063962
H   2.508052 ; -1.114614 ; 0.956494
C   0.602425 ; 1.955429 ; 0.231203
H   0.190551 ; 2.617931 ; -0.539763
H   0.288367 ; 2.310256 ; 1.222472
H   -0.338689 ; -0.331255 ; -1.770796
H   1.700337 ; 2.006727 ; 0.180846
C   -2.480879 ; -0.336443 ; 0.324209
H   -3.470458 ; -0.324760 ; -0.136585
H   -2.211375 ; 0.718176 ; 0.483737
H   -2.509612 ; -0.881566 ; 1.263432
H   -1.892540 ; -0.857210 ; -0.468343
****
127
Charge:1   Multiplicity:1 (0.0000)
E(Hartree):-177.100357   Zero-point:0.109285
Cartesian coordinates (Angstroms)
Ta   0.055893 ; -0.022947 ; -0.158054
C   0.426688 ; -1.718499 ; 0.589100
H   -0.082528 ; -2.385385 ; -0.135897
H   0.971376 ; -2.239861 ; 1.368211
C   1.607108 ; 1.209950 ; 0.493545
H   1.319942 ; 2.269027 ; 0.434955
H   2.060280 ; 1.006276 ; 1.465206
H   0.258277 ; -0.366624 ; -1.872604
H   2.359392 ; 1.012994 ; -0.294321
C   -2.294400 ; 0.556115 ; 0.396592
H   -2.057945 ; 1.585477 ; 0.670630
H   -1.714588 ; -0.233966 ; 0.955623
H   -3.294211 ; 0.321626 ; 0.766426
H   -2.336585 ; 0.420154 ; -0.695726
****
132
Charge:1   Multiplicity:1 (0.0000)
E(Hartree):-177.150271   Zero-point:0.108837
Cartesian coordinates (Angstroms)
Ta   0.000044 ; 0.000036 ; -0.153149
C   -0.442359 ; -1.937840 ; 0.467863
H   -0.563350 ; -2.462836 ; -0.498761
H   0.364576 ; -2.423151 ; 1.026553
H   -1.379394 ; -2.023690 ; 1.027753
C   -1.457328 ; 1.351757 ; 0.467948
H   -1.853002 ; 1.717342 ; -0.498709
H   -2.279994 ; 0.895361 ; 1.028113
H   -1.063552 ; 2.207330 ; 1.026338
C   1.899396 ; 0.585835 ; 0.468077
H   2.441950 ; -0.181809 ; 1.029546
H   2.414885 ; 0.741700 ; -0.498703
H   1.916206 ; 1.528349 ; 1.025032
H   0.000185 ; 0.000232 ; -1.910611
****

$TaC_4H_6^+$

Minima

116
Charge:1   Multiplicity:1 (0.0228)
E(Hartree):-212.752684   Zero-point:0.085200
Cartesian coordinates (Angstroms)
C   -0.911340 ; -1.741937 ; 0.113002
H   -0.946257 ; -1.947324 ; 1.186308
H   -0.826933 ; -2.631353 ; -0.501417
C   -1.586295 ; -0.590008 ; -0.395369
H   -1.772006 ; -0.517753 ; -1.466202
C   -1.584481 ; 0.596146 ; 0.394511
H   -1.770945 ; 0.523910 ; 1.465151
C   -0.903728 ; 1.745441 ; -0.112399
H   -0.937995 ; 1.953141 ; -1.185154
H   -0.815802 ; 2.633491 ; 0.503582
Ta   0.506644 ; -0.000986 ; -0.000010
****
117
Charge:1   Multiplicity:3 (2.0001)
E(Hartree):-212.704922   Zero-point:0.074750
Cartesian coordinates (Angstroms)
Ta   -0.117902 ; 0.000005 ; -0.225295
C   1.456802 ; -0.000856 ; 1.052707
H   1.844651 ; -0.000744 ; 2.062218
C   1.924734 ; -0.001530 ; -0.193538
H   2.915512 ; -0.002185 ; -0.632858
C   -0.963292 ; -1.741406 ; 0.553200
H   -0.487718 ; -2.717948 ; 0.628738
H   -1.967600 ; -1.732475 ; 0.984746
C   -0.960187 ; 1.743309 ; 0.552861
H   -0.482355 ; 2.718861 ; 0.626821
H   -1.964003 ; 1.737019 ; 0.985470
****
118
Charge:1   Multiplicity:1 (0.0000)
E(Hartree):-212.73954   Zero-point:0.080599
Cartesian coordinates (Angstroms)
Ta   -0.046981 ; -0.266157 ; -0.000019
C   -1.781530 ; 0.538120 ; 0.677596
H   -2.479798 ; 0.970933 ; 1.384649
C   -1.781469 ; 0.538408 ; -0.677555

H   -2.479914 ; 0.971828 ; -1.384078
C   1.475014 ; 1.295769 ; 0.000130
H   1.429004 ; 1.905095 ; -0.901197
H   1.429818 ; 1.905731 ; 0.901023
C   2.134834 ; 0.013119 ; 0.000031
H   2.624423 ; -0.318330 ; -0.915778
H   2.624961 ; -0.318308 ; 0.915533
****
115
Charge:1   Multiplicity:3 (2.0000)
E(Hartree):-212.761755   Zero-point:0.085503
Cartesian coordinates (Angstroms)
C   -0.911929 ; -1.743663 ; 0.112362
H   -0.942160 ; -1.946512 ; 1.186292
H   -0.828047 ; -2.634260 ; -0.500276
C   -1.589477 ; -0.593198 ; -0.394200
H   -1.769379 ; -0.520308 ; -1.465574
C   -1.589694 ; 0.592372 ; 0.393920
H   -1.768639 ; 0.518652 ; 1.465457
C   -0.913041 ; 1.743352 ; -0.111997
H   -0.943690 ; 1.947984 ; -1.185490
H   -0.828584 ; 2.632959 ; 0.502059
Ta   0.508292 ; 0.000114 ; -0.000041
****
119
Charge:1   Multiplicity:3 (2.0000)
E(Hartree):-212.751233   Zero-point:0.081420
Cartesian coordinates (Angstroms)
Ta   -0.105732 ; -0.270697 ; -0.093615
C   -1.825092 ; 0.291709 ; 0.792218
H   -2.573342 ; 0.290200 ; 1.575315
C   -1.624750 ; 1.028787 ; -0.311433
H   -2.128867 ; 1.859066 ; -0.790570
C   1.768819 ; 1.192234 ; 0.001504
H   1.834578 ; 1.524200 ; -1.033910
H   1.500539 ; 1.956252 ; 0.723641
C   2.318532 ; -0.005590 ; 0.392241
H   2.799655 ; -0.663977 ; -0.328084
H   2.460823 ; -0.247673 ; 1.440338
****
120
Charge:1   Multiplicity:1 (0.0869)
E(Hartree):-212.704228   Zero-point:0.074537
Cartesian coordinates (Angstroms)
Ta   -0.121266 ; 0.000000 ; -0.227996
C   1.455777 ; -0.000007 ; 1.057063
H   1.838350 ; -0.000004 ; 2.068269
C   1.922187 ; 0.000009 ; -0.187118
H   2.910223 ; 0.000035 ; -0.632763
C   -0.948280 ; -1.742280 ; 0.558403
H   -0.491712 ; -2.731198 ; 0.549610
H   -1.900516 ; -1.727373 ; 1.094220
C   -0.948298 ; 1.742272 ; 0.558406
H   -0.491696 ; 2.731176 ; 0.549614
H   -1.900516 ; 1.727394 ; 1.094253
****

$TaC_4H_8^+$

Minima

031
Charge:1   Multiplicity:3 (2.0001)
E(Hartree):-213.930619   Zero-point:0.095281
Cartesian coordinates (Angstroms)
C   -1.890578 ; -0.000640 ; -0.611911
H   -2.305395 ; -0.909906 ; -1.045083
H   -2.305945 ; 0.908146 ; -1.045562
C   -1.708039 ; -0.000248 ; 0.902754
H   -1.994470 ; 0.906808 ; 1.432625
H   -1.995011 ; -0.906645 ; 1.433401
Ta   0.136188 ; -0.000002 ; -0.077300
C   1.184462 ; 1.788554 ; 0.175436
H   2.274680 ; 1.821279 ; 0.236660
H   0.737952 ; 2.779147 ; 0.271036
C   1.185834 ; -1.787756 ; 0.175633
H   2.276066 ; -1.819327 ; 0.237319
H   0.740338 ; -2.778818 ; 0.271048
****
032
Charge:1   Multiplicity:5 (6.0001)
E(Hartree):-213.85195   Zero-point:0.088609
Cartesian coordinates (Angstroms)
C   -1.273058 ; -0.663565 ; 1.522059
H   -2.160556 ; -0.109071 ; 1.832990
H   -1.160457 ; -1.608290 ; 2.055379
C   1.132218 ; -1.610374 ; -0.708008
H   2.013933 ; -1.986839 ; -0.185845
H   0.946586 ; -2.131883 ; -1.647778
Ta   0.000005 ; 0.000113 ; 0.000208
C   1.273193 ; 1.479424 ; 0.754161
H   2.213263 ; 1.751545 ; 0.270424
H   1.106513 ; 2.025046 ; 1.683755
C   -1.132389 ; 0.793467 ; -1.570105
H   -0.892570 ; 1.714603 ; -2.102656
H   -2.066863 ; 0.342961 ; -1.910081
****
030
Charge:1   Multiplicity:1 (0.0000)
E(Hartree):-213.951438   Zero-point:0.098597
Cartesian coordinates (Angstroms)
C   -2.209697 ; -0.000735 ; -0.681953
H   -2.303741 ; -0.925206 ; -1.246194
H   -2.303197 ; 0.925389 ; -1.243613
C   -2.210368 ; -0.002463 ; 0.681918
H   -2.304057 ; 0.922062 ; 1.246133
H   -2.304657 ; -0.928494 ; 1.243554
Ta   0.178052 ; -0.000390 ; -0.000040
C   1.315813 ; 1.574073 ; 0.000566
H   2.400418 ; 1.437370 ; 0.000774
H   1.034789 ; 2.628399 ; 0.000571
C   1.323830 ; -1.568924 ; -0.000159
H   2.406530 ; -1.415663 ; -0.000265
H   1.058682 ; -2.627094 ; -0.000267
****
105
Charge:1   Multiplicity:3 (2.0000)
E(Hartree):-213.956377   Zero-point:0.102069
Cartesian coordinates (Angstroms)
Ta   -0.062526 ; -0.000358 ; -0.020713
C   2.281973 ; 0.001046 ; 0.733179
H   2.381378 ; 0.920612 ; 1.303938
H   2.382364 ; -0.917738 ; 1.305047

C	2.361969 ; 0.000162 ; -0.634377
H	2.507852 ; -0.919572 ; -1.193258
H	2.506530 ; 0.919408 ; -1.194450
C	-1.959762 ; -0.802126 ; 0.041273
H	-2.417024 ; -1.234986 ; -0.848726
H	-2.369003 ; -1.232428 ; 0.954915
C	-1.958493 ; 0.803964 ; 0.040140
H	-2.366470 ; 1.236097 ; 0.953503
H	-2.415342 ; 1.236474 ; -0.850219
****
106
Charge:1 Multiplicity:1 (0.0000)
E(Hartree):-213.964907 Zero-point:0.100683
Cartesian coordinates (Angstroms)
Ta	0.000075 ; 0.000895 ; 0.000034
C	1.906491 ; -0.769896 ; 0.272119
H	2.354216 ; -0.870418 ; 1.261158
H	2.351861 ; -1.470619 ; -0.434636
C	1.909614 ; 0.765857 ; -0.271713
H	2.356967 ; 0.863661 ; -1.261232
H	2.359379 ; 1.464500 ; 0.434210
C	-1.909144 ; 0.270560 ; 0.766822
H	-2.355005 ; -0.436096 ; 1.467241
H	-2.357539 ; 1.259434 ; 0.865871
C	-1.908015 ; -0.273537 ; -0.767395
H	-2.356963 ; 0.430817 ; -1.468102
H	-2.352028 ; -1.264482 ; -0.866006
****
107
Charge:1 Multiplicity:3 (2.0000)
E(Hartree):-213.953872 Zero-point:0.106728
Cartesian coordinates (Angstroms)
Ta	-0.728413 ; -0.000042 ; -0.047519
C	1.099794 ; 0.769173 ; 0.597023
H	1.020729 ; 1.184120 ; 1.604717
C	1.099893 ; -0.769151 ; 0.596801
H	1.020487 ; -1.184318 ; 1.604395
C	2.024286 ; -1.562774 ; -0.309543
H	3.058489 ; -1.453276 ; 0.029238
H	1.777403 ; -2.623567 ; -0.272441
H	1.988397 ; -1.241199 ; -1.351025
C	2.023755 ; 1.563093 ; -0.309534
H	1.777149 ; 2.623913 ; -0.271529
H	3.058197 ; 1.453034 ; 0.028302
H	1.986928 ; 1.242287 ; -1.351244
****
108
Charge:1 Multiplicity:1 (0.0534)
E(Hartree):-213.95146 Zero-point:0.106771
Cartesian coordinates (Angstroms)
Ta	0.702945 ; 0.000002 ; 0.000000
C	-1.242135 ; 0.624642 ; 0.425660
H	-1.473480 ; 0.434651 ; 1.478231
C	-1.242131 ; -0.624648 ; -0.425662
H	-1.473483 ; -0.434659 ; -1.478232
C	-1.804855 ; -1.935864 ; 0.097633
H	-2.897416 ; -1.885840 ; 0.091563
H	-1.509280 ; -2.775447 ; -0.531484
H	-1.495299 ; -2.142473 ; 1.123128
C	-1.804875 ; 1.935851 ; -0.097632
H	-2.897435 ; 1.885814 ; -0.091563
H	-1.509310 ; 2.775436 ; 0.531488
H	-1.495320 ; 2.142467 ; -1.123126
****
109
Charge:1 Multiplicity:1 (0.0542)
E(Hartree):-213.950328 Zero-point:0.106769
Cartesian coordinates (Angstroms)
Ta	-0.727322 ; -0.000058 ; -0.047362
C	1.106922 ; 0.758537 ; 0.595080
H	0.992187 ; 1.190126 ; 1.593161
C	1.107062 ; -0.758483 ; 0.594769
H	0.991758 ; -1.190285 ; 1.592721
C	2.019082 ; -1.571003 ; -0.308683
H	3.048457 ; -1.497094 ; 0.053719
H	1.739422 ; -2.624056 ; -0.291250
H	2.014516 ; -1.230451 ; -1.343895
C	2.018328 ; 1.571420 ; -0.308716
H	1.738416 ; 2.624403 ; -0.291027
H	3.047878 ; 1.497751 ; 0.053257
H	2.013480 ; 1.230999 ; -1.343965
****
110
Charge:1 Multiplicity:3 (2.0000)
E(Hartree):-213.955218 Zero-point:0.106690
Cartesian coordinates (Angstroms)
Ta	0.704419 ; 0.000002 ; 0.000000
C	-1.235569 ; 0.629615 ; 0.436152
H	-1.501115 ; 0.422702 ; 1.476825
C	-1.235565 ; -0.629621 ; -0.436154
H	-1.501118 ; -0.422711 ; -1.476825
C	-1.811689 ; -1.928903 ; 0.098695
H	-2.902578 ; -1.858102 ; 0.134531
H	-1.556136 ; -2.770415 ; -0.545284
H	-1.467821 ; -2.152562 ; 1.110491
C	-1.811710 ; 1.928889 ; -0.098693
H	-2.902598 ; 1.858073 ; -0.134531
H	-1.556168 ; 2.770403 ; 0.545288
H	-1.467844 ; 2.152557 ; -1.110489
****
111
Charge: Multiplicity: (0.0486)
E(Hartree):-213.949526 Zero-point:0.107141
Cartesian coordinates (Angstroms)
Ta	-0.877599 ; -0.091860 ; -0.011208
C	0.584483 ; 1.374692 ; -0.151506
H	0.428089 ; 2.259932 ; 0.463780
C	1.135382 ; 0.133241 ; 0.496804
H	1.251572 ; 0.203077 ; 1.582020
C	2.228577 ; -0.686197 ; -0.183057
H	2.245364 ; -1.688309 ; 0.251416
H	2.007057 ; -0.802468 ; -1.247749
H	0.928205 ; 1.611151 ; -1.158751
C	3.606873 ; -0.031593 ; -0.018028
H	3.873013 ; 0.062971 ; 1.035630
H	4.366177 ; -0.643307 ; -0.505882
H	3.633338 ; 0.961891 ; -0.467525
****
112
Charge: Multiplicity: (2.0000)
E(Hartree):-213.953275 Zero-point:0.107109
Cartesian coordinates (Angstroms)
Ta	-0.877088 ; -0.090152 ; -0.013719
C	0.589977 ; 1.375043 ; -0.146590

H 0.460543 ; 2.260763 ; 0.473309
C 1.130669 ; 0.118235 ; 0.520810
H 1.263570 ; 0.214810 ; 1.601486
C 2.223128 ; -0.697118 ; -0.162450
H 2.259751 ; -1.690894 ; 0.289567
H 1.982702 ; -0.839816 ; -1.221123
H 0.971624 ; 1.602717 ; -1.141816
C 3.597390 ; -0.026034 ; -0.039700
H 3.883415 ; 0.091604 ; 1.006474
H 4.354204 ; -0.638082 ; -0.531108
H 3.604598 ; 0.959247 ; -0.507749
****
113
Charge:1 Multiplicity:3 (2.0000)
E(Hartree):-213.967923 Zero-point:0.101154
Cartesian coordinates (Angstroms)
Ta 0.284175 ; 0.000348 ; -0.235922
C -2.006785 ; -0.000998 ; 0.527975
H -2.903500 ; -0.000226 ; -0.084420
H -2.254749 ; -0.001526 ; 1.583733
C -1.202981 ; 1.343462 ; 0.222135
H -1.152005 ; 2.020595 ; 1.069329
H -1.574610 ; 1.855342 ; -0.672565
C -1.202855 ; -1.344077 ; 0.219851
H -1.574403 ; -1.855211 ; -0.675283
H -1.150168 ; -2.022397 ; 1.066015
C 1.918129 ; -0.001417 ; 1.051775
H 1.888165 ; -0.002900 ; 2.140064
H 2.943440 ; -0.000931 ; 0.665048
****
114
Charge:1 Multiplicity:1 (0.0000)
E(Hartree):-214.00108 Zero-point:0.102530
Cartesian coordinates (Angstroms)
Ta 0.343758 ; -0.000135 ; -0.253245
C -1.951625 ; 0.000098 ; 0.515096
H -2.885451 ; -0.000035 ; -0.040283
H -2.132540 ; 0.000252 ; 1.583315
C -1.181077 ; 1.336249 ; 0.129051
H -1.146613 ; 2.069936 ; 0.926107
H -1.566779 ; 1.775950 ; -0.797330
C -1.180922 ; -1.336079 ; 0.129672
H -1.566456 ; -1.776403 ; -0.796533
H -1.145976 ; -2.069432 ; 0.927040
C 1.341392 ; -0.000133 ; 1.370388
H 1.593470 ; -0.911187 ; 1.915968
H 1.589370 ; 0.919992 ; 1.903332
****
141
Charge:1 Multiplicity:1 (0.0115)
E(Hartree):-213.975518 Zero-point:0.106882
Cartesian coordinates (Angstroms)
Ta 0.547626 ; -0.276199 ; -0.002468
C -1.086867 ; 1.453678 ; 0.161884
H -1.724248 ; 1.744217 ; -0.667818
H -1.314146 ; 2.020211 ; 1.059336
C 0.415336 ; 1.751954 ; -0.238693
H 0.948849 ; 2.365167 ; 0.488054
H 0.529633 ; 2.132045 ; -1.254456
C -1.429356 ; -0.091418 ; 0.472515
H -1.530226 ; -0.232213 ; 1.556207
C -2.595452 ; -0.679013 ; -0.311334
H -2.739821 ; -1.728731 ; -0.057189
H -3.516836 ; -0.147413 ; -0.060267
H -2.451857 ; -0.601987 ; -1.389920
****
142
Charge:1 Multiplicity:3 (2.0000)
E(Hartree):-213.982208 Zero-point:0.107117
Cartesian coordinates (Angstroms)
Ta 0.549012 ; -0.274436 ; -0.001544
C -1.096000 ; 1.448344 ; 0.169248
H -1.744690 ; 1.740221 ; -0.651472
H -1.314882 ; 2.016823 ; 1.067780
C 0.398917 ; 1.757798 ; -0.250474
H 0.929588 ; 2.388443 ; 0.463236
H 0.495665 ; 2.138103 ; -1.268053
C -1.433805 ; -0.092913 ; 0.487603
H -1.568936 ; -0.226110 ; 1.566971
C -2.575622 ; -0.694555 ; -0.322825
H -2.723509 ; -1.742725 ; -0.063849
H -3.507123 ; -0.165902 ; -0.104335
H -2.404909 ; -0.627087 ; -1.398901
****
143
Charge:1 Multiplicity:1 (0.0000)
E(Hartree):-213.957329 Zero-point:0.106039
Cartesian coordinates (Angstroms)
C -2.153360 ; 0.726158 ; 0.256455
C -2.153062 ; -0.726878 ; -0.255940
C -0.816878 ; -1.417635 ; 0.119211
C -0.817846 ; 1.417747 ; -0.119956
H -3.000217 ; 1.286247 ; -0.143780
H -2.249173 ; 0.741837 ; 1.343250
H -2.249446 ; -0.742392 ; -1.342700
H -2.999409 ; -1.287509 ; 0.144592
H -0.757600 ; -1.653982 ; 1.202013
H -0.578135 ; -2.314243 ; -0.466573
H -0.580081 ; 2.315546 ; 0.464265
H -0.759547 ; 1.652380 ; -1.203150
Ta 0.668774 ; 0.000079 ; 0.000047
****
144
Charge:1 Multiplicity:3 (2.0000)
E(Hartree):-213.976439 Zero-point:0.107129
Cartesian coordinates (Angstroms)
C -2.064607 ; 0.723611 ; 0.274365
C -2.063952 ; -0.723806 ; -0.275246
C -0.786662 ; -1.515906 ; 0.125222
C -0.786662 ; 1.515921 ; -0.123623
H -2.948398 ; 1.254968 ; -0.083048
H -2.134443 ; 0.699535 ; 1.364007
H -2.131520 ; -0.699642 ; -1.365055
H -2.948462 ; -1.255315 ; 0.080174
H -0.819221 ; -1.884149 ; 1.161475
H -0.582728 ; -2.364911 ; -0.537064
H -0.582946 ; 2.363667 ; 0.540359
H -0.818098 ; 1.886140 ; -1.159236
Ta 0.646262 ; 0.000011 ; -0.000081
****
161
Charge:1 Multiplicity:1 (0.0000)
E(Hartree):-213.9882 Zero-point:0.099498
Cartesian coordinates (Angstroms)

Ta -0.717360 ; -0.000072 ; -0.036047
C 1.162160 ; 0.675693 ; -0.031470
C 1.162175 ; -0.675832 ; -0.031375
H -0.823613 ; 1.075501 ; 1.316951
H -0.823726 ; -1.075340 ; 1.317196
C 2.140189 ; 1.786114 ; 0.009014
H 2.324852 ; 2.046069 ; 1.055746
H 3.093000 ; 1.492324 ; -0.435787
H 1.771034 ; 2.683554 ; -0.486788
C 2.140962 ; -1.785610 ; 0.009006
H 3.091073 ; -1.493108 ; -0.442421
H 2.331838 ; -2.040252 ; 1.055940
H 1.769881 ; -2.685680 ; -0.480488
****
262
Charge:1 Multiplicity:1 (0.0000)
E(Hartree):-214.007284 Zero-point:0.099782
Cartesian coordinates (Angstroms)
Ta 0.062463 ; 0.000008 ; -0.311859
C -1.665598 ; -0.659225 ; 0.526698
H -2.294844 ; -1.473514 ; 0.863592
C -1.663667 ; 0.662169 ; 0.528276
H -2.290285 ; 1.477713 ; 0.867003
C 1.050857 ; -1.514393 ; 0.781923
H 0.712850 ; -2.392307 ; 0.199637
H 2.129764 ; -1.379141 ; 0.632821
H 0.836809 ; -1.698797 ; 1.831286
C 1.054203 ; 1.511836 ; 0.781770
H 2.132828 ; 1.374176 ; 0.632785
H 0.717790 ; 2.388462 ; 0.196167
H 0.840497 ; 1.700495 ; 1.830386
****
298-2
Charge:1 Multiplicity:1 (0.0000)
E(Hartree):-214.023808 Zero-point:0.096763
Cartesian coordinates (Angstroms)
Ta 0.221674 ; -0.065700 ; -0.212521
C -1.939977 ; 0.322221 ; 0.459834
H -2.954637 ; 0.435162 ; 0.837280
C -1.191505 ; 1.365695 ; 0.032354
H -1.496760 ; 2.400910 ; 0.001864
C -1.389616 ; -1.138908 ; 0.492445
H -1.468424 ; -1.569262 ; 1.491232
H -1.909714 ; -1.770328 ; -0.236269
C 1.904515 ; 0.237847 ; 0.992293
H 1.968185 ; -0.459186 ; 1.836589
H 2.037812 ; 1.260106 ; 1.359210
H 2.731620 ; 0.013652 ; 0.291064
H 0.609206 ; -0.236077 ; -1.928495
****
325
Charge:1 Multiplicity:1 ()
E(Hartree):-213.984307 Zero-point:0.098837
Cartesian coordinates (Angstroms)
Ta 0.002643 ; -0.101651 ; -0.217806
C -1.788642 ; 0.824230 ; -0.155791
H -2.792250 ; 1.055798 ; -0.489639
C -1.059665 ; 1.191977 ; 0.903233
H -1.194814 ; 1.818223 ; 1.775148
C -0.360028 ; -1.695583 ; 0.779320
H 0.446734 ; -2.423995 ; 0.576982
H -1.093148 ; -2.048784 ; 1.493094
C 2.350441 ; 0.707970 ; 0.323054
H 3.282262 ; 0.918432 ; 0.849953
H 1.650074 ; 0.467062 ; 1.155581
H 2.084472 ; 1.611915 ; -0.230853
H 2.571112 ; -0.149648 ; -0.329345
****
362
Charge:1 Multiplicity:1 ()
E(Hartree):-213.986601 Zero-point:0.098030
Cartesian coordinates (Angstroms)
Ta 0.203634 ; -0.052453 ; -0.228174
C -2.563925 ; 0.160830 ; 0.154864
H -3.622865 ; 0.009840 ; 0.351918
C -1.616674 ; -0.723284 ; 0.511167
H -1.876385 ; -1.640703 ; 1.028630
C 0.596992 ; 1.849654 ; 0.585176
H 0.107639 ; 1.980572 ; 1.554922
H 1.672693 ; 2.011248 ; 0.702762
H -2.342905 ; 1.101283 ; -0.353956
H 0.197058 ; 2.602123 ; -0.112630
C 1.482049 ; -1.149009 ; 0.664782
H 2.416120 ; -0.872990 ; 1.150655
H 1.192690 ; -2.191435 ; 0.838477
****
Transition states

363
Charge:1 Multiplicity:1 ()
E(Hartree):-213.954482 Zero-point:0.095702
Cartesian coordinates (Angstroms)
Ta -0.064596 ; -0.162728 ; -0.247434
C 1.242679 ; 1.159715 ; 0.786496
H 1.486740 ; 1.898282 ; 1.539870
C 1.893725 ; 0.432562 ; -0.097948
H 2.937601 ; 0.301433 ; -0.347278
C -1.676659 ; 1.288674 ; 0.315154
H -1.744533 ; 2.239751 ; 0.845182
H -1.871313 ; 1.532744 ; -0.748683
H -0.253811 ; 1.290791 ; 0.802869
H -2.463523 ; 0.640367 ; 0.704298
C -0.218707 ; -1.564839 ; 1.053295
H 0.434151 ; -1.994153 ; 1.805046
H -1.256064 ; -1.926761 ; 1.119398
****

$TaC_4H_{10}^+$

Minima

266-1
Charge:1 Multiplicity:1 (0.0000)
E(Hartree):-215.253364 Zero-point:0.119047
Cartesian coordinates (Angstroms)
Ta -0.114118 ; -0.000033 ; -0.000115
C 1.789658 ; 0.001204 ; -0.789185
H 2.192316 ; -0.904187 ; -1.240527
H 2.191234 ; 0.907077 ; -1.240525
C 1.789385 ; 0.001277 ; 0.789588
H 2.190724 ; 0.907196 ; 1.241044
H 2.191909 ; -0.904060 ; 1.241156
C -1.162161 ; 1.828759 ; 0.000169
H -2.250579 ; 1.705312 ; 0.000184

H -0.873631 ; 2.414888 ; 0.885244
H -0.873727 ; 2.416153 ; -0.884050
C -1.158889 ; -1.830680 ; 0.000233
H -0.869250 ; -2.416408 ; 0.885205
H -2.247565 ; -1.709713 ; -0.000080
H -0.868800 ; -2.417178 ; -0.884071
****
303
Charge:1 Multiplicity:1 (0.0000)
E(Hartree):-215.222378 Zero-point:0.120518
Cartesian coordinates (Angstroms)
Ta 0.247930 ; 0.000001 ; -0.123940
C -1.302026 ; -1.343212 ; 0.058575
H -1.553552 ; -1.755860 ; -0.928990
H -1.403517 ; -2.101941 ; 0.827091
C -1.302023 ; 1.343210 ; 0.058578
H -1.403514 ; 2.101941 ; 0.827091
H -1.553546 ; 1.755856 ; -0.928989
C 2.251762 ; -0.000005 ; 0.451231
H 2.703183 ; 0.897171 ; 0.877607
H 0.154654 ; -0.000003 ; 1.591798
H 2.478193 ; 0.000042 ; -0.663407
H 2.703195 ; -0.897211 ; 0.877532
C -2.117179 ; 0.000000 ; 0.348752
H -2.433536 ; -0.000001 ; 1.384674
H -2.973648 ; 0.000001 ; -0.319636
****
354
Charge:1 Multiplicity:1 ()
E(Hartree):-215.229678 Zero-point:0.117342
Cartesian coordinates (Angstroms)
Ta -0.011562 ; 0.086229 ; -0.133337
C 0.635994 ; -1.676543 ; 0.679411
H 0.539252 ; -2.526523 ; 1.342707
C 1.548709 ; -1.207445 ; -0.155424
H 2.531777 ; -1.477402 ; -0.515620
H 0.049837 ; -0.033796 ; -1.884291
C -2.495078 ; -0.182721 ; 0.242269
H -3.437228 ; -0.711223 ; 0.392521
H -2.613944 ; 0.844904 ; 0.591403
H -1.787362 ; -0.784481 ; 0.858936
H -2.300813 ; -0.232053 ; -0.844246
C 1.025064 ; 1.777288 ; 0.561426
H 1.661038 ; 2.044799 ; -0.300242
H 0.268374 ; 2.573404 ; 0.670431
H 1.644974 ; 1.744189 ; 1.455903
****
355-1
Charge:1 Multiplicity:1 ()
E(Hartree):-215.243465 Zero-point:0.116102
Cartesian coordinates (Angstroms)
Ta 0.213565 ; 0.000009 ; -0.222896
C -1.833486 ; 0.000285 ; -0.493560
H -2.461247 ; 0.000506 ; -1.380136
C -2.353364 ; -0.000118 ; 0.743095
H -1.738184 ; -0.000295 ; 1.647160
H -3.421721 ; -0.000252 ; 0.945724
C 0.832701 ; -1.711147 ; 0.806487
H 1.590130 ; -2.087357 ; 0.090923
H 1.341393 ; -1.499696 ; 1.752857
H 0.077241 ; -2.485127 ; 0.956535
C 0.832899 ; 1.710937 ; 0.806784
H 1.589808 ; 2.087677 ; 0.090950
H 0.077096 ; 2.484537 ; 0.957191
H 1.342016 ; 1.499545 ; 1.752921
H 1.140716 ; 0.000046 ; -1.719558
****
358
Charge:1 Multiplicity:1 ()
E(Hartree):-215.224466 Zero-point:0.119119
Cartesian coordinates (Angstroms)
Ta -0.257774 ; -0.081865 ; -0.188487
C 1.224915 ; 1.440416 ; -0.242438
H 1.766579 ; 1.409026 ; -1.192481
H -0.261316 ; -1.170322 ; -1.579340
H 1.073206 ; 2.450690 ; 0.122629
C 1.532158 ; 0.414109 ; 0.780494
H 1.545152 ; 0.708726 ; 1.823940
C 1.972491 ; -0.959156 ; 0.418969
H 2.686412 ; -1.051535 ; -0.399866
H 2.280779 ; -1.546988 ; 1.279795
H 1.085307 ; -1.620700 ; -0.013838
C -2.079516 ; 0.142833 ; 0.828166
H -2.422943 ; 1.185207 ; 0.745513
H -2.826230 ; -0.518402 ; 0.365779
H -2.009748 ; -0.098796 ; 1.896267
****
357
Charge:1 Multiplicity:1 ()
E(Hartree):-215.225468 Zero-point:0.119337
Cartesian coordinates (Angstroms)
Ta -0.331387 ; -0.193712 ; 0.123277
C 1.466287 ; 0.512415 ; -0.575778
H 1.451777 ; 1.249826 ; -1.379067
C 1.014565 ; 1.167714 ; 0.820824
H 0.821057 ; 2.237625 ; 0.767389
H 1.660324 ; 0.892569 ; 1.656275
H -0.372249 ; -1.810553 ; 0.842041
C -2.068744 ; 0.560723 ; -0.773207
H -2.881898 ; -0.173250 ; -0.695356
H -2.384229 ; 1.495413 ; -0.288957
H -1.887006 ; 0.781278 ; -1.834870
C 2.703277 ; -0.368632 ; -0.545324
H 2.867404 ; -0.823050 ; -1.522552
H 3.584756 ; 0.228216 ; -0.299113
H 2.639009 ; -1.170446 ; 0.195871
****
376
Charge:1 Multiplicity:1 ()
E(Hartree):-215.243465 Zero-point:0.116109
Cartesian coordinates (Angstroms)
Ta 0.212996 ; -0.000006 ; -0.222991
C -2.348290 ; 0.000315 ; 0.744922
H -3.415638 ; 0.000270 ; 0.952859
C -1.833807 ; 0.000402 ; -0.493959
H -2.464778 ; 0.000452 ; -1.378136
H 1.140558 ; -0.000062 ; -1.719453
C 0.832300 ; -1.711432 ; 0.806016
H 0.077345 ; -2.486070 ; 0.955068
H 1.590387 ; -2.086508 ; 0.090533
H -1.729071 ; 0.000304 ; 1.646420
H 1.340386 ; -1.500374 ; 1.752793
C 0.833243 ; 1.710922 ; 0.806236
H 1.590411 ; 2.086499 ; 0.090039

H 0.078476 ; 2.485491 ; 0.956696
H 1.342505 ; 1.499231 ; 1.752256
****
377
Charge:1 Multiplicity:1 ()
E(Hartree):-215.201389 Zero-point:0.113288
Cartesian coordinates (Angstroms)
Ta -0.073529 ; 0.000010 ; -0.248157
C 1.729409 ; 0.658436 ; 0.442926
H 2.386933 ; 1.475238 ; 0.712960
C 1.728713 ; -0.659898 ; 0.443314
H 2.385246 ; -1.477384 ; 0.713682
H -0.586028 ; -0.380684 ; -2.446786
H -0.585364 ; 0.382318 ; -2.446506
C -0.999635 ; -1.530079 ; 0.872381
H -2.085846 ; -1.394842 ; 0.794484
H -0.691706 ; -2.365864 ; 0.210363
H -0.723452 ; -1.785563 ; 1.891611
C -0.998037 ; 1.530984 ; 0.872637
H -0.689407 ; 2.366802 ; 0.211033
H -2.084413 ; 1.397016 ; 0.795207
H -0.721047 ; 1.785556 ; 1.891889
****
Transition states

225
Charge:1 Multiplicity:1 (0.0000)
E(Hartree):-215.194042 Zero-point:0.119559
Cartesian coordinates (Angstroms)
Ta -0.092637 ; 0.019525 ; 0.063029
C 1.484793 ; -1.279742 ; -0.130891
H 1.771064 ; -1.646810 ; 0.864550
H 1.584254 ; -2.063212 ; -0.874584
C 1.430254 ; 1.385324 ; -0.108122
H 1.497608 ; 2.184996 ; -0.838033
H 1.701282 ; 1.746452 ; 0.893762
C -2.164913 ; -0.017865 ; -0.515086
H -2.739301 ; 0.867902 ; -0.789168
H -1.159341 ; 0.010541 ; -1.439434
H -2.102136 ; -0.026277 ; 0.653317
H -2.702635 ; -0.921738 ; -0.804043
C 2.245334 ; 0.072174 ; -0.495171
H 2.485934 ; 0.086116 ; -1.551729
H 3.152033 ; 0.085586 ; 0.103964
****
356
Charge:1 Multiplicity:1 ()
E(Hartree):-215.207938 Zero-point:0.113636
Cartesian coordinates (Angstroms)
Ta 0.106878 ; 0.077081 ; -0.193895
C -0.320248 ; -1.923318 ; 0.038492
H -0.004738 ; -2.945368 ; -0.115146
C -1.332680 ; -1.311398 ; 0.602673
H -1.531501 ; 0.205650 ; 0.593977
H 0.296088 ; -0.042197 ; -1.946318
H -2.235494 ; -1.593753 ; 1.130475
C -1.410980 ; 1.637454 ; 0.357912
H -0.780198 ; 2.286739 ; 0.977648
H -1.453369 ; 2.045082 ; -0.666653
H -2.424050 ; 1.684344 ; 0.760271
C 1.956729 ; 0.241085 ; 0.791964
H 2.147478 ; -0.281519 ; 1.728582

H 2.641448 ; -0.158286 ; 0.020686
H 2.185351 ; 1.309461 ; 0.924562
****
359
Charge:1 Multiplicity:1 ()
E(Hartree):-215.200914 Zero-point:0.114743
Cartesian coordinates (Angstroms)
Ta 0.118630 ; -0.035611 ; -0.012468
C -1.700636 ; -0.816606 ; -0.459870
H -2.146678 ; -1.745581 ; -0.790706
C -2.233220 ; -0.005815 ; 0.506956
H -1.863347 ; 1.002091 ; 0.726030
H -3.147287 ; -0.240346 ; 1.039507
C 1.910252 ; -1.118944 ; 0.223987
H 2.637622 ; -0.756985 ; -0.517542
H 2.324371 ; -0.920116 ; 1.224183
H 1.808153 ; -2.202726 ; 0.107012
C 0.484243 ; 2.038942 ; -0.113146
H 1.386896 ; 2.125742 ; -0.737241
H -0.267465 ; 2.739622 ; -0.474051
H 0.767695 ; 2.319967 ; 0.916983
H -0.923768 ; -0.307498 ; -1.531598
****
361-1
Charge:1 Multiplicity:1 ()
E(Hartree):-215.173529 Zero-point:0.116340
Cartesian coordinates (Angstroms)
Ta 0.223671 ; -0.091259 ; -0.160180
C -1.701368 ; -1.246912 ; 0.184063
H -1.626332 ; -1.352928 ; -0.903986
H 0.148676 ; -0.182616 ; -1.936183
H -2.298826 ; -2.032468 ; 0.630267
C -1.475522 ; -0.035481 ; 0.849958
H -1.803997 ; 0.184502 ; 1.856364
C -1.279996 ; 1.695863 ; -0.067736
H -1.347650 ; 1.850689 ; -1.146763
H -2.293503 ; 1.796439 ; 0.328504
H -0.661934 ; 2.451716 ; 0.421573
C 2.131573 ; 0.152519 ; 0.698497
H 2.711742 ; 0.797406 ; 0.018957
H 2.167574 ; 0.583029 ; 1.704006
H 2.628134 ; -0.829816 ; 0.731732
****
378
Charge:1 Multiplicity:1 ()
E(Hartree):-215.200914 Zero-point:0.114743
Cartesian coordinates (Angstroms)
Ta 0.118637 ; -0.035562 ; -0.012482
C -2.233226 ; -0.005846 ; 0.506933
H -3.147269 ; -0.240477 ; 1.039482
H -1.863394 ; 1.002068 ; 0.726028
C -1.700572 ; -0.816657 ; -0.459852
H -0.923719 ; -0.307505 ; -1.531591
H -2.146551 ; -1.745659 ; -0.790693
C 1.910074 ; -1.119196 ; 0.224011
H 1.807740 ; -2.202856 ; 0.106050
H 2.323653 ; -0.921367 ; 1.224628
H 2.637960 ; -0.756789 ; -0.516775
C 0.484333 ; 2.038925 ; -0.113118
H 0.767883 ; 2.319594 ; 0.917123
H 1.386931 ; 2.125860 ; -0.737269
H -0.267378 ; 2.739791 ; -0.473661

****
379
Charge:1 Multiplicity:1 ()
E(Hartree):-215.222928 Zero-point:0.112870
Cartesian coordinates (Angstroms)
Ta -0.100192 ; 0.000018 ; -0.177553
C 2.084676 ; 0.000089 ; -0.088002
H 3.064262 ; 0.000222 ; -0.544284
C 1.542906 ; -0.000204 ; 1.088921
H 1.831631 ; -0.000436 ; 2.129448
H 1.215899 ; 0.000372 ; -1.477191
H 0.377754 ; 0.000430 ; -1.986842
C -1.086054 ; -1.745168 ; 0.466793
H -1.633622 ; -1.582342 ; 1.402209
H -1.830468 ; -1.892053 ; -0.340437
H -0.489799 ; -2.654410 ; 0.553689
C -1.086120 ; 1.745008 ; 0.467151
H -0.489542 ; 2.653816 ; 0.556262
H -1.828517 ; 1.893211 ; -0.341739
H -1.636003 ; 1.581552 ; 1.401080
****

$TaC_4H_{12}^+$

Minima

254
Charge:1 Multiplicity:1 (0.0000)
E(Hartree):-216.508948 Zero-point:0.136881
Cartesian coordinates (Angstroms)
Ta 0.000094 ; -0.000021 ; -0.000038
C 1.151427 ; 1.732705 ; -0.318845
H 1.217036 ; 2.325280 ; 0.601532
H 2.168024 ; 1.466707 ; -0.632360
H 0.700105 ; 2.355956 ; -1.100072
C -1.435300 ; -0.179989 ; -1.528674
H -1.427908 ; -1.193957 ; -1.946082
H -2.439253 ; 0.026395 ; -1.138727
H -1.225998 ; 0.529135 ; -2.338462
C -0.972229 ; 0.136541 ; 1.861449
H -2.036582 ; -0.106164 ; 1.757681
H -0.526721 ; -0.561632 ; 2.580203
H -0.886642 ; 1.151925 ; 2.266571
C 1.255524 ; -1.689087 ; -0.013716
H 1.055582 ; -2.323955 ; 0.857734
H 1.090025 ; -2.283573 ; -0.920224
H 2.308929 ; -1.385615 ; 0.013691
****
256
Charge:1 Multiplicity:3 (2.0000)
E(Hartree):-216.427817 Zero-point:0.143212
Cartesian coordinates (Angstroms)
Ta 0.166772 ; -0.000102 ; -0.000094
C 1.541506 ; 1.599969 ; 0.141583
H 2.189956 ; 1.662478 ; -0.739695
H 0.971414 ; 2.536635 ; 0.213901
H 2.166358 ; 1.513260 ; 1.038244
C 1.543137 ; -1.598839 ; -0.141060
H 2.190982 ; -1.659812 ; 0.740812
H 0.974504 ; -2.536379 ; -0.213141
H 2.168641 ; -1.511805 ; -1.037212
C -2.290533 ; 0.317084 ; -0.695983
H -2.353141 ; 1.405753 ; -0.670608
H -1.441658 ; 0.035949 ; -1.388575
H -3.131274 ; -0.063348 ; -1.276277
C -2.291078 ; -0.317481 ; 0.696112
H -1.441498 ; -0.038255 ; 1.388565
H -3.131100 ; 0.064572 ; 1.276360
H -2.355720 ; -1.406005 ; 0.670562
****
257
Charge:1 Multiplicity:1 (0.0000)
E(Hartree):-216.462078 Zero-point:0.137578
Cartesian coordinates (Angstroms)
Ta 0.163988 ; 0.000047 ; -0.027996
C 1.241668 ; 1.786416 ; 0.161367
H 2.308280 ; 1.665511 ; -0.055630
H 0.842035 ; 2.623278 ; -0.418646
H 1.143094 ; 2.035206 ; 1.237344
C 1.235017 ; -1.790409 ; 0.159971
H 1.134868 ; -2.037816 ; 1.236217
H 0.832128 ; -2.625986 ; -0.419568
H 2.302216 ; -1.674139 ; -0.056474
C -1.816157 ; 0.003337 ; -0.765292
H -2.101694 ; 0.896885 ; -1.317405
H 0.429233 ; -0.000666 ; -1.745000
H -2.101849 ; -0.888683 ; -1.319875
C -2.273370 ; 0.001106 ; 0.693024
H -1.432597 ; -0.003781 ; 1.463949
H -2.822232 ; 0.897689 ; 0.974562
H -2.827578 ; -0.893650 ; 0.969846
****
258
Charge:1 Multiplicity:1 (0.0000)
E(Hartree):-216.456494 Zero-point:0.137583
Cartesian coordinates (Angstroms)
Ta 0.040853 ; 0.000001 ; 0.004974
C 0.257588 ; 0.000170 ; 1.907643
H 0.365680 ; -0.911103 ; 2.498854
H 0.366346 ; 0.913000 ; 2.496451
C 1.010731 ; 1.747171 ; -0.682958
H 0.618992 ; 2.010478 ; -1.676367
H 0.907772 ; 2.612079 ; -0.022557
H 2.080562 ; 1.526362 ; -0.794861
C 1.008704 ; -1.748462 ; -0.682391
H 0.904337 ; -2.613083 ; -0.021820
H 0.616939 ; -2.011477 ; -1.675869
H 2.078877 ; -1.529158 ; -0.793956
C -2.433485 ; 0.000939 ; -0.380048
H -2.288166 ; 0.906886 ; -0.975346
H -1.936383 ; 0.000995 ; 0.637845
H -3.469029 ; 0.001842 ; -0.034495
H -2.289436 ; -0.905809 ; -0.974442
****
255
Charge:1 Multiplicity:3 (2.0000)
E(Hartree):-216.399355 Zero-point:0.135183
Cartesian coordinates (Angstroms)
Ta 0.068993 ; 0.000117 ; 0.076807
C -0.252232 ; 1.805614 ; -0.973360
H 0.730606 ; 2.123531 ; -1.351137
H -0.917456 ; 1.702867 ; -1.836065
H -0.628997 ; 2.603699 ; -0.319461
C 2.165356 ; -0.004761 ; 0.574213

H 2.384689 ; -0.905512 ; 1.156113
H 2.743144 ; -0.006723 ; -0.356054
H 2.388277 ; 0.896188 ; 1.154497
C -0.259487 ; -1.802904 ; -0.975607
H -0.640502 ; -2.601158 ; -0.324512
H -0.923119 ; -1.695148 ; -1.838988
H 0.722391 ; -2.124032 ; -1.353251
C -2.257349 ; 0.001142 ; 0.830113
H -2.197231 ; -0.926322 ; 1.396121
H -2.198994 ; 0.930190 ; 1.393642
H -2.877044 ; -0.000702 ; -0.059966
****
Transition states

380
Charge:1 Multiplicity:1 (0.0000)
E(Hartree):-216.442948 Zero-point:0.134640
Cartesian coordinates (Angstroms)
Ta -0.062757 ; 0.000015 ; 0.014720
C 0.808291 ; -0.000045 ; 1.759803
H 1.073761 ; -0.909530 ; 2.301902
H 1.755671 ; 0.000250 ; 0.336166
H 1.073411 ; 0.909468 ; 2.302041
C -1.170679 ; 1.775840 ; -0.301475
H -0.967994 ; 2.168112 ; -1.307759
H -2.243267 ; 1.549738 ; -0.240121
H -0.939204 ; 2.559634 ; 0.425714
C -1.169424 ; -1.776608 ; -0.301803
H -0.937708 ; -2.559714 ; 0.426071
H -2.242343 ; -1.551891 ; -0.241780
H -0.965083 ; -2.169258 ; -1.307609
C 1.938239 ; 0.000707 ; -1.085155
H 1.770618 ; -0.896037 ; -1.693056
H 1.769903 ; 0.897715 ; -1.692483
H 2.994946 ; 0.001083 ; -0.811889
****

$TaC_5H_{10}^+$

Minima

147
Charge:1 Multiplicity:1 (0.0000)
E(Hartree):-253.347927 Zero-point:0.130017
Cartesian coordinates (Angstroms)
Ta -0.124435 ; 0.000102 ; -0.000148
C 2.293854 ; -0.000306 ; 0.000041
H 2.912524 ; -0.000381 ; 0.891634
H 2.912048 ; -0.000268 ; -0.891887
C 1.443886 ; 1.363169 ; 0.000315
H 1.616628 ; 1.964920 ; -0.892403
H 1.615834 ; 1.964042 ; 0.893809
C 1.443521 ; -1.363406 ; 0.000173
H 1.615491 ; -1.964665 ; 0.893395
H 1.615697 ; -1.964977 ; -0.892792
C -2.039301 ; 0.000008 ; -0.794926
H -2.456030 ; -0.903980 ; -1.236773
H -2.456549 ; 0.903916 ; -1.236416
C -2.038809 ; -0.000239 ; 0.795592
H -2.454651 ; -0.904707 ; 1.237302
H -2.456127 ; 0.903277 ; 1.237761
****

151
Charge:1 Multiplicity:1 (0.0552)
E(Hartree):-253.28119 Zero-point:0.135354
Cartesian coordinates (Angstroms)
Ta 0.929416 ; -0.234854 ; 0.004595
C -0.635208 ; 1.085577 ; 0.414257
H -0.943766 ; 0.994637 ; 1.460110
C -1.095924 ; -0.061611 ; -0.454285
H -1.236964 ; 0.209788 ; -1.505277
C -0.628193 ; 2.515694 ; -0.100510
H -1.652431 ; 2.898860 ; -0.112857
H -0.038680 ; 3.168488 ; 0.543349
H -0.242774 ; 2.590302 ; -1.118476
C -2.116313 ; -1.077758 ; 0.055071
H -2.077020 ; -1.969203 ; -0.574860
H -1.860442 ; -1.394659 ; 1.070275
C -3.539359 ; -0.504379 ; 0.046243
H -4.243641 ; -1.256332 ; 0.403236
H -3.622779 ; 0.368525 ; 0.695434
H -3.838862 ; -0.211235 ; -0.961010
****

152
Charge:1 Multiplicity:3 (2.0000)
E(Hartree):-253.288782 Zero-point:0.123605
Cartesian coordinates (Angstroms)
Ta -0.240328 ; 0.000032 ; 0.025220
C 2.190975 ; 0.001091 ; -0.087877
H 2.808362 ; -0.890738 ; -0.109356
H 2.805598 ; 0.894842 ; -0.108648
C 1.385009 ; -0.000512 ; 1.299700
H 1.569396 ; 0.895016 ; 1.891594
H 1.568128 ; -0.898332 ; 1.888646
C 1.275761 ; 0.000284 ; -1.393152
H 1.400890 ; -0.896885 ; -1.996965
H 1.400764 ; 0.896783 ; -1.997944
C -1.364483 ; 1.763337 ; -0.016173
H -2.323249 ; 1.861328 ; -0.528181
H -1.057339 ; 2.691853 ; 0.467922
C -1.362675 ; -1.764352 ; -0.016833
H -2.320576 ; -1.862922 ; -0.530335
H -1.055536 ; -2.692349 ; 0.468183
****

149
Charge:1 Multiplicity:1 (0.0000)
E(Hartree):-253.302621 Zero-point:0.133513
Cartesian coordinates (Angstroms)
C -2.529710 ; 0.000042 ; 0.416152
C -2.006775 ; 1.290457 ; -0.224939
C -0.492703 ; 1.520497 ; -0.064970
C -0.492760 ; -1.520508 ; -0.065023
C -2.006840 ; -1.290432 ; -0.224875
H -0.077196 ; 2.277327 ; -0.752089
H -2.227073 ; 1.290072 ; -1.294660
H -2.531904 ; 2.148198 ; 0.201703
H -2.320909 ; 0.000064 ; 1.491189
H -3.618302 ; 0.000064 ; 0.325569
H -0.077344 ; -2.277400 ; -0.752118
H -0.220361 ; -1.797399 ; 0.991686
H -2.227245 ; -1.290144 ; -1.294574
H -2.531943 ; -2.148126 ; 0.201895
H -0.220366 ; 1.797434 ; 0.991734
Ta 0.838704 ; -0.000006 ; 0.011940

****
373-1
Charge:1 Multiplicity:3 (2.0000)
E(Hartree):-253.317913 Zero-point:0.134814
Cartesian coordinates (Angstroms)
C -2.476355 ; 0.000001 ; 0.414246
C -1.966334 ; 1.290346 ; -0.239045
C -0.469810 ; 1.615892 ; -0.018461
C -0.469812 ; -1.615889 ; -0.018465
C -1.966337 ; -1.290346 ; -0.239041
H -0.104940 ; 2.384650 ; -0.716175
H -2.150034 ; 1.248890 ; -1.315281
H -2.545525 ; 2.136982 ; 0.137864
H -2.250750 ; 0.000002 ; 1.486727
H -3.566350 ; 0.000002 ; 0.343799
H -0.104942 ; -2.384648 ; -0.716177
H -0.280520 ; -1.992655 ; 1.010085
H -2.150043 ; -1.248898 ; -1.315276
H -2.545525 ; -2.136981 ; 0.137877
H -0.280526 ; 1.992661 ; 1.010087
Ta 0.822891 ; 0.000000 ; 0.007412
****
150
Charge:1 Multiplicity:1 (0.0000)
E(Hartree):-253.325279 Zero-point:0.130622
Cartesian coordinates (Angstroms)
C -2.108156 ; 0.810247 ; 0.488474
C -2.215615 ; -0.661451 ; 0.024802
C -0.933799 ; -1.477958 ; 0.350594
C -0.879408 ; 1.520760 ; -0.139412
H -3.016830 ; 1.354103 ; 0.223383
H -2.019427 ; 0.847431 ; 1.574886
H -2.404093 ; -0.688347 ; -1.053593
H -3.079345 ; -1.141129 ; 0.489111
H -0.870470 ; -1.783954 ; 1.394910
H -0.841807 ; -2.378968 ; -0.271753
H -0.607068 ; 2.457217 ; 0.344089
H -1.057745 ; 1.727167 ; -1.214686
Ta 0.512061 ; -0.047630 ; -0.237164
C 1.598223 ; 0.227134 ; 1.303395
H 1.806850 ; -0.615193 ; 1.968981
H 1.942041 ; 1.186293 ; 1.690503
****
153
Charge:1 Multiplicity:3 (2.0000)
E(Hartree):-253.332796 Zero-point:0.131312
Cartesian coordinates (Angstroms)
Ta 0.073447 ; 0.000138 ; -0.003152
C -2.323641 ; -0.000338 ; 0.006718
H -2.941720 ; -0.000761 ; -0.884851
H -2.933895 ; -0.000507 ; 0.903621
C -1.478507 ; 1.361739 ; 0.002755
H -1.644779 ; 1.959760 ; 0.898241
H -1.649882 ; 1.957501 ; -0.893438
C -1.477723 ; -1.362227 ; 0.003132
H -1.648975 ; -1.958283 ; -0.892881
H -1.643779 ; -1.959972 ; 0.898844
C 2.388049 ; 0.000214 ; 0.695092
H 2.506915 ; -0.919609 ; 1.261169
H 2.507990 ; 0.920518 ; 1.260135
C 2.398156 ; -0.000539 ; -0.679891
H 2.523664 ; -0.921019 ; -1.243298
H 2.524819 ; 0.919179 ; -1.244263
****
154
Charge:1 Multiplicity:3 (2.0000)
E(Hartree):-253.295187 Zero-point:0.129083
Cartesian coordinates (Angstroms)
C -2.180042 ; 0.757824 ; 0.472986
C -2.152687 ; -0.690051 ; -0.075385
C -0.920609 ; -1.492971 ; 0.441806
C -0.904279 ; 1.544971 ; 0.067696
H -3.070384 ; 1.275520 ; 0.112186
H -2.242323 ; 0.727141 ; 1.561791
H -2.146399 ; -0.667710 ; -1.176029
H -3.071474 ; -1.217675 ; 0.187149
H -0.950756 ; -1.668789 ; 1.519062
H -0.792389 ; -2.451726 ; -0.074033
H -0.671836 ; 2.400737 ; 0.699775
H -0.979981 ; 1.901103 ; -0.983259
Ta 0.440270 ; -0.028071 ; -0.204216
C 2.208597 ; 0.119210 ; 0.878166
H 3.188989 ; 0.058808 ; 0.393925
H 2.290968 ; 0.257860 ; 1.955562
****
302
Charge:1 Multiplicity:1 ()
E(Hartree):-253.328459 Zero-point:0.123919
Cartesian coordinates (Angstroms)
Ta -0.110708 ; 0.031809 ; -0.159174
C -1.466732 ; -1.492219 ; -0.060327
H -1.935331 ; -2.359704 ; -0.506299
C -1.560041 ; -0.762300 ; 1.034731
H -2.129400 ; -0.744086 ; 1.955253
C 2.350902 ; -0.869571 ; -0.060019
H 2.172581 ; -1.821958 ; 0.431851
H 2.569980 ; -0.904098 ; -1.124668
C 2.434145 ; 0.278030 ; 0.637906
H 2.318046 ; 0.295564 ; 1.717802
H 2.717498 ; 1.213059 ; 0.162766
C -0.819417 ; 1.985742 ; 0.131475
H -0.204137 ; 2.592375 ; -0.552707
H -0.724143 ; 0.144835 ; -1.791596
H -0.473497 ; 2.146387 ; 1.171279
H -1.863065 ; 2.277449 ; 0.053437
****

$TaC_5H_{12}^+$

Minima

304
Charge:1 Multiplicity:1 (0.0000)
E(Hartree):-254.553985 Zero-point:0.146688
Cartesian coordinates (Angstroms)
Ta 0.151634 ; -0.086764 ; -0.048818
C 1.463179 ; 1.250133 ; 0.740701
H 2.227437 ; 1.511746 ; 1.461257
C 0.831560 ; 1.823953 ; -0.272045
H 0.844705 ; 2.770623 ; -0.795026
H 0.300112 ; -0.028962 ; -1.783846
C 1.580100 ; -1.631127 ; -0.141108
H 1.712948 ; -1.893353 ; 0.925228
H 1.072196 ; -2.470526 ; -0.642610
H 2.563287 ; -1.492851 ; -0.585078

C	-2.573572 ; 0.322499 ; -0.485782
H	-3.051222 ; 1.275463 ; -0.268183
H	-1.736088 ; 0.558998 ; -1.181613
H	-3.246086 ; -0.295931 ; -1.077737
C	-2.193492 ; -0.400884 ; 0.810286
H	-3.065526 ; -0.551397 ; 1.447861
H	-1.804491 ; -1.425087 ; 0.662685
H	-1.533196 ; 0.187609 ; 1.488467
****
305
Charge:1 Multiplicity:1 (0.0000)
E(Hartree):-254.562863 Zero-point:0.148414
Cartesian coordinates (Angstroms)
Ta	0.265438 ; 0.028223 ; -0.018117
C	-2.248268 ; -0.136617 ; 0.207884
H	-2.998450 ; -0.916672 ; 0.332421
H	-2.739796 ; 0.832707 ; 0.230908
C	-1.718751 ; -0.354254 ; -1.266105
H	-2.510516 ; -0.067540 ; -1.959322
H	-1.454808 ; -1.391292 ; -1.495363
C	-1.239610 ; -0.236109 ; 1.418803
H	-1.243063 ; -1.224589 ; 1.882512
H	-1.445967 ; 0.530136 ; 2.160242
C	0.790285 ; 1.870416 ; -0.063512
H	1.743824 ; 2.252251 ; -0.432005
H	0.152856 ; 2.647143 ; 0.366518
C	1.957396 ; -1.251017 ; -0.005867
H	2.257799 ; -1.308723 ; 1.054031
H	-0.931163 ; 0.384269 ; -1.652770
H	2.815429 ; -0.881204 ; -0.574670
H	1.730581 ; -2.271310 ; -0.337193
****
372
Charge:1 Multiplicity:1 ()
E(Hartree):-254.583262 Zero-point:0.145005
Cartesian coordinates (Angstroms)
Ta	0.004692 ; 0.069119 ; 0.000003
C	-0.508995 ; -1.933038 ; 0.000148
H	-0.285120 ; -2.991983 ; 0.000218
C	-1.596177 ; -1.188371 ; 0.000217
H	-2.669758 ; -1.318849 ; 0.000288
C	2.555290 ; -0.351687 ; 0.000134
H	3.468360 ; -0.948698 ; 0.000537
H	2.563493 ; 0.261176 ; -0.905552
H	1.782289 ; -1.159454 ; -0.000581
H	2.562515 ; 0.260609 ; 0.906233
C	-0.554522 ; 1.124827 ; -1.751090
H	-1.476996 ; 1.687556 ; -1.566325
H	0.253753 ; 1.862093 ; -1.903087
H	-0.681469 ; 0.543639 ; -2.665266
C	-0.554723 ; 1.125324 ; 1.750660
H	-0.683008 ; 0.544887 ; 2.665099
H	0.254440 ; 1.861653 ; 1.902799
H	-1.476262 ; 1.689323 ; 1.565028
****
371-1
Charge:1 Multiplicity:1 ()
E(Hartree):-254.60303 Zero-point:0.143920
Cartesian coordinates (Angstroms)
Ta	-0.159674 ; -0.016818 ; -0.000214
C	1.621528 ; -1.090264 ; -0.009430
H	1.811485 ; -2.159799 ; -0.018476
C	2.637614 ; -0.214298 ; -0.001837
H	2.490194 ; 0.867275 ; 0.007293
H	3.682567 ; -0.514549 ; -0.004236
C	-0.242787 ; 1.231192 ; -1.703966
H	-1.272526 ; 1.238772 ; -2.082390
H	0.033485 ; 2.261361 ; -1.450192
H	0.420647 ; 0.879762 ; -2.500546
C	-0.242492 ; 1.201339 ; 1.725064
H	-1.272852 ; 1.204985 ; 2.101806
H	0.418590 ; 0.833669 ; 2.516278
H	0.037200 ; 2.235089 ; 1.490447
C	-1.831209 ; -1.289806 ; -0.010787
H	-1.436434 ; -2.317270 ; -0.018129
H	-2.456565 ; -1.171883 ; 0.880696
H	-2.455486 ; -1.158695 ; -0.901203
****

Transition states

373
Charge:1 Multiplicity:1 ()
E(Hartree):-254.564387 Zero-point:0.141435
Cartesian coordinates (Angstroms)
Ta	0.097177 ; -0.045392 ; -0.000010
C	-0.765183 ; 1.835448 ; 0.000547
H	-0.613315 ; 2.905327 ; 0.000808
C	-1.789070 ; 1.021342 ; 0.000451
H	-1.672371 ; -0.513199 ; -0.000013
H	-2.868513 ; 1.110741 ; 0.000554
C	-1.237149 ; -1.886406 ; -0.000504
H	-0.816827 ; -2.363132 ; -0.895083
H	-0.816792 ; -2.363735 ; 0.893732
H	-2.308028 ; -2.099166 ; -0.000565
C	1.290867 ; 0.047140 ; -1.751171
H	0.765690 ; 0.244412 ; -2.687791
H	2.064925 ; 0.813860 ; -1.622160
H	1.797835 ; -0.927937 ; -1.832800
C	1.291191 ; 0.045942 ; 1.750995
H	0.766341 ; 0.243402 ; 2.687746
H	1.797241 ; -0.929649 ; 1.832333
H	2.065983 ; 0.811907 ; 1.622045
****
